# The Biphasic Effects of Moderate Alcohol Consumption with a Meal on Ambiance-Induced Mood and Autonomic Nervous System Balance: A Randomized Crossover Trial

**DOI:** 10.1371/journal.pone.0086199

**Published:** 2014-01-21

**Authors:** Ilse C. Schrieks, Annette Stafleu, Victor L. Kallen, Marc Grootjen, Renger F. Witkamp, Henk F. J. Hendriks

**Affiliations:** 1 The Netherlands Organization for Applied Scientific Research, TNO, Zeist, The Netherlands; 2 Wageningen University, Wageningen, The Netherlands; 3 EagleScience, Amsterdam, The Netherlands; Centre for Addiction and Mental Health, Canada

## Abstract

**Background:**

The pre-drinking mood state has been indicated to be an important factor in the mood effects of alcohol. However, for moderate alcohol consumption there are no controlled studies showing this association. Also, the mood effects of consuming alcohol combined with food are largely unknown. The aim of this study was to investigate the effects of moderate alcohol combined with a meal on ambiance-induced mood states. Furthermore effects on autonomic nervous system activity were measured to explore physiological mechanisms that may be involved in changes of mood state.

**Methods:**

In a crossover design 28 women (age 18–45 y, BMI 18.5–27 kg/m^2^) were randomly allocated to 4 conditions in which they received 3 glasses of sparkling white wine (30 g alcohol) or alcohol-free sparkling white wine while having dinner in a room with either a pleasant or unpleasant created ambiance. Subjects filled out questionnaires (B-BAES, POMS and postprandial wellness questionnaire) at different times. Skin conductance and heart rate variability were measured continuously.

**Results:**

Moderate alcohol consumption increased happiness scores in the unpleasant, but not in the pleasant ambiance. Alcohol consumption increased happiness and stimulation feelings within 1 hour and increased sedative feelings and sleepiness for 2.5 hour. Skin conductance was increased after alcohol within 1 hour and was related to happiness and stimulation scores. Heart rate variability was decreased after alcohol for 2 hours and was related to mental alertness.

**Conclusion:**

Mood inductions and autonomic nervous system parameters may be useful to evaluate mood changes by nutritional interventions. Moderate alcohol consumption elevates happiness scores in an unpleasant ambiance. However, drinking alcohol during a pleasant mood results in an equally positive mood state.

**Trial Registration:**

Clinicaltrials.gov NCT01426022.

## Introduction

Alcoholic beverages are amongst the oldest beverages and are commonly consumed because of their anticipated mood effects. Motivation for moderate alcohol consumption may vary from enhancing pleasure to reducing tension [1], [2]. Mood and emotions are distinct phenomena, although the two are closely related. Whereas emotions are of short duration and often directed towards an object, moods are generally lasting longer and not object-related. Mood comprises a mixture of hedonic (pleasure-displeasure) and arousal (sleepy-activated) values [3].

The effect of moderate alcohol consumption on mood may depend on several interacting factors, including the rise or decline of blood alcohol concentration (BAC) [4]–[6], the pre-drinking mood state of the drinker [7] and factors such as the social context of drinking [8], [9], and expectancy of mood effects [10], [11].

Alcohol has been shown to change mood in a biphasic pattern. During rising BACs alcohol has stimulating effects, reported by feelings of euphoria and elation, while during declining BACs alcohol has sedative effects, reported by relaxation and sleepiness [4]–[6]. In Western countries alcohol is often consumed with a meal. Food consumption may also affect mood. Previous studies showed an increased calmness and sleepiness after food intake [12]–[15]. Furthermore, food consumption may influence the mood effects of alcohol because it delays gastric emptying and alcohol absorption, resulting in a lower peak BAC [16], [17]. Thus, the combination of food and alcohol consumption may affect mood differently from that of alcohol consumption only. To date, only two studies investigated the mood effects of combined food and alcohol consumption, and their results were inconsistent [18], [19]. Lloyd and Rogers (1997) found that alcohol with a meal improved mood by increasing confidence and decreasing tension and confusion, while Markus et al. (2004) did not find any effect on mood.

Furthermore, the pre-drinking mood state has been indicated to play an important role in the mood effects of alcohol [7], [11]. However, the effects of different pre-drinking moods on postprandial mood changes have not yet been compared in an intervention study. The most accurate method to measure mood is by self-report of feelings as mood is a subjective state. Nevertheless, specific physiological parameters associated with mood changes are increasingly being used in studies generally to validate subjective outcomes or to explore physiological mechanisms involved in mood regulation. However, for yet another reason this may be highly relevant, as incorporating the physiological dynamics underlying both mood effects and pharmacological activities of psychotropic substances like alcohol may be instrumental to further unravel the effects of such enhancers on mood. With respect to alcohol, the relevant physiological parameters are likely to include measures of autonomic nervous system (ANS) activity as moderate alcohol consumption has been shown to increase the activity of the sympathetic division of the ANS (being related to emotional arousal), and to decrease the activity of the parasympathetic division of the ANS, associated with relaxation [10], [20]–[25].

The primary aim of this study was to evaluate the biphasic effects of moderate alcohol consumption with a meal on ambiance-induced mood, as measured by subjective responses and ANS activity. The secondary aim was to study the association between the effects of moderate alcohol consumption on subjective responses of mood and on ANS activity. We hypothesized that moderate alcohol consumption with a meal would amplify the pre-drinking mood state, resulting in a more positive mood in a positive induced mood state and in a more negative mood in a negative induced mood state. Furthermore, the induced mood was expected to influence the effect of alcohol on ANS activity, specifically by decreasing sympathetic outflow and increasing parasympathetic outflow in a positive induced mood and opposite effects in a negative induced mood.

## Materials and Methods

### Ethics Statement

The study was conducted at TNO (The Netherlands Organization for Applied Scientific Research) in Zeist, The Netherlands, and was performed according to the International Conference on Harmonisation Guidelines for Good Clinical Practice. The study also complied with the Declaration of Helsinki and was approved by an independent Medical Ethics Committee (METOPP, Tilburg, The Netherlands). Written informed consent was obtained from all subjects. The study is registered at ClinicalTrials.gov (NCT): NCT01426022. The protocol for this trial and supporting CONSORT checklist are available as supporting information (Protocol S1 and Checklist S1).

### Subjects

**Figure 1:**
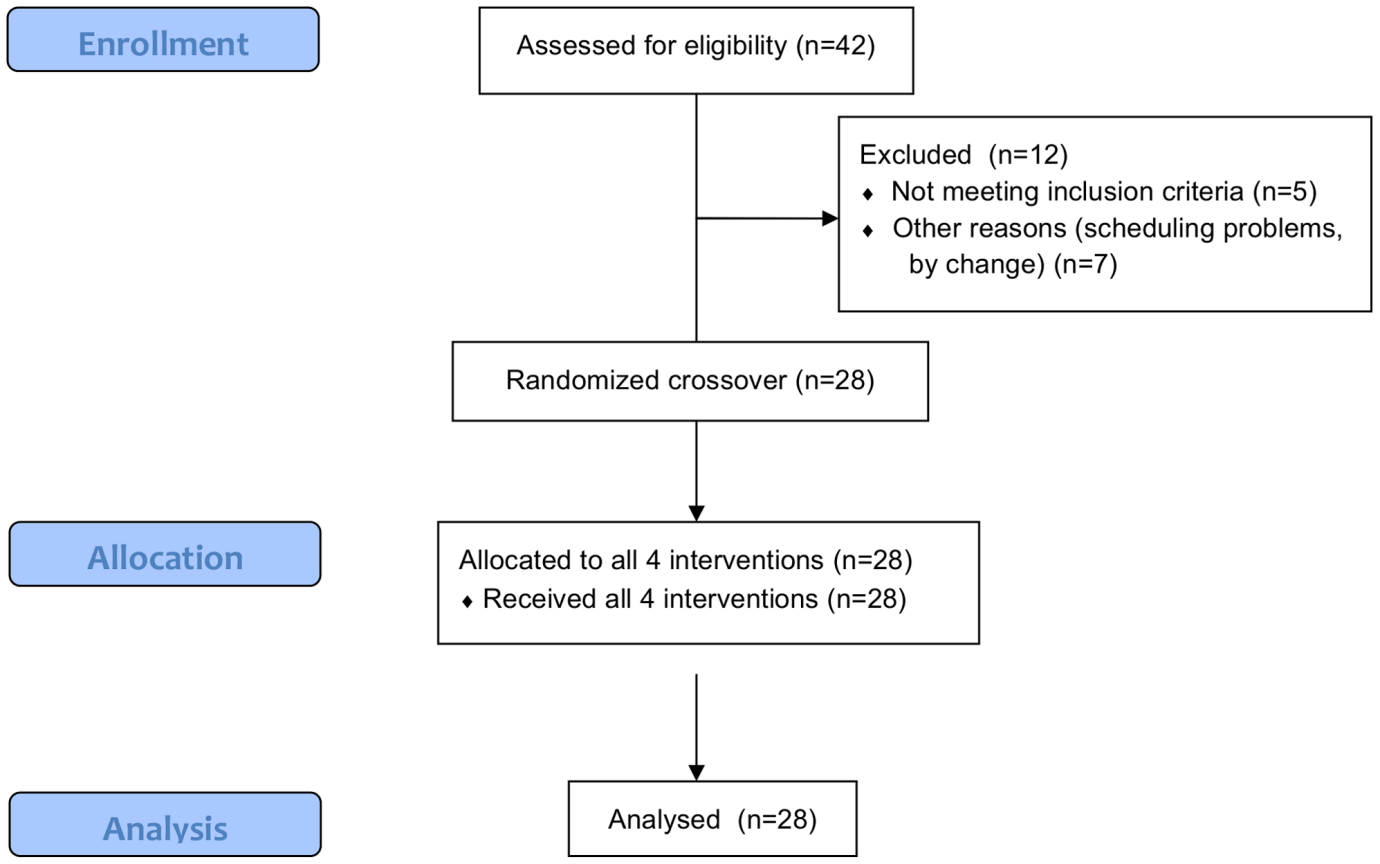
Flow chart (CONSORT).

Healthy women (28 women aged 18–43 years, BMI 22.1±1.7 kg/m^2^) participated in the study (Figure 1). The subjects were recruited from a pool of volunteers at TNO in Zeist, The Netherlands. Eligible subjects did not use any medication, habitually consumed alcohol (3–20 glasses/week) and had no (family) history of alcoholism. We chose women who were taking oral contraceptives, thus expecting to reduce possible effects of the menstrual cycle on mood. They were not tested in the week they were not taking oral contraceptives. The calculated sample size (power analysis) was 24 subjects, where α was 0.05 (two-sided), β was 0.80 and the effect size was 1.2, based on a previous study with Profile of Mood States (POMS) as outcome measure [14]. Twenty-eight subjects were included to guarantee sufficient power, even in case drop out may occur.

### Experimental protocol

The study used a randomized single-blind crossover design, with the intervention factors alcohol and ambiance. Subjects consumed 3 glasses of sparkling white wine (30 g alcohol; Prosecco Santa Chiara, Italy) or alcohol-free sparkling white wine (<2 g alcohol; Vita Nova Sparkling Secco, The Netherlands) with a meal in either a pleasant or an unpleasant ambiance. The blood alcohol concentration was expected not to exceed 0.5‰, which is the Dutch legal limit of driving. Therefore, we considered consumption of 30 g alcohol together with a meal as a moderate dose.

**Figure 2:**
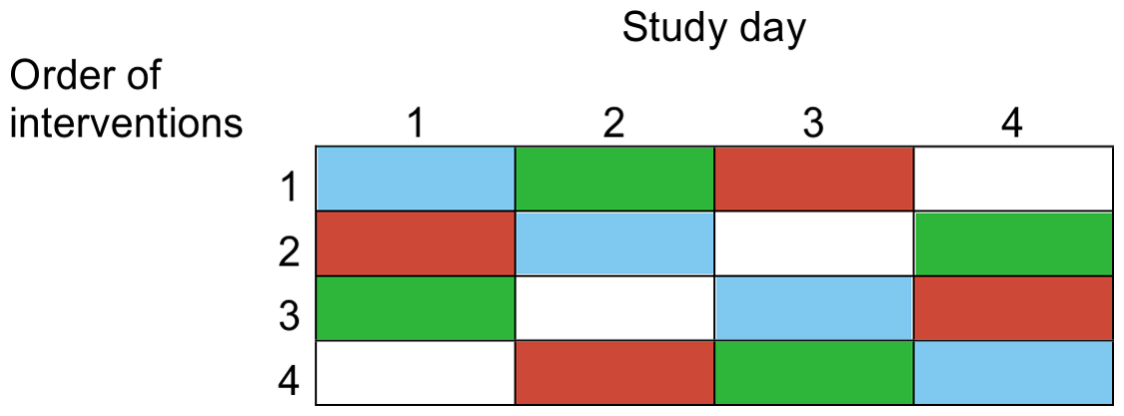
Randomization of the interventions according to a Latin Square design. Blue: Unpleasant ambiance +3 glasses of sparkling white wine Green: Pleasant ambiance +3 glasses of sparkling white wine Red: Unpleasant ambiance +3 glasses of alcohol-free sparkling white wine White: Pleasant ambiance +3 glasses of alcohol-free sparkling white wine.

Each subject participated in all 4 experimental conditions, which occurred at least one week apart. Subjects were equally divided in 4 groups with different intervention orders according to a Latin square design (Figure 2). Allocation to intervention order was randomized according to body fat percentage and age by a computer-generated randomization scheme. Randomization and intervention order allocation were performed by statisticians of TNO.

Subjects were kept ignorant to the ultimate study aim; instead they were informed that the aim was to investigate the effect of different meal settings and alcohol on hormones and satiety. In addition, they were informed that the alcohol content of the beverages could vary per intervention day. Subjects were blinded to the alcohol intervention. They were instructed to refrain from drinking alcohol on the evening preceding each test day, to eat their standard breakfast and lunch on standard times on each test day and to refrain from eating and drinking anything except water 2 hours before testing. After connecting the electrodes for skin conductance and electrocardiography (ECG) measurements, a baseline measurement was performed for 2 min in a seated position. From that moment onwards, skin conductance and ECG were collected continuously until the end of the study day. Subjects relaxed for at least 15 min in a room where soft music was played before they went to the test rooms with either a pleasant or an unpleasant ambiance. In these rooms, subjects filled out computer questionnaires about their mood. Afterwards, they were asked to consume their first glass of sparkling white wine or alcohol-free white wine within 5 min. This moment is considered t = 0. Immediately following their wine consumption the participants were served a meal consisting of a macaroni dish (2004 kJ, Apetito B.V., Denekamp, The Netherlands) with two more glasses of wine. The meal and 2 glasses of wine had to be finished in 15 min. BAC was measured with a breathalyzer (Alcotest 7410, Dräger Nederland, Zoetermeer, The Netherlands) before the first drink and 30 min, 60 min, 120 min and 150 min afterwards. Mood questionnaires were filled out before the first drink and at 20 min, 50 min, 110 and 140 min after. After the last questionnaire, a second baseline (recovery) measurement of 2 min was carried out for skin conductance and ECG.

Minor changes were made in the study procedure after the second study day to reduce potential stress from e.g. number of blood collections. These changes were approved by the Medical Ethics Committee METOPP.

### Mood induction by meal ambiance

Rooms with a pleasant or an unpleasant meal ambiance were created by environmental factors as lighting, music, cleanness, decoration and a film scene. The pleasant ambiance room was colourfully decorated, light was soft, and music was playing. The unpleasant ambiance was created by having very bright lighting, no music, a filled dustbin next to the table, no decoration and plastic cutlery and serving dish. Ambient temperature was similar in both rooms. The ambiances were enhanced by showing the participants either a happy or sad film scene from the animation films ‘Bambi’ (Walt Disney, 1942) and ‘The Lion King’ (Walt Disney, 1994). The scenes were approximately 2.5 min long and were shown during the first glass of wine or alcohol-free wine. The happy scenes were either the scene of ‘Bambi on the ice’ or the Lion King scene of ‘hakuna matata’. The sad scenes were the scene of ‘Bambi's mother dying’ [26] or the ‘Lion Kings father dying’ [27]. Subjects watched each film scene once. Subjects had dinner individually and stayed in the room until all measurements were completed.

### Mood questionnaires

Mood was measured with three different questionnaires, which were filled out on the computer using adaptive VAS software [28]. Subjects practised the questionnaires once to familiarize.

#### Profile of Mood States (POMS)

Changes in mood were measured using the short version of the ‘Profile of Mood States’ (POMS) questionnaire [29]. The questionnaire was computer based and asked participants to answer questions on a five-point interval scale ranging from ‘strongly disagree’ to ‘strongly agree’. The POMS comprises 40 items for five different subscales for mood. The subscale Anger (7 items, score range 7–35), Depression (8 items, score range 8–40), Fatigue (6 items, score range 6–30) and Tension (6 items, score range 6–30) refer to a negative mood state, whereas the subscale Vigor (5 items, score range 5–25) refers to a positive mood state. We added two more positive mood subscales, Happiness (4 items, score range 4–20) and Calmness (4 items, score range 4–20) from the Brunel mood scale to make the questionnaire more balanced for negative and positive moods [30].

#### Postprandial wellness questionnaire

The postprandial wellness questionnaire has been developed and described by Boelsma et al. (2010) to measure the combined effects of satiety feelings and wellness after a meal (visual analogue scale, 100-unit) [15]. The recorded items were pleasantness, satisfaction, relaxation, sleepiness, physical energy, mental alertness, hunger, fullness, desire to eat and prospective food consumption. We added the items experienced body temperature and thirst.

#### Biphasic Alcohol Effects Scale (B-BAES)

The stimulation and sedative effects of alcohol were measured with the brief version of the Biphasic Alcohol Effects Scale (B-BAES) [31], [32]. The questionnaire comprises of two subscales, sedation and stimulation, with three items per subscale. The participants were asked to indicate ‘the extent to which the adjectives described their feelings’ on a visual analogue scale (100-unit) from ‘not at all’ to ‘extremely’. The questions were translated to Dutch and participants had to answer them on a visual analogue scale instead of on an 11-point scale as in the original questionnaire. The B-BAES stimulation and sedation scores were calculated as the average of the scores on the three items within the subscale.

### Autonomic nervous system measurements

Skin conductance and ECG data were collected and stored using a wireless multi-channel ambulatory system (Mobi-8, TMS International, The Netherlands), designed to measure different electro-physiological signals at the same time in both ambulatory and stationary conditions. Data were analysed with BioTrace + Software for NeXus version 1.2.0.0 (Mind Media B.V., The Netherlands).

#### Skin conductance level

As primary parameter of sympathetic activity, skin conductance level was measured continuously with two Ag/AgCL electrodes, which were positioned on the middle phalanx of the ring finger and on the middle phalanx of the index finger of the non-dominant hand after removal of any hand jewelry. Mean skin conductance level was calculated from 2 min time frames. The same frames were used for calculation of the ECG time-domain derivatives.

#### Heart rate variability parameters

Heart rate and heart rate variability parameters were measured using continuous ECG monitoring. Three electrodes were attached on the upper body for the ECG measurement (pre-cordial lead, sampling rate 512 Hz).

From the original ECG time series, inter beat intervals were calculated from R-R intervals. These are the intervals between two successive R-spikes of the QRS-complex in the ECG. Based on the inter beat intervals, the heart rate and root mean square of successive differences (RMSSD) were calculated. RMSSD is a time-domain measure that reflects the short-term variation of the inter beat intervals and is strongly related to parasympathetic nervous system activity [33], [34]. In addition, frequency-domain measurements were determined from a power spectral density analysis computed by a 1024 point Fast Fourier Transformation (FFT) on the inter beat intervals. The following frequency-domain measurements were calculated: low frequency power (LF power, 0.04–0.15 Hz), high frequency power (HF power, 0.15–0.40 Hz) and the LF:HF ratio. HF power is an index of parasympathetic nervous system activity and strongly related to RMSSD. Both are mainly influenced by vagal activity. Heart rate variability analysis was performed according to the Task Force Guidelines [35]. Time frames of 6 min were selected at time points when the questionnaires were filled out (4 time frames at −5 min, 25 min, 55 min and 115 min) to be able to compare the outcomes of the questionnaires with the physiological responses. Each 6 min time frame was subdivided in 2 min time frames for calculation of skin conductance, heart rate and the time-domain heart rate variability parameter RMSSD. Inter beat intervals were manually checked and segments with ectopic beats were not used for analysis. As FFT's mathematically require longer time series (e.g. as compared to time domain based parameters like RMSSD), heart rate variability parameters quantified within the frequency domain were calculated from the 6 min time frames. Consequently, skin conductance level, heart rate and RMSSD were calculated from 2 min time frames, whereas LF power, HF power and LF:HF ratio were calculated from 6 min time frames.

### Data analysis

Statistical analyses were performed using the SAS statistical software package (SAS version 8; SAS Institute, Cary, NC, USA). All variables were compared between interventions with a mixed analysis of variance model that included the fixed factors alcohol (alcohol vs. alcohol-free), ambiance (pleasant vs. unpleasant ambiance), time and the interaction between alcohol and ambiance, time and alcohol, time and ambiance, and time, alcohol and ambiance. The factors subject and subject by period were added to the model as random factors. A post hoc Tukey-Kramer test was used if an intervention effect occurred, to correct for multiple testing. Pearson correlations were calculated within one of the four conditions (Figure 2). Changes over time in subjective feelings were correlated with changes over time in autonomic nervous system measurements for each subject. A Fisher's *z* transformation was applied on the individual correlations, to correct for deviations from the normal distribution and a 95% confidence interval was calculated.

The measurements on the first study day of the first 11 subjects were considered not valid because of logistic problems that occurred, and were therefore excluded from the analyses. Except for baseline characteristics, all values are expressed as means and standard errors of the mean. Error bars in figures express standard errors of the mean.

## Results

### Subject baseline characteristics

Subjects were recruited and enrolled in the trial between September and December 2011 (Figure 1). Baseline characteristics of the participants are shown in Table 1.

**Table 1:** Baseline characteristics of the participants (n = 28).

Variable	Value^a^
Age (y)	22 [18]–[43]
Alcohol consumption	
3–6 drinks/week	61%
7–14 drinks/week	36%
15–21 drinks/week	4%
Body weight (kg)	66±6
BMI (kg/m^2^)	22.1±1.7
Body fat percentage (%)	24.5±5.5
Heart rate (beats/min)	66±9
Systolic blood pressure (mmHg)	109±9
Diastolic blood pressure (mmHg)	72±8

a Data are expressed as median and [range] for age, percentage for alcohol consumption and means ± SD for the other variables.

BMI: body mass index.

### Mood induction by ambiance

The study was designed to induce a positive and a negative mood by ambiance. The results of the questionnaires show that mood was influenced by ambiance, with a more positive mood in the pleasant ambiance than in the unpleasant ambiance. Scores on the subscales anger, tension and depression of the POMS were all higher in the unpleasant ambiance than in the pleasant ambiance (all *P*<0.05), with depression increasing over time in the unpleasant ambiance (*P*<0.05). The subscale happiness of the POMS and the items pleasantness, relaxation and mental alertness of the postprandial wellness questionnaire were all higher in the pleasant ambiance (*P*<0.05, P<0.001, *P*<0.01, *P*<0.05, respectively). There was no main effect of ambiance on the B-BAES subscales stimulation and sedation, although sedation scores were 10% lower in the pleasant ambiance (*P* = 0.05).

### Effects of alcohol on mood

The hypothesis was that moderate alcohol consumption would amplify the ambiance-induced mood.

#### Main effects of alcohol

**Figure 3:**
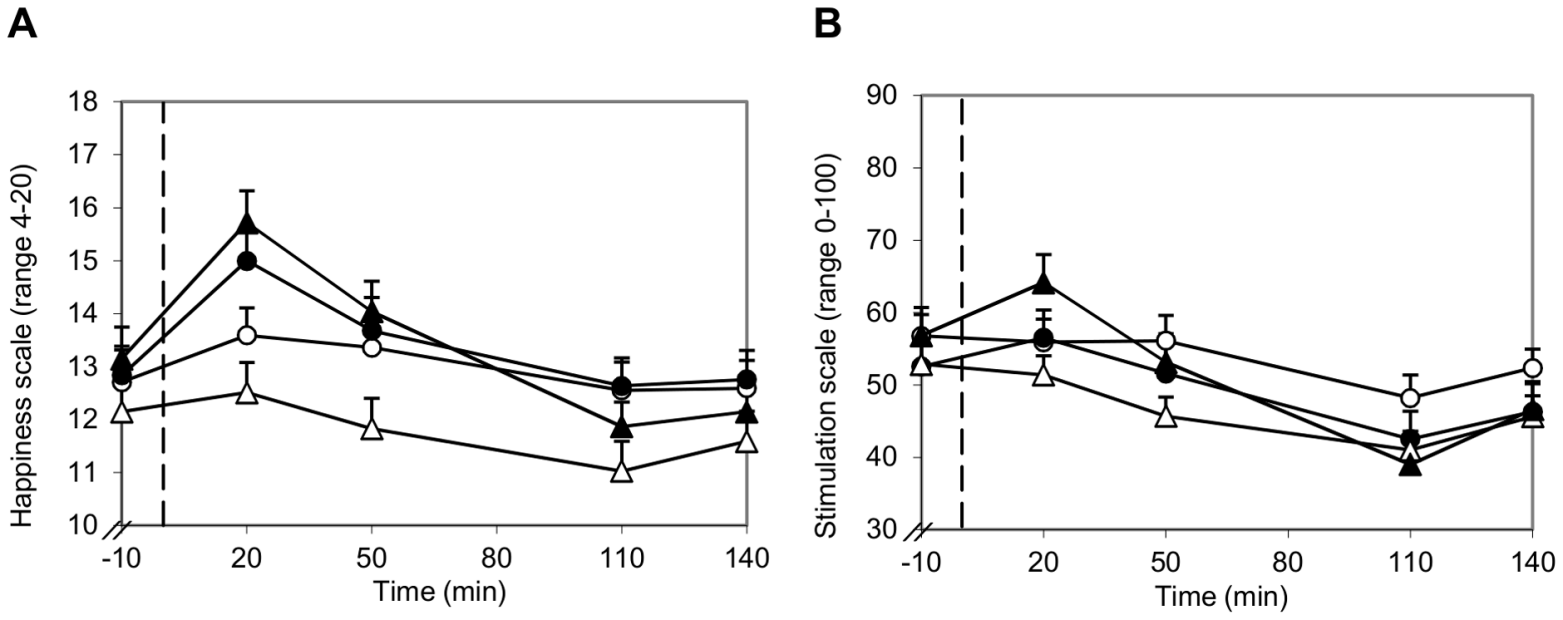
Influence of ambiance and alcohol on POMS happiness scores (A) and B-BAES stimulation scores (B). −○− Pleasant, alcohol-free; −•− Pleasant, alcohol; −Δ− Unpleasant, alcohol-free; −▴− Unpleasant, alcohol; - - start alcohol consumption (t = 0). (**A**) Happiness scores were higher 20 min and 50 min after alcohol consumption compared to no alcohol consumption (*P*<0.001 and *P*<0.05, respectively). Happiness scores were lower in the unpleasant ambiance without alcohol than in the pleasant and unpleasant ambiance with alcohol (*P*<0.001) and in the pleasant ambiance without alcohol (*P*<0.05). (**B**) Stimulation scores were decreased from 20 min until 50 min after alcohol consumption, which did not occur after consumption of alcohol-free drinks (*P*<0.05 vs. *P* = 1.0). Stimulation scores tended to be higher in the pleasant ambiance without alcohol than in the unpleasant ambiance without alcohol (*P* = 0.08).

**Figure 4:**
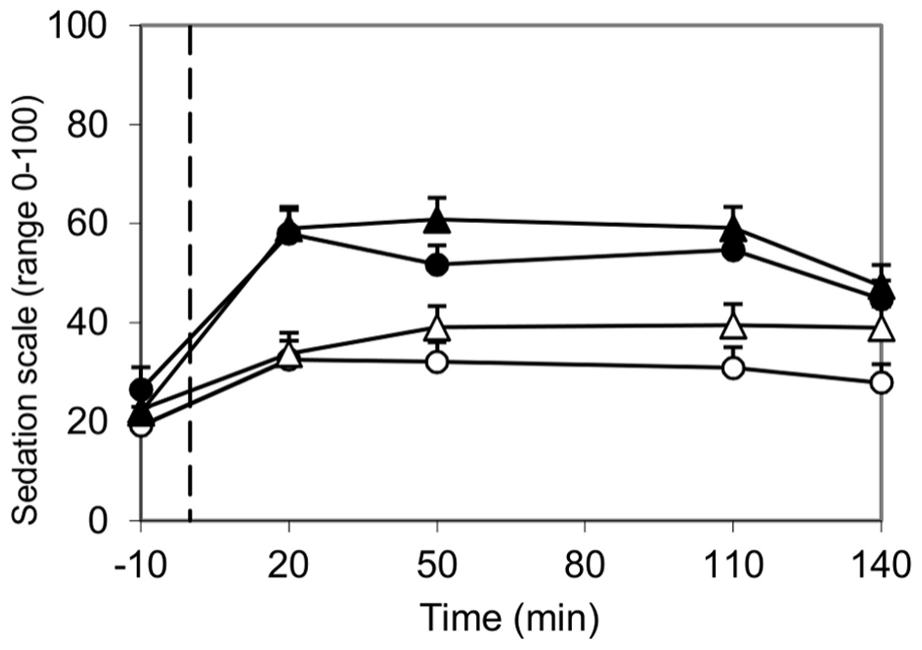
Influence of ambiance and alcohol on B-BAES sedation scores. −○− Pleasant, alcohol-free; −•− Pleasant, alcohol; −Δ− Unpleasant, alcohol-free; −▴− Unpleasant, alcohol; - - start alcohol consumption (t = 0). Sedation scores were higher after alcohol consumption compared to no alcohol consumption at 20 min, 50 min, 110 min (all *P*<0.001) and 140 min (*P*<0.01) after consumption . Sedation scores tended to be higher in the unpleasant ambiance (*P* = 0.05).

Moderate alcohol consumption with a meal influenced mood, but these effects were different over time. Acute effects of alcohol (within 1 hour) were increased happiness and stimulation scores (Figure 3, *P*<0.001 and *P*<0.05) and reduced calmness and mental alertness scores (both *P*<0.001). Effects of alcohol consumption occurring or continuing after 1.5 hour were increased sleepiness (*P*<0.05) and sedation scores (Figure 4, *P*<0.01). These results show time-dependent effects of moderate alcohol consumption with a meal on mood, with a more positive and active mood state within 1 hour after alcohol and food consumption and a more sedated and inactive mood state occurring or continuing after 1.5 hour after alcohol and food consumption. The mean BAC was highest 30 min after alcohol consumption (0.53±0.01‰). Compared to 30 min, BAC was decreased 60 min and 120 min after alcohol consumption (0.47±0.01‰, 0.30±0.01‰ and 0.24±0.01‰ respectively). Thus, the positive and activation effects of alcohol on mood were more present during rising BACs, while the sedation and inactivation effects of alcohol on mood were more present during declining BACs.

#### Interaction effects of ambiance and alcohol

The effect of ambiance on mood was influenced by the alcohol intervention for the POMS happiness subscale and the B-BAES stimulation subscale (Figure 3). Happiness scores were higher when alcohol was consumed in the unpleasant ambiance than when no alcohol was consumed in both the unpleasant and pleasant ambiance (16 vs. 13 and 14 respectively, both *P*<0.001). Stimulation scores were 14% higher when no alcohol was consumed in the pleasant ambiance than in the unpleasant ambiance (54 vs. 47, *P* = 0.08). There were no interaction effects between ambiance, alcohol and time.

### Autonomic nervous system measurements

#### Skin conductance level

**Figure 5:**
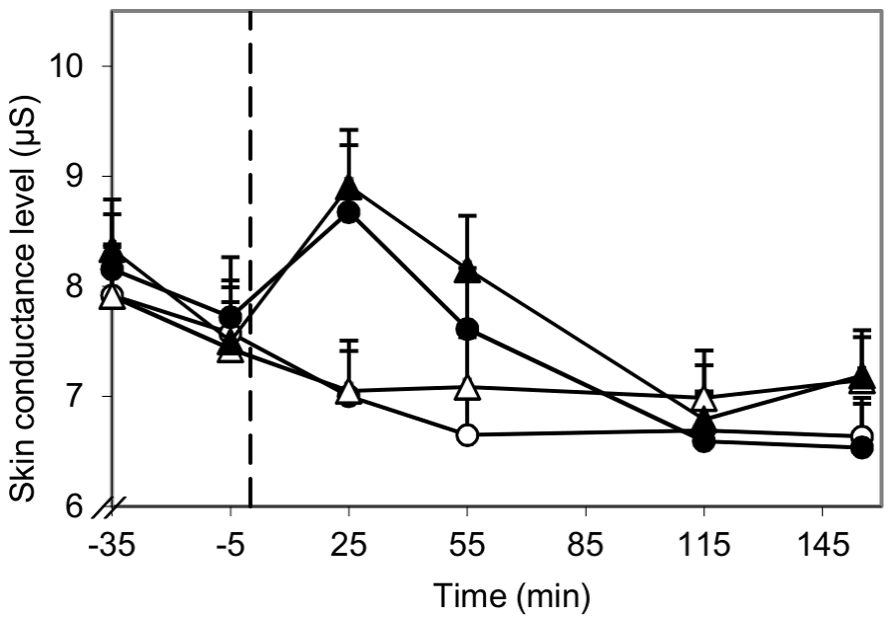
Influence of ambiance and alcohol on skin conductance. −○− Pleasant, alcohol-free; −•− Pleasant, alcohol; −Δ− Unpleasant, alcohol-free; −▴− Unpleasant, alcohol; - - start alcohol consumption (t = 0). Skin conductance was higher 25 min after alcohol consumption compared to no alcohol consumption (*P*<0.001). Skin conductance decreased more from 25 min until 155 in the pleasant ambiance than in the unpleasant ambiance (*P* = 0.042). The values shown at time point −5, 25, 55 and 115 are averages of the mean skin conductance levels measured during three succeeding 2 min time frames.

Skin conductance acutely increased 25 min after alcohol consumption (Figure 5). At this point skin conductance was 25% higher than after consumption of alcohol-free drinks (8.8 µS vs. 7.0 µS respectively; *P*<0.001). In the pleasant ambiance skin conductance decreased more during the time interval from 25 min until 155 min after consumption than in the unpleasant ambiance (*P*<0.05). The increased skin conductance during the first hour after alcohol consumption suggests an increase in sympathetic nervous system activity, with a gradual decline afterwards that might be associated with our findings on the sedation and inactivation effects of alcohol.

#### Heart rate variability parameters

**Figure 6:**
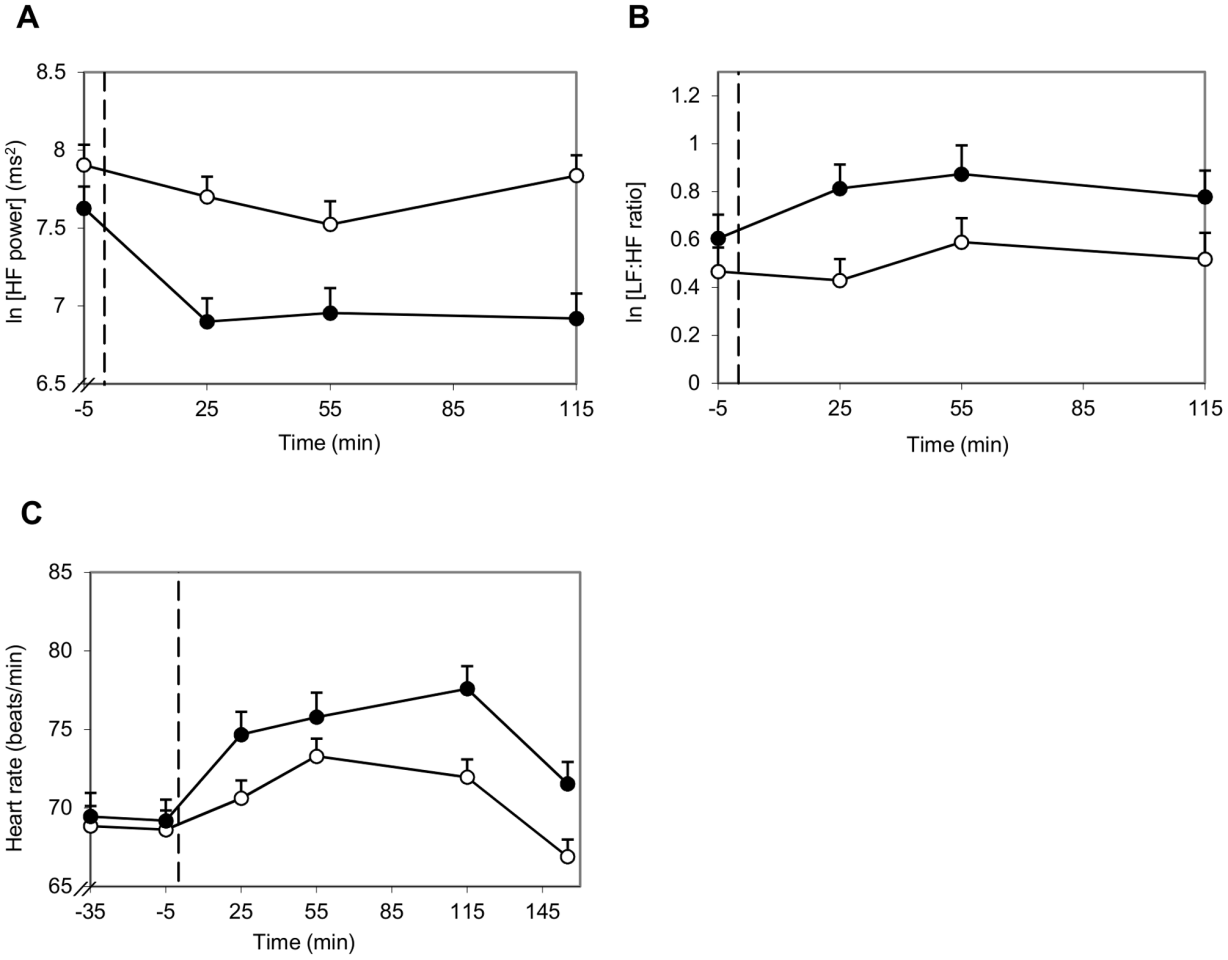
Influence of alcohol on HF power (A), LF:HF ratio (B) and heart rate (C). −○− Alcohol-free; −•− Alcohol; - - start alcohol consumption (t = 0). (**A**) HF power was lower after alcohol consumption compared to no alcohol consumption (at 25 min and 115 min *P*<0.001, at 55 min *P*<0.01). (**B**) LF:HF ratio was higher after alcohol consumption (*P*<0.001). (**C**) Heart rate was higher 25 min and 115 min after alcohol consumption compared to no alcohol consumption (*P*<0.01 and *P*<0.001, respectively). The heart rate values shown at time point −5, 25, 55 and 115 are averages of the mean heart rates measured during three succeeding 2 min time frames.

Heart rate variability responses after consumption of alcohol or alcohol-free drinks with a meal are shown in Figure 6. After moderate alcohol consumption ln [HF power] was decreased during all time points (25 min until 115 min after consumption) compared to no alcohol consumption (*P*<0.001, *P*<0.01 and *P*<0.001, respectively). After 115 min, ln [HF power] was still 12% lower in the alcohol intervention group than in the alcohol-free intervention group. RMSSD was similarly influenced by alcohol as HF power and showed a very similar response, with lower RMSSD 25 min, 55 min and 115 min after alcohol consumption than after consumption of alcohol-free drinks (*P*<0.001, *P*<0.01 and *P*<0.001, respectively). The decrease in HF power and RMSSD after alcohol consumption suggests an alcohol induced parasympathetic suppression.

The LF:HF ratio was higher when alcohol was consumed than when no alcohol was consumed (*P*<0.001). HF power, RMSSD and LF:HF ratio were not influenced by ambiance. Heart rate was also increased by alcohol consumption, with higher values 25 min and 115 min after alcohol consumption than after consumption of alcohol-free drinks (*P*<0.001). The heart rate response was different in the pleasant ambiance than in the unpleasant ambiance (*P*<0.001). Until 55 min after consumption, heart rate was higher in the pleasant ambiance, but from 110 min onwards heart rate was higher in in the unpleasant ambiance. However, differences were very small. The increased LF:HF ratio after alcohol consumption indicates a sympathetic overtone in the balance between sympathetic and parasympathetic activity that does not recover within 2 hours.

### Relation between autonomic nervous system parameters and subjective feelings

Associations were calculated between changes in autonomic nervous system parameters and changes in subjective feelings to examine whether these were related. If correlations were higher than *r* = 0.5 or lower than *r* = −0.5 and if *r* = 0.0 was not part of the confidence interval than they were assumed significantly enough to describe (Table 2).

**Table 2:** Correlations between autonomic nervous system parameters and subjective feelings (n = 28)^1^.

Correlated variables	Intervention	Correlation	95% CI
**SNS parameter – subjective feeling**			
SC – Stimulation (B-BAES)	Alcohol, pleasant	0.60	(0.29, 0.79)
SC – Stimulation (B-BAES)	Alcohol, unpleasant	0.63	(0.34, 0.81)
SC – Calmness (POMS)	Alcohol, pleasant	−0.51	(−0.74, −0.07)
SC – Calmness (POMS)	Alcohol, unpleasant	−0.66	(−0.83, −0.37)
SC – Happiness (POMS)	Alcohol, unpleasant	0.75	(0.52, 0.87)
SC – Fatigue (POMS)	Alcohol, unpleasant	−0.51	(−0.74, 0.17)
SC – Anger (POMS)	No alcohol, unpleasant	0.51	(0.16, 0.74)
SC – Pleasant (PPW)	Alcohol, pleasant	0.54	(0.21, 0.76)
SC – Experienced BT (PPW)	Alcohol, unpleasant	0.52	(0.19, 0.75)
**PNS parameter – subjective feeling**			
HF power – Sedation (B-BAES)	Alcohol, unpleasant	−0.61	(−0.80, −0.31)
HF power – Mental alertness (PPW)	Alcohol, unpleasant	0.62	(0.33, 0.81)
HF power – Experienced BT (PPW)	Alcohol, pleasant	−0.51	(−0.74, −0.17)
HF power – Experienced BT (PPW)	Alcohol, unpleasant	−0.58	(−0.78, −0.26)
HF power – Experienced BT (PPW)	No alcohol, pleasant	−0.52	(−0.75, −0.18)
HF power – Experienced BT (PPW)	No alcohol, unpleasant	−0.55	(−0.77, −0.22)

1 All values are mean [correlation coefficient (*r*)] and 95% confidence interval after Fisher's *z* transformation. Correlations are shown when *r* = 0.0 is not part of the confidence interval and when *r*>0.5 or *r*<−0.5.

Abbreviations: SNS: sympathetic nervous system; PNS: parasympathetic nervous system; SC: skin conductance; HF power: high frequency power; B-BAES, brief biphasic alcohol effects scale; POMS, profile of mood states; PPW: postprandial wellness questionnaire; BT: body temperature.

Skin conductance, the index for sympathetic activity, was positively related with the stimulation subscale and negatively related with calmness after alcohol consumption in both the pleasant and unpleasant ambiance. Skin conductance was positively related to happiness and experienced body temperature and negatively related to fatigue in the unpleasant ambiance with alcohol.

HF power and RMSSD are two heart rate variability parameters that are related to parasympathetic activity. In Table 2 only HF power is shown, but RMSSD showed similar correlations with the listed subjective items. In the unpleasant ambiance with alcohol, HF power and RMSSD were negatively related to the sedation subscale and positively related to mental alertness. HF power and RMSSD were associated with experienced body temperature in all conditions, except for the pleasant ambiance with alcohol.

## Discussion

The present study provided two major findings on the role of pre-drinking mood state in alcohol-induced mood changes and the possible involvement of the ANS. First, alcohol did not amplify ambiance-induced mood, but it improved mood in the unpleasant ambiance as shown by increased scores for feelings of happiness. Second, parameters for ANS activity were influenced by alcohol consumption, but not by ambiance-induced mood, except for some minor changes in skin conductance. Although no effects of pre-drinking mood state were found on ANS activity, ANS parameters were associated to subjective feelings of high or low arousal after alcohol consumption. This indicates that ANS parameters can be used as objective measures of the arousal dimension of mood.

We hypothesized that alcohol would amplify the ambiance-induced mood, but the opposite was found; in a more negative mood state, created by an unpleasant ambiance, moderate alcohol consumption with a meal increased self-reported happiness feelings. In the pleasant ambiance mood was not further improved by alcohol. Alcohol did not affect any other mood scales that were influenced by ambiance. With the exception of happiness, alcohol and ambiance influenced different mood scales.

We successfully induced a positive and negative mood state by creating rooms with either a pleasant or an unpleasant ambiance. In the pleasant ambiance, subjects scored higher on pleasantness and relaxation and lower on depression and tension than in the unpleasant ambiance. This indicates that subjects were indeed in a better mood in the pleasant ambiance than in the unpleasant ambiance. This is in accordance with previous research showing that mood declines when there is no color, and when light is too bright, similar to the conditions of the unpleasant ambiance in our study [36]. The mood effects of ambiance were present as long as the subjects were in the rooms, which was almost 3 hours. Other mood induction methods used previously, such as images, music, film clips and personalized recall, generally affect mood for a much shorter period [27], [37]–[39]. Therefore, the mood induction by ambiance used in this study may be more useful for intervention studies in which a longer mood induction is preferred, such as experiments on food intake and eating behavior. Ambiance has been shown previously to influence eating behavior. This is probably caused by mood changes, although mood effects were not evaluated in these studies [40]–[42].

Moderate alcohol consumption with a meal resulted in biphasic mood effects. During the first hour after wine consumption subjects reported higher happiness and stimulation and lower calmness and mental alertness than after consumption of alcohol-free wine. During the first hour following alcohol consumption also sedative feelings were increased, but these effects remained elevated until the last measurement. This biphasic pattern differs from studies in which alcohol is consumed without food in the early-late afternoon. Those studies found that sedative effects arose on the descending limb of the BAC curve, while we observed sedative effects directly after consumption [31], [43], [44]. However, Addicot et al. (2007) also observed sedative effects before BAC levels started to decline [45]. The earlier onset of sedative feelings in the present study might be due to an increase in tiredness caused by the food intake. Indeed, in the alcohol-free condition, fatigue scores were increasing and vigor scores were decreasing after the meal. Lloyd & Rogers (1997) and Markus et al. (2004) also measured the influence of combined alcohol and food intake on mood [18], [19]. Lloyd & Rogers (1997) observed no effects of moderate alcohol consumption on elation, energy and tiredness, which is in contrast to our findings. An explanation may be the lower number of subjects in the study of Lloyd & Rogers (1997) [18]. Markus et al. (2004) did not find any mood effects of a moderate dose of alcohol (40 g) with an evening meal. However, they measured mood only once, 2 h after consumption [19]. Therefore, the study designs of both studies may not have had enough power to detect differences.

Motives to drink a moderate amount of alcohol are mostly to enhance mood or for social reasons, but with heavy alcohol consumption motives as coping with stress or negative mood also occur frequently. Although in the present study mood effects of moderate alcohol consumption were measured, we realize that coping motives in heavy alcohol consumption are related to problem drinking and addiction [1], [2].

The second hypothesis was that alcohol consumption in a positive pre-drinking mood would cause higher heart rate variability and lower skin conductance than in a negative pre-drinking mood. This hypothesis, however, is not confirmed by our results, as we did not find interaction effects of alcohol and ambiance on ANS activity.

Ambiance only had a small influence on ANS activity. Skin conductance was further declined 2.5 h after consumption in the pleasant ambiance compared to the unpleasant ambiance, but heart rate variability parameters were not affected by ambiance. The small influence of ambiance on ANS activity may be due to the fact that varying ambiances resulted in mood changes. In contrast to emotional changes, mood changes are suggested to have no influence on ANS activity, because mood is not related to an object and no action from the body is required [25].

Moderate alcohol consumption influenced ANS activity. Compared to the situation in which no alcohol was consumed, alcohol increased skin conductance within the first hour, which indicates a short-term increase in sympathetic activity. On the other hand, alcohol consumption decreased heart rate variability (RMSSD and HF power), indicating a decrease in parasympathetic tone and vagal withdrawal. As a result, LF:HF ratio and heart rate, indices of sympathovagal balance, were increased after alcohol consumption.

These results are in accordance with previous research on the effect of alcohol on heart rate variability [10], [20]–[22]. Skin conductance was also increased after alcohol consumption in a study by Levenson et al (1980) [10]. However, Stritzke et al (1995) found an attenuated skin conductance response after alcohol consumption when subjects watched either pleasant or unpleasant pictures inducing arousal [46]. In contrast to previous studies, in the present study alcohol was combined with food consumption. Autonomic activity plays an important role in energy balance and thermoregulation. Sympathetic stimulation increases energy metabolism and thermogenesis and reduces food intake [47], [48]. In the present study, energy metabolism was not measured and food intake was standardized. However, the distinct short-term increase in sympathetic outflow could have caused an increase in energy metabolism and energy expenditure. This is in line with a study by Addicott et al. (2007), which showed that moderate alcohol consumption increases physical activity during rising BACs. Surprisingly, when sympathetic tone was returned to normal values, parasympathetic tone stayed decreased and sympathovagal balance was not restored 2 hours after consumption as shown by elevated levels of the LF:HF ratio an heart rate. However, heart rate was recorded until 2.5 hour after consumption and at this time its levels were recovered. Whether this 2 hours increase in sympathovagal balance is related to long-term effects is not known. Epidemiological studies found inconsistent results on the association between chronic moderate alcohol consumption and heart rate variability [49]–[51]. An acute increase in sympathovagal balance is also seen during physical activity and mental stress [52]. Furthermore, previous studies showed that even a meal increased sympathovagal balance for 1 or 2 hours due to a continuous inhibition of parasympathetic activity [53], [54]. Therefore, we suggest that the acute increase in sympathovagal balance after combined food and alcohol consumption indicates an activated physiological state of the body, which may be generated to restore homeostasis.

ANS activity was found to be related to the arousal dimension of mood. The increased sympathetic tone was related to increased happiness and stimulation and decreased calmness and fatigue. This indicates that sympathetic activity was related to increased arousal and can be used as an objective measure of the arousal dimension of mood. The reduced parasympathetic tone was related to decreased mental alertness and increased sedation, suggesting this was caused by a central effect of alcohol. These associations are not in line with the idea that mood states, in contrast to emotions, are not related to ANS activity [25]. However, Matthews et al. (1990) also found a relation between skin conductance and self-report of the arousal dimension of mood in subjects measured prior to an intelligence test [55].

Strengths of the study are the randomized crossover design and the controlled study conditions. However, the study design also has some limitations that warrant consideration. We did not measure the mood effects of a meal separately from the alcohol effects. Therefore we cannot disentangle the alcohol and meal effects on mood. Second, although the alcohol intervention was blinded, the subjects might have noticed the alcohol content of the beverages. A confounding effect of expectancy in the observed mood effects can therefore not be excluded. Third, mood was measured in a laboratory setting and the physiological measurements may have influenced the mood of the subjects. However, we tried to interfere as little as possible with the physiological measurements. The subjects did not notice much of the autonomic nervous system measurements as they were carrying a small ambulatory device, which all electrodes from the ECG and skin conductance were connected to, so they could move freely in the rooms.

The study was carried out in women and the generalizability of the results to men may not be directly possible because men and women showed different mood effects of a meal in a previous study [56]. Furthermore, subjects were eating and drinking alone in this study. Social interaction has been shown to influence mood effects of alcohol and therefore the results may not be generalizable to food and alcohol consumption in a social setting [57], [58].

To conclude, mood inductions and autonomic nervous system parameters may be useful to evaluate mood changes by nutritional interventions. Moderate alcohol consumption elevates happiness in an unpleasant ambiance. However, drinking alcohol during a pleasant mood results in an equally positive mood state. Consuming alcohol with a meal does not result in different mood changes than alcohol consumption alone, although sedative feelings may have an earlier onset.

## Supporting Information

Checklist S1 **CONSORT checklist of information to include when reporting a randomised trial.**
(DOC)

Protocol S1 **Study protocol ‘Effect of moderate alcohol consumption on postprandial mood’.**
(PDF)
