# Supplementary material for: The Biphasic Effects of Moderate Alcohol Consumption with a Meal on Ambiance-Induced Mood and Autonomic Nervous System Balance: A Randomized Crossover Trial
Source: PLoS One. 2014 Jan 21;9(1):e86199. doi: 10.1371/journal.pone.0086199 (PMC3897660; doi:10.1371/journal.pone.0086199)
Supplement: Protocol S1 — Study protocol ‘Effect of moderate alcohol consumption on postprandial mood’. (PDF) [file pone.0086199.s002.pdf]

TNO
Utrechtseweg 48
P.O. Box 360
3700 AJ Zeist
The Netherlands

www.tno.nl
T +31 88 866 60 00
F +31 88 866 87 28

TNO Protocol

P9334 |

The effect of moderate alcohol consumption with a meal in different ambiances on postprandial mood, evaluated by subjective and physiological responses

Short title	Effect of moderate alcohol consumption on postprandial mood
Date	September 9th 2011
At the request of	Foundation for Alcohol Research (SAR)
Drafted by	Ilse Schrieks<br>Annette Stafleu<br>Henk Hendriks
TNO project number	051.01167
TNO study code	9334
Status	Revised Final
Previous versions	Final, August 23rd 2011
Number of pages	49
Number of appendices	13
Number of forms	9

All rights reserved.
No part of this publication may be reproduced and/or published by print, photoprint, microfilm or any other means without the previous written consent of TNO.

In case this report was drafted on instructions, the rights and obligations of contracting parties are subject to either the Standard Conditions for Research Instructions given to TNO, or the relevant agreement concluded between the contracting parties. Submitting the report for inspection to parties who have a direct interest is permitted.

© 2011 TNO

Contents

1 Responsible personnel and testing facilities 5
1.1 Sponsor(s) 5
1.2 Testing facilities and responsible personnel 5
1.3 Facility for the preparation of iv-test substance 6
1.4 Facility for the preparation and dispense of pharmaceutical products 7
1.5 Responsibilities 7

2 List of Abbreviations (alphabetical order) 8

3 Approval of the protocol 9
3.1 Sponsor 9
3.2 TNO 9

4 Summary 10

5 Introduction 12

6 Objectives of the study 16

7 Study design 17

8 Study substances and treatments 18
8.1 Study substances 18
8.1.1 Description 18
8.1.2 Safety of handling 18
8.1.3 Supply to TNO 18
8.1.4 Storage conditions 18
8.1.5 Chemical analyses 18
8.1.6 Remainder of study substances 18
8.2 Study treatments 18
8.2.1 Description 18
8.2.2 Standardized meal/diet with treatment study substances 19
8.2.3 Treatment code 19
8.2.4 Blinding and unblinding 19
8.2.5 Supply to subjects 20
8.2.6 Labelling 20
8.3 Accountability 20
8.4 Safety of treatment 20
8.5 Standardized diet 20
8.6 Restrictions 21

9 Subjects 22
9.1 Population base 22
9.2 Number of subjects and rationale for the number 22
9.3 Replacement and drop-outs 22
9.4 Inclusion criteria 22
9.5 Exclusion criteria 23

10 Informing the subjects 24

10.1 Aim of the study towards the participants ... 24
10.2 Information session and participation procedure ... 24

11 Study procedures and parameters ... 25
11.1 Pre-study screen, eligibility and selection ... 25
11.2 Pre-study screening parameters ... 25
11.2.1 Anamnesis ... 25
11.2.2 Physical examination ... 25
11.3 Familiarisation session ... 25
11.4 Allocation to entry number and study treatment ... 26
11.5 Assessments ... 26
11.6 In-study parameters ... 27
11.6.1 Subjective response questionnaires ... 27
11.6.2 Blood parameters ... 27
11.6.3 Non-invasive measures ... 28
11.6.4 Adverse events ... 28
11.6.5 Serious Adverse Events (SAEs) ... 28
11.6.6 Compliance with study instructions and treatment ... 29
11.6.7 In-study samples and analyses remark ... 29
11.7 Safety of subjects ... 29
11.8 Last assessment ... 29
11.9 Criteria for withdrawal or premature discontinuation ... 29
11.10 Payment of the subjects ... 29

12 Code and handling of samples ... 31
12.1 Sample coding ... 31
12.2 Sample collection, preparation, storage and transfer ... 31
12.2.1 Sample collection in-study ... 31
12.2.2 Registration of sample storage and transfer ... 31
12.3 Remainder samples and archive samples ... 32

13 Documentation ... 33

14 Data management and statistics ... 34
14.1 Data management ... 34
14.2 Missing values ... 34
14.3 Statistical analysis ... 35
14.3.1 Research questions / hypotheses ... 35
14.3.2 Descriptive parameters ... 35
14.3.3 Derived parameters ... 35
14.3.4 Analysis ... 35

15 Reporting ... 37

16 Public disclosure ... 38

17 Timing ... 39

18 Insurance of subjects ... 40

19 Ethics and quality ... 41

19.1 General 41
19.2 Good Clinical Practice 41
19.3 Quality assurance and monitoring 41

20 Retention of records, samples and specimens 42

21 References 43

22 List of Appendices and Forms 49

1 Responsible personnel and testing facilities

1.1 Sponsor(s)

Foundation for Alcohol Research (SAR)
Postbus 10413
2501 HK Den Haag

Responsible person
Mr C.J. Adema
Phone : +31 70 3180710
Fax : +31 70 3106173
Email : cjadema@dranken.org

1.2 Testing facilities and responsible personnel

TNO Zeist
Utrechtseweg 48
P.O. Box 360
3700 AJ ZEIST
Phone : +31 88 86 66 000
Fax : +31 88 86 68 728

Responsible persons at TNO:

Earth, Environmental and Life Sciences/*Pharmacokinetics & Human Studies*
Principal investigator
Mr H.F.J. Hendriks, PhD
Phone : +31 88 86 61 633
Fax : +31 88 86 68 754
Email : Henk.hendriks@tno.nl

Deputy principal investigator
Mrs W.J. Pasman, PhD
Phone : +31 88 86 65 129
Fax : +31 88 86 68 754
Email : Wilrike.pasman@tno.nl

Study assistant
Ms I.C. Schrieks, MSc
Phone : +31 88 86 64 228
Fax : +31 88 86 68 754
Email : Ilse.schrieks@tno.nl

Medical investigator
Mrs W.A.A. Klöpping-Ketelaars, MD, PhD
Phone : +31 88 86 65 980
Fax : +31 88 86 68 754
E-mail : Ineke.klopping@tno.nl

Datamanager
Mrs A.E. Dutman, MSc
Phone : +31 88 86 61 725
Fax : +31 88 86 68 754
E-mail : Ellen.dutman@tno.nl

Study nurse
Mrs H.J. Fick-Brinkhof, SRN
Phone : +31 88 86 61 596
Fax : +31 88 86 68 754
E-mail : Henriette.fick@tno.nl

Research manager Pharmacokinetics and Human studies
Mr J. de Groot, PhD
Phone : +31 88 86 62 399
Fax : +31 88 86 68 767
E-mail : Jeroen.degroot@tno.nl

Earth, Environmental and Life Sciences/*Quality and Safety*
Biostatistician
Mrs C.M. de Jong-Rubingh, PhD
Phone : +31 88 86 64 196
Fax : +31 30 69 44 894
E-mail : Carina.dejong@tno.nl

TNO Triskelion BV/*Toxicology & Applied Pharmacology*
Clinical Chemistry Laboratory
Mr V.J.H. Gielen
Phone : +31 88 86 61 751
Fax : +31 30 69 60 264
E-mail : Vincent.gielen@tno.triskelion.nl

Analytical Chemistry
Mrs M.M.H. van Lipzig, PhD
Phone : +31 88 86 61 654
Fax : +31 30 69 44 894
E-mail : Marola.vanlipzig@tno.nl

Mr F. van Schaik
Phone : +31 88 86 61 608
Fax : +31 30 69 44 894
E-mail : Frank.vanschaik@tno.triskelion.nl

Mr L. Coulier, PhD
Phone : +31 88 86 65 109
Fax : +31 30 69 22 894
E-mail : Leon.coulier@tno.nl

1.3 Facility for the preparation of iv-test substance

Not applicable

1.4 Facility for the preparation and dispense of pharmaceutical products

Not applicable

1.5 Responsibilities

The sponsor will be responsible for the financial compensation for the conduct of the study. Moreover the sponsor is responsible for the insurance according to the "WMO". Insurances for material damage and accidents during the travel to and from TNO and during the stay at TNO are TNO's responsibility.

Mr Hendriks will be responsible for the overall conduct of the study. Ms Schrieks assists in the daily conduct of the study. Mr Hendriks will also be responsible for monitoring dietary intake, receipt, coding, distribution, storage, accountability, and labelling of study substances. Most of these actions will be carried out by Mrs Fick-Brinkhof.

Mrs Klöpping-Ketelaars will be responsible for the selection of the subjects, medical aspects of the study, documentation, and interpretation and reporting of the AEs and SAEs.

Mrs Fick-Brinkhof will be responsible for the daily conduct of the clinical part of the study, dispensing of the study substances to the subjects and contacts with the subjects.

Mrs Dutman will be responsible for data management.

Mrs de Jong-Rubingh will be responsible for allocation of the subjects to entry-numbers and treatment.

Mr Gielen will be responsible for measurements of ethanol, insulin, cortisol, glucose and FFA levels.

Mrs van Lipzig will be responsible for measurements of CCK-8, ghrelin, GLP-1 and ACTH.

Mr Schaik will be responsible for measurements of Trp:LNAA ratio.

Mr Coulier will be responsible for measurements of endocannabinoids and N-acyl-serotonins.

Mr de Groot, Research manager Pharmacokinetics and Human studies, by signing the protocol, declares that the expertise, competence and experience of the local researchers, and the local facilities are appropriate for the adequate conduct of the study, which is consistent with the institution’s policies on research and human subjects**.**

2 List of Abbreviations (alphabetical order)

2-AG	: 2-Arachidonoylethanolamine
5-HT	: 5-Hydroxytryptamine
ACTH	: Adrenocorticotropic hormone
AE	: Adverse Event
AEA	: Anandamide
ANOVA	: Analysis of Variance
AUC	: Area under the curve
BAES	: Biphasic Alcohol Effects Scale
B-BAES	: Brief BAES
BAC	: Blood alcohol concentration
BMI	: Body Mass Index
CCK	: Cholecystokinin
CCMO	: Central Committee on Research involving Human Subjects
CRO	: Contract Research Organisation
DEBQ	: Dutch Eating Behaviour Questionnaire
DMQ-R	: Drinking Motives Questionnaire - Revised
EDTA	: Ethylene Diamine Tetra Acetic acid
FFA	: Free fatty acids
GCP	: Good Clinical Practice
GLP-1	: Glucose-like protein-1
HPA	: Hypothalamic-pituitary-adrenal
HRV	: Heart rate variability
ICD-10	: International Statistical Classification of Diseases and Related Health Problems
ICH	: International Conference on Harmonisation of Technical Requirements for Registration of Pharmaceuticals for Human Use
IRB	: Institutional Review Board
LNAA	: Large Neutral Amino Acids
MEC	: Medical Ethics Committee (Medisch Ethische ToetsingsCommissie)
OECD	: Organisation for Economic Cooperation and Development
POMS	: Profile of Mood States
PPW	: Postprandial Wellness questionnaire
QAU	: Quality Assurance Unit
SAE	: Serious Adverse Event
SAR	: Foundation for Alcohol Research
SCL	: Skin conductance level
SOP	: Standard Operating Procedure
STAI	: State-Trait Anxiety Inventory
TNO	: Nederlandse organisatie voor Toegepast Natuurwetenschappelijk onderzoek (Netherlands Organisation for Applied Scientific Research)
Trp	: Tryptophan
VTC	: Visit Time Code
WHO	: World Health Organisation
WMA	: World Medical Association
WMO	: Wet Medisch Onderzoek met mensen (Medical Research involving Human Subjects Act)

Definitions of terms

‘Participants’ or ‘candidates’ refer to the persons who are interested in participating in the study. ‘Subjects and volunteers’ refer to the persons who actually participated in the study. ‘Patients’ is not applicable in this study, for this study is performed with apparently healthy subjects.

3 Approval of the protocol

3.1 Sponsor

C.J. Adema<br>Foundation for Alcohol Research	Signature	09-09-2011<br>Date (dd-mm-yy)

3.2 TNO

H.F.J. Hendriks<br>Principal investigator	Signature	09-09-2011<br>Date (dd-mm-yy)
W.A.A. Klöpping – Ketelaars<br>Medical investigator	Signature	12-09-2011<br>Date (dd-mm-yy)
C.M. de Jong - Rubingh<br>Statistician	Signature	12-09-2011<br>Date (dd-mm-yy)
J. de Groot<br>Research Manager<br>Pharmacokinetics and Human Studies	Signature	12-09-2011<br>Date (dd-mm-yy)

4 Summary

Rationale: Food choice is influenced by postprandial mood; the feelings of well-being after a meal. Postprandial mood can be measured by subjective responses. Physiological responses may play an important role in the generation of postprandial mood. However, the relationship between subjective and physiological responses after a meal is not clear yet. To investigate this relationship, moderate alcohol consumption will be used as a mood modulator, because of its well-studied effects on mood. Postprandial mood depends on the current mood state. Therefore we will manipulate the current mood state by changing the ambiance to measure the influence of moderate alcohol consumption with a meal on postprandial mood in a pleasant or unpleasant ambiance.

Primary objective
To determine whether moderate alcohol consumption with a meal in different ambiances affects postprandial mood, evaluated by subjective (POMS, B-BAES, PPW questionnaires) and physiological (ACTH, cortisol, TRP:LNAA ratio, ghrelin blood concentration) parameters.
Secondary objective
To investigate whether moderate alcohol consumption with a meal in different ambiances affects other physiological parameters related to mood, which are not part of the primary objective (endocannabinoids, N-acyl serotonins, β-endorphin, dopamine, insulin, CCK, GLP-1, FFA, glucose, heart rate, HRV and SCL).

Study design: Randomized, placebo-controlled, single-blind, cross-over trial

Blinding: Subjects will not be informed about the study objective, but about a study aim that is slightly different. The alcohol content of the drinks will be blinded to the subjects, they will be informed that alcohol content may vary each treatment day.

Study population: 28 apparently healthy, normal weight (BMI 18.5-27 kg/m^2) women (18-45 y), taking oral contraceptives.

Intervention: 4 times having dinner at TNO Zeist with either 3 glasses of sparkling white wine (~30g alcohol) or alcohol-free sparkling white wine in either a pleasant or unpleasant meal ambiance.

Outcome parameters:
Subjective response: Effect of moderate alcohol consumption with a meal in different ambiances on subjective scales: POMS (extended with mood scales happiness and calmness), Brief BAES (B-BAES) and the Postprandial Wellness (PPW) questionnaire.
Physiological response: Effect of moderate alcohol consumption with a meal in different ambiances on the physiological response of hormones of the HPA axis, Trp:LNAA ratio and ghrelin.
Other physiological parameters: The effect of moderate alcohol consumption with a meal in different ambiances on physiological parameters related to mood, which are not part of the primary objective (endocannabinoids, N-acyl serotonins, β-endorphin, dopamine, insulin, CCK, GLP-1, FFA, glucose, heart rate, HRV and SCL).

Nature and extent of the burden and risks associated with participation, benefit and group relatedness: Subjects need to visit the study site five times, once for a screening and four times for a treatment day. During these visits blood will be collected five times. The total amount collected during the whole study will be less than 460 mL

blood. The study will be performed in women, because women are suspected to have a different postprandial mood response than men (1, 2), which might be due to female hormones (3). We will only include women taking oral contraceptives, because they have a reduced variation in female hormones over the menstrual cycle. A large number of women use oral contraceptives nowadays, therefore this group will reflect a large population of women. Women above 45 years will be excluded, because we will include only premenopausal women.

5 Introduction

Developing a method to measure and model postprandial mood
Food consumption influences our mood, the short-term general feelings of well-being. Previous studies showed that postprandial mood is an important factor in food choice (4, 5) and there has been considerable interest in the effects of meals and beverages on postprandial mood. Postprandial mood can be defined as the general feelings of well-being after consumption and can be measured by self-reports of subjective feelings in questionnaires. Physiological responses to food intake, such as changes in hormone and neurotransmitter concentrations in the blood, play an important role in the generation of postprandial mood (6). However, the relationship between physiological and subjective responses of postprandial mood is not clear yet.
Therefore, the ultimate objective of this study is to develop a method to evaluate postprandial mood and to develop a multi-factorial model describing the associations between physiological postprandial responses and subjective feelings of postprandial mood.
The effect of a food product on postprandial mood, depends also on the current mood state and can either improve, worsen, reinforce or attenuate it. Meal settings differing in environmental factors (e.g. lightening, music, decoration) are known to affect food intake and eating behaviour (7-9). It is hypothesized that mood plays an important role in these effects (7). Consequently, meal settings differing in ambiance pleasantness are hypothesized to influence the mood state during and after the meal.

Alcohol as mood modulator
To investigate the association between subjective and physiological responses of postprandial mood, moderate alcohol consumption (3 glasses of sparkling white wine; 30g alcohol) will be used as a mood modulator. Extensive research has been done on the effects of moderate alcohol consumption on mood. During rising BACs, alcohol has a stimulating effect, reported by feelings of euphoria, talkativeness and elation. During falling BACs, tension-reducing effects and feelings of fatigue are examples of reported sedative effects (10-13).
The effect of alcohol on mood is suggested to be reinforcing, with greater positive or stimulating effects in a neutral or positive pre-drinking mood and greater sedating effects in a depressive pre-drinking mood (12). Though, the tension-reducing effect of alcohol is an improvement of a negative pre-drinking mood.
Non-pharmacological factors (e.g. expectancy and social context) are determining a large part of the alcohol-induced mood change. The expectancy effect is predicted to be as important as the pharmacological effect of alcohol (14). However, Pliner and Cappell (1974) determined the effect of alcohol on mood consumed in different social contexts (drinking solitary or in a group) including a placebo-effect in their model, and concluded that mood changes were even more influenced by social context than by expectancy. They found larger mood changes in individuals drinking in a group compared to drinking alone, and also the type of mood changes was different (15). These findings emphasize that non-pharmacological factors as expectancy and social context are important to take into account when measuring alcohol-induced mood changes.
Although alcohol consumption with dinner is very common in Western countries, only a few studies investigated the influence of alcohol consumption with a meal on postprandial mood (16, 17). The combination of food and alcohol consumption may result in a different mood change compared to single alcohol consumption, because food may both directly and indirectly, by influencing alcohol absorption, affect the alcohol-induced mood change (18-20).

Therefore, the aim of this study is to determine the effects of moderate alcohol consumption with a meal in different ambiances on postprandial mood, evaluated by subjective and physiological responses. Thus, the current mood state will be manipulated by environmental factors to induce a pleasant or unpleasant meal ambiance and mood state. Thereby, we will be able to measure the influence of moderate alcohol consumption with a meal on postprandial mood in a positive or negative ambiance.

Subjective response

There are two validated and reliable questionnaires mainly used in previous research to assess the subjective response to moderate alcohol consumption; the Profile of Mood States (POMS) and the Biphasic Alcohol Effects Scale (BAES) (21-23). The POMS is a widely used questionnaire measuring subjective feelings of mood and has been often used in research interventions to investigate the influence of a treatment on mood. It was originally developed to measure mood in a clinical setting, therefore it consists of four negative mood scales (depression, fatigue, tension, anger) and just one positive mood scale (vigour). To make the POMS questionnaire more balanced, two positive mood scales from the 32-item Brunel Mood Scale are added to measure happiness and calmness (24). The BAES is a questionnaire developed specifically to measure the biphasic effects of alcohol consumption; the stimulating effects during ascending blood alcohol levels and sedative effects during descending blood alcohol levels (21, 23).
Previous research showed mood effects of single alcohol consumption measured by the POMS, the BAES or self-developed visual analogue scales (15, 25-31). However, there is few data on the effects of alcohol consumption with a meal and these data is inconsistent (16, 17). Therefore, in this study we would like to investigate the effect of moderate alcohol consumption with a meal on the postprandial subjective response.
During the postprandial state, also feelings of satiety may influence mood, but no questions on satiety are included in mood questionnaires as the POMS and BAES. However, Boelsma et al. (2010), developed a questionnaire to evaluate ‘postprandial wellness’ (PPW) including items on satiety, mood and gastric comfort (32) (see TNO report P5820). This questionnaire will be used to measure satiety feelings related to mood.

Physiological response

Many physiological parameters are suggested to be important factors in mood generation. For this study we will focus on physiological parameters that have been studied before in alcohol interventions in humans, because many parameters are only measurable in the brain or are very difficult to detect in peripheral blood. Therefore, the primary parameters of the physiological response are the hormones of the hypothalamic-pituitary-adrenal (HPA) axis, the Trp:LNAA ratio (reflecting serotonin response) and the ghrelin concentration in the blood.

Stress hormones

The HPA axis is a neuroendocrine system that is stimulated during stress, resulting in a fast increase in the hormones adrenocorticotropic hormone (ACTH) and cortisol in the blood (33). Previous data indicate that acute alcohol ingestion of 0.695 g/kg compared to placebo may also stimulate the HPA axis, resulting in increased ACTH and cortisol concentrations, with peak levels 10-20 min and 30 min after drinking initiation, respectively (30). Other studies failed to demonstrate stimulating effects on the HPA axis (34-36). Though, Spaak et al. (2008) found a trend for a higher cortisol response after red wine or ethanol intake compared to water, and a higher ACTH response after red wine intake compared to ethanol and water (37). In addition, King et al. showed increased cortisol levels after alcohol intake during descending BACs (38).

Serotonin system
The effect of alcohol on mood may be mediated by different neurotransmitter systems. The serotonin system is known to be involved in the regulation of mood and cognitive performance and previous research indicates the role of the 5-hydroxytryptamine (5-HT) receptor subtype in the effect of moderate alcohol consumption on mood (39). Brain neurotransmitter 5-HT receptor activity is shown to be influenced by the availability of the plasma precursor tryptophan (Trp) in relation to other large neutral amino acids (LNAA). The plasma Trp:LNAA ratio can be influenced by food intake. For example, a carbohydrate-rich, low-protein diet increases the ratio, resulting in higher brain 5-HT levels (40). In a study by Markus et al. (2004), it was shown that in male volunteers moderate alcohol consumption decreases the Trp:LNAA ratio after 2h (16).

Endocannabinoids
Endocannabinoids, which are conjugates of fatty acids with ethanolamides, are thought to play a role in the influence of alcohol on mood and reward. Roberto et al. (2010) showed that endocannabinoids may have a modulatory role in the effect of alcohol on the neural activity in the central amygdala (41). The acute effects of alcohol on the endocannabinoid system have been shown in cultured cells and animals, but not yet in humans (42). The results in cultured cells and animals are inconsistent. Alcohol consumption was shown to suppress the concentration of brain endocannabinoids anandamide (AEA) and 2-arachidonoylethanolamine (2-AG) (36, 43-45). However, self-administration of alcohol in rats resulted in enhanced interstitial levels of 2-AG and not AEA, measured by *in vivo* microdialysis, with peak 2-AG levels simultaneous to peak BAC (46).
There is growing interest in a recently discovered group of fatty acid derived mediators, which are conjugates of fatty acids with serotonin, or N-acyl serotonins (47). Their function is still unknown, but they might be involved in mood. N-acyl serotonins have been shown to inhibit the activity of the enzyme fatty acid amide hydrolase *in vitro*, responsible for degradation of endocannabinoids (47). Therefore, in this study we are interested in the influence of alcohol on both endocannabinoids and N-acyl-serotonins and in their relation to subjective mood.

B-endorphin
It has been suggested that β-endorphin is involved in the rewarding and reinforcing effects of alcohol. *In vitro* and animal studies showed that alcohol stimulates the release of β-endorphin in the brain (48). Additionally, Frias et al. (2002) showed that alcohol intoxication results in higher plasma β-endorphin levels in men and women (49). However, in a study of Dai et al. (2005) β-endorphin levels were attenuated after moderate alcohol consumption (50).

Dopamine
Another neurotransmitter involved in mood and reward is dopamine. High dopamine levels in the brain are associated with positive mood and drugs acting on the dopamine receptor are also influencing the mood state (51). In a study by Boyer et al. (2004) increased dopamine levels in the blood were found after champagne consumption, but not after still white wine consumption.

Satiety hormones
Satiety hormones, such as ghrelin, insulin, cholecystokinin (CCK) and glucose-like protein-1 (GLP-1), may also influence postprandial mood.
Ghrelin is an appetite-regulating hormone that has also been associated with mood, stress and anxiety. Plasma ghrelin levels were found to be increased during a stress reaction and ghrelin seems to be involved in the reward of food and alcohol (52). Food consumption results in a decline of plasma ghrelin levels, with higher calorie content

resulting in lower circulating ghrelin (53). Moderate alcohol consumption was found to attenuate ghrelin levels even more than could be expected from the caloric value, and the ghrelin levels did not return to baseline within 5h, while they returned to baseline about 3h after food consumption (36, 53-55). However, no studies have been performed on the effect of moderate alcohol consumption with a meal on ghrelin levels.
After food consumption individuals may experience sleepiness. It has been suggested that CCK production, caused by presence of food (mainly lipids) in the intestine is partly responsible for the induction of sleepiness. Insulin might also be a mediating factor, however the role of insulin is less clear (1). CCK infusion in males caused sleepiness and fatigue (56, 57). In this study we will measure the effect of moderate alcohol consumption on satiety hormones, because of their potential effect on mood.

Metabolites
There is some evidence that metabolites like glucose and free fatty acids (FFA) may influence mood. Calissendorf et al. (2006) showed that low glucose levels are associated with irritability and more aggressive behaviour in frustrating situations (58).
Endocannabinoids are derived from FFAs. In a previous study by Joosten et al. (2010) (see TNO report P7573), elevated endocannabinoid levels are found to be simultaneously increased with FFA levels (42). Therefore it is hypothesized that FFAs may indirectly, as precursors of endocannabinoids, have an effect on postprandial mood.

Non-invasive measures
Heart rate, heart rate variability (HRV) and skin conductance level (SCL) are indices of alcohol’s effect on emotional arousal. The activity of the sympathetic and parasympathetic nervous system can be estimated by heart rate variability and skin conductance level (59, 60). Previous studies showed an increased heart rate and skin conductance level after alcohol consumption (28, 37, 61). Heart rate variability was found to be decreased after moderate alcohol consumption (28, 59, 62). Though, Stritzke et al. (1995) found an attenuated skin conductance level after alcohol consumption (63).
In this study, heart rate, heart rate variability and skin conductance level will be measured during baseline, ascending and descending BACs to investigate the amount of arousal during these different phases, and to determine changes in the parasympathetic and sympathetic tone.

Blood alcohol concentration
Blood alcohol concentration (BAC) will be measured in this study on the same time points as the other parameters, to be able to explain and relate the subjective and physiological responses.

6 Objectives of the study

Primary objective
To determine whether moderate alcohol consumption with a meal in different ambiances affects postprandial mood, evaluated by subjective and physiological parameters.
Secondary objective
To investigate whether moderate alcohol consumption with a meal in different ambiances affects other physiological parameters related to mood, which are not part of the primary objective.

Primary hypothesis
It is hypothesized that moderate alcohol consumption with a meal in different ambiances changes the response of subjective and physiological parameters of mood.
Secondary hypothesis
It is hypothesized that moderate alcohol consumption with a meal in different ambiances changes the response of other physiological parameters related to mood, which are not part of the primary objective.

Primary outcome parameters
Subjective response:
Effect of moderate alcohol consumption with a meal in different ambiances on subjective scales:
- POMS (extended with mood scales happiness and calmness)
- Brief BAES
- Postprandial wellness (PPW) questionnaire

Physiological response:
Effect of moderate alcohol consumption with a meal in different ambiances on:
- Stress hormones (ACTH and cortisol)
- Serotonin system (Trp:LNAA ratio)
- Satiety hormone (ghrelin)

Secondary outcome parameters
Physiological response:
- Endocannabinoids and N-acyl serotonins
- β-endorphin
- dopamine
- Satiety hormones (Insulin, CCK and GLP-1).
- Metabolites (FFA and glucose)
- Non-invasive measures (Heart rate, HRV and SCL)

7 Study design

The study is designed as a randomized, placebo-controlled, single-blind, cross-over study. The study has a 2x2 factorial design, with treatment factors 'alcohol' (yes/no) and 'ambiance' (pleasant/unpleasant). Therefore, the subjects will visit TNO Zeist on four treatment days separated by at least 3 days, which may extend to 7 days (which is preferred).
A cross-over design will be used, because large between-subject variations are expected in the subjective and physiologic responses of the primary outcomes.
Subjects will be divided in 4 groups with different treatment order according to the latin square design.

Treatment order (Latin square)

1	Unpleasant ambiance + alcohol	Pleasant ambiance + alcohol	Unpleasant ambiance - alcohol	Pleasant ambiance - alcohol
2	Unpleasant ambiance - alcohol	Unpleasant ambiance + alcohol	Pleasant ambiance - alcohol	Pleasant ambiance + alcohol
3	Pleasant ambiance + alcohol	Pleasant ambiance - alcohol	Unpleasant ambiance + alcohol	Unpleasant ambiance - alcohol
4	Pleasant ambiance - alcohol	Unpleasant ambiance - alcohol	Pleasant ambiance + alcohol	Unpleasant ambiance + alcohol

8 Study substances and treatments

8.1 Study substances

8.1.1 Description

Test substance : sparkling white wine (11% alcohol) (P9334 B09)
Reference substance : alcohol-free sparkling white wine (<0.5% alcohol) (P9334 B10)
The test substance is a prosecco vino frizzante. The reference substance is a sparkling de-alcoholized wine. Both the sparkling white wine and de-alcoholized wine are commercially available. The de-alcoholizing process is performed by a de-alcoholizing company. During this process the wine is heated till 28 °C and applied vacuum.
The study substances are selected based on a pilot study with 7 participants who scored different sparkling white wines with and without alcohol on palatability and wine taste. These wines were selected because their scores on both scales were comparable and above average (palatability: 59 for the test substance and 58 for the reference substance; wine taste: 67 for the test substance and 52 for the reference substance, with a scoring range 0-100).

8.1.2 Safety of handling

As the test substance and reference substance are both commercially available food products, no special precautions have to be taken in handling of the substances.

8.1.3 Supply to TNO

Sparkling white wine and alcohol-free sparkling white wine are both commercially available. All beverages will be purchased from one expiry date and one batch. Beverages will be supplied by a TNO employee. Products will be from single batches to limit variability between the substances.

8.1.4 Storage conditions

White wine and alcohol-free white wine will be stored at room temperature in a dark place. At the test days, beverages will be served cold (~5 °C) at TNO Zeist.

8.1.5 Chemical analyses

A few bottles of wine and alcohol-free wine out of the total batch will be stored for analysis in order to be able to perform analyses or to check alcohol percentage.

8.1.6 Remainder of study substances

Most of the remaining study substances will be discarded at the end of the clinical part of the study. Taking in account the possible need for additional analyses, part of the remaining study substances will be stored until the final report is accepted by the sponsor (if the nature of the substance allows this).

8.2 Study treatments

8.2.1 Description

There are two treatment factors in this study:

Treatment factor 1, alcohol, consists of consumption of either:
Treatment A (2x): 340 mL of sparkling white wine (3 glasses = 30 g alcohol)

Treatment B (2x): 340 mL of alcohol-free sparkling white wine (3 glasses < 2g alcohol)
Beverages will be used with a dinner at TNO Zeist.

Treatment factor 2, ambiance, consists of either:
Treatment 1 (2x): pleasant meal ambiance
Treatment 2 (2x): unpleasant meal ambiance

The meal ambiance will be manipulated by environmental factors and will be enhanced; the subjects have to watch a movie scene on a computer which is either sad or happy in the room before they start eating. The movie scenes will be from two different animation movies and will not contain content potentially shocking for some viewers.

	**Pleasant ambiance**	**Unpleasant ambiance**
Light	Slightly dimmed light	Very bright light
Music	Cheerful music	No music
Cleanness	Clean space	Dirty dustbin
Decoration	Decorated: decorated walls, table-cloth, plants, nice plate	Undecorated: white walls, no table-cloth, no plants, plastic plate
Movie scene	Happy scene (ca. 3min) of 2 animation movies (randomized)	Sad scene (ca. 3 min) of 2 animation movies (randomized).

Subjects will stay in separate rooms when they are having the intervention at TNO, to avoid social interaction with the other subjects.

8.2.2 Standardized meal/diet with treatment study substances

Subjects will consume a standardized meal as dinner on the treatment days. This meal will be comparable to a Dutch evening meal. Subjects have to consume the total meal and all the drinks.

8.2.3 Treatment code

The coding of the 2 treatment factors is as follows:
<u>Treatment factor alcohol:</u>
Treatment ‘A’ = sparkling white wine
Treatment ‘B’ = alcohol-free sparkling white wine

<u>Treatment factor ambiance:</u>
Treatment ‘1’ = pleasant meal ambiance
Treatment ‘2’ = unpleasant meal ambiance

8.2.4 Blinding and unblinding

The treatment factor ‘alcohol’ will be blinded for the subjects. They will be informed that the alcohol percentage in the wines may be different per treatment day. Furthermore, the beverage will be served in a closed cup and the serving temperature will be colder than advised (~5°C instead of ~8°C). We will check at the end of the last treatment day whether the subjects noticed the difference in alcoholic and nonalcoholic wines, by asking them the alcoholic percentage of wines of each study day (P9334 F09).
The treatment factor ‘ambiance’ cannot be blinded, because the subjects will notice the environmental differences between the pleasant and unpleasant ambiances, but they will not be informed about it.

Before the start of the clinical part of the study, the statistician will inform the study nurse in writing about the allocation of pre-entry number to entry number.

8.2.5 Supply to subjects

The meals and alcoholic drinks will be supplied to the subjects during the four treatment days, which they are obliged to finish totally. They have to consume first one glass of sparkling white wine within 5 min and afterwards they have to consume the meal and second and third glass of sparkling white wine within 15 min. So, they have 20 min in total to finish the alcoholic drinks and meal. Every subject is allocated randomly to one of the four treatment groups with a specific treatment order.

8.2.6 Labelling

Study substances will be labelled according to a study specific instruction as described in DHDV/ALG/711.
Each bottle/box will be labelled by a pre-printed label, as follows:
The TNO study number (9334) followed by a slash,
followed by the 2-digit entry number, followed by a slash,
followed by "treatment A" or "treatment B".

8.3 Accountability

Not applicable

8.4 Safety of treatment

The **test substances** (sparkling white wine and alcohol-free sparkling white wine) used in this study are commercially available and will be produced in accordance with safety and quality regulations of the commercial manufacturer. Therefore, test substances used are safe.
With an alcohol consumption of 30g (340 mL wine) the following BACs are expected for women with a body weight of 57kg – 80kg: 0.38‰ – 0.53‰. This has been calculated with the following formula (for alcoholic drinks with dinner in women):

$$\text{BAC (‰)} = \frac{\text{alcohol content (g) / (body weight (kg) * 0.5)}}{2}$$

Previous research showed that simultaneous food and alcohol consumption results in attenuated BACs and that consumption of 4 alcoholic drinks (40g) with a dinner meal in men resulted in BACs not higher than 0.5‰ and (64-66).

At the end of each treatment day, the subjects BAC is measured by a breathing analyzer, and is allowed to leave TNO when her BAC has dropped below 0.2‰. This will be measured in duplo with 10 min in between.

8.5 Standardized diet

The standardized diet will consists of a macaroni dish, served by a caterer. The nutrition composition is summarized in the table on the next page.

**Nutritional value**	**Per 100 g**	**Per meal (430g)**
Energy (kJ)<br>Energy (kcal)	466<br>111	2002<br>478
Protein (g)	5	23
Carbohydrates (g)<br>- sugar (g)	12<br>3	50<br>11
Fat (g)<br>- saturated fat (g)<br>- unsaturated fat (g)	4<br>1<br>3	19<br>5<br>14

8.6 Restrictions

Subjects will be asked to maintain their habitual diet, alcohol intake and activity pattern throughout the study. Subjects will have to refrain from alcohol for 24h prior to testing and caffeine on the afternoon of testing. Subjects will be asked to eat the same breakfast and lunch and are not allowed to exercise intensively on the 4 treatment days. Additionally, they have to refrain from eating 2h before testing.

9 Subjects

9.1 Population base

The subjects in this study will be recruited from the pool of volunteers of TNO, location Zeist and, if needed, a recruitment office may be contacted.

9.2 Number of subjects and rationale for the number

Twenty eight women will participate in the study. The rationale for the number of subjects is based on the primary objective to determine the ‘subjective response’.

<u>Subjective response:</u>
Several scales will be used to gain knowledge on the subjective response, but the POMS is the most widely used questionnaire and also shown to be valid and reliable for measurements of mood (67). Therefore, the rationale for the number of subjects is based on the POMS.
In a previous study by King et al. (1997), significant differences were picked up on the scales confusion, fatigue and vigour of the POMS. In this study 2 subgroups of 14 subjects were used, although with a slightly higher amount of alcohol, and with male subjects (25). In a study of Pasman et al. (2003), they picked up a significant difference on the scale fatigue of the POMS after consumption of two different breakfasts with 26 subjects (68). We expect to find a larger difference on the POMS scales between no alcohol or alcohol consumption with dinner, than between two breakfasts differing in macronutrient composition. Therefore, we did a power calculation based on this study. In this study a significant difference of 1.2 between the two breakfasts was shown. To reach a power of 0.8, with a significance level of 0.05 (2-sided), a difference of 1.2 (standard deviation of the difference between two values for a subject = 2.0) can be detected with 24 subjects.
To guarantee sufficient subjects more subjects will be included in case of possible drop-out.

9.3 Replacement and drop-outs

A maximum of 5 subjects will be reserve until Day 01 of the study. Subjects not completing the study for any reason will be considered as drop-outs.

9.4 Inclusion criteria

1. Healthy as assessed by the health and lifestyle questionnaire, (P9334 F02; in Dutch).
2. Females aged 18-45 years at Day 01 of the study*
3. Taking a monophasic combined oral contraceptive pill at Day 01 of the study, with 21 days of taking pills with active ingredients followed by 7 days taking no pills or continuous intake of the oral contraceptive pill**.
4. Body Mass Index (BMI) of 18.5-27 kg/m^2 *
5. Body weight between 57 and 80 kg
6. Normal Dutch eating habits as assessed by P9334 F02
7. Alcohol consumption ≥ 3 and ≤ 21 standard glasses/week*
8. Voluntary participation
9. Having given written informed consent

10. Willing to comply with the study procedures, including refrain from alcohol 24 h before the test days and refrain from caffeine during the afternoon of the test day.
11. Appropriate veins for blood sampling/cannula insertion according to TNO
12. Willing to accept use of all nameless data, including publication, and the confidential use and storage of all data for at least 15 years
13. Willing to accept the disclosure of the financial benefit of participation in the study to the authorities concerned.

* Volunteers with an age of 25-45 years, a BMI of 20-25 and alcohol consumption between 3-14 standard glasses/week are preferred for inclusion.
** With the recruitment letter, a list with accepted brands of oral contraceptive pills for participation will be added.

9.5 Exclusion criteria

Subjects with one or more of the following characteristics will be excluded from participation:

1. Participation in any clinical trial including blood sampling and/or administration of substances up to 90 days before Day 01 of this study.
2. Having a history of medical or surgical events or disease that may significantly affect the study outcome, particularly physiological disorders, or psychiatric, metabolic or endocrine disease and gastrointestinal disorders.
3. Use of medication that may affect the outcome of the study parameters (e.g. antidepressant drugs).
4. Having a family history of alcoholism
5. Having a history of alcohol or drug related problems
6. Smoking
7. Reported unexplained weight loss or gain of > 2 kg in the month prior to the pre-study screening
8. Reported slimming or medically prescribed diet
9. Reported vegan, vegetarian or macrobiotic
10. Recent blood donation (<1 month prior to the start of the study)
11. Not willing to give up blood donation during the study.
12. Pregnant (to their own knowledge) or lactating or wishing to become pregnant in the period of the study
13. Personnel of TNO Zeist, their partner and their first and second degree relatives
14. Not having a general practitioner
15. Not willing to accept information-transfer concerning participation in the study, or information regarding her health, like laboratory results, findings at anamnesis or physical examination and eventual adverse events to and from his general practitioner.

10 Informing the subjects

10.1 Aim of the study towards the participants

The aim of the study is to investigate the effect of moderate alcohol consumption with a meal on postprandial mood. However, we keep the subjects ignorant to the aim to avoid that subject’s expectations of postprandial mood effects will interfere with the pharmacologically caused postprandial mood effects. Keeping subjects ignorant is common in research with implicit measurements since the outcome of the study is easily influenced. The information subjects receive concerning the aim of the study is that we want to investigate the effect of different meal settings and alcohol on hormones and satiety.
The subjects will be kept blinded for the treatment factor ‘alcohol’; they will be informed that alcohol content may vary per treatment day. A blinding questionnaire at the end of the last treatment day will be used to check whether the blinding of the alcohol content was successful.

10.2 Information session and participation procedure

Candidates having informed TNO of being interested in possible study participation will be invited to come to TNO for an oral information session on the study.

The candidates will be informed verbally on the aim, the study procedures, the constraints and insurance of the study and receive a copy of the "written information for subjects" (P9334 F01).

After oral information a booklet will be handed out containing the following forms:
1. Subject checklist (pre-study) (P9334 F04)
2. Form P9334 F01 (written informed consent; in duplicate, in Dutch)
3. Form P9334 F02 (health and lifestyle questionnaire, in Dutch)
5. Form: Eligibility checklist
6. Form: Drinking Motives Questionnaire-Revised (DMQ-R) form (P9334 F06)
7. Form: State-Trait Anxiety Inventory (STAI) form (P9334 F07)
8. Form: Dutch Eating Behaviour Questionnaire (DEBQ) form (P9334 F08)
9. Form: End of trial

Volunteers willing to participate will be asked to sign both informed consent forms (one for themselves and one for TNO). Thereafter subjects will be asked to complete the health and lifestyle questionnaire. The volunteers participate on a voluntary base and can withdraw at any moment of the study.

On the basis of the oral and written information, volunteers will be given two to three days to consider participation.

Each subject will be allocated to a pre-entry number consisting of the TNO study code (9334), followed by a slash ('/'), followed by a 3-digit number starting at 101.

The medical investigator will inform in writing the general practitioner of each volunteer who has signed the informed consent form on his/her application for study participation.

11 Study procedures and parameters

No examinations will be carried out before the volunteer concerned gave her written informed consent.
For both the pre-study period and the treatment period checklists per subject will be used.

11.1 Pre-study screen, eligibility and selection

The volunteers will have a pre-study screening before the start of the treatment period. The pre-study screening will involve:
Measurement of vital signs, venous access, height, weight and body fat percentage.
The subjects also have to fill out a health and lifestyle questionnaire, the DMQ-R, STAI and DEBQ form (P9334 F02, P9334 F06, P9334 F07 and P9334 F08, respectively).

Based on the results of the pre-study screening, the medical investigator will establish the eligibility.
If a volunteer is eligible the principal investigator will inform her in writing and whether she will be invited to participate in the study or is reserve or whether she is not selected. If a volunteer is not eligible the medical investigator will inform her in writing.

11.2 Pre-study screening parameters

11.2.1 Anamnesis
There will be no interview (anamnesis) with the medical investigator.

11.2.2 Physical examination
No physical examination will be carried out.

Systolic and diastolic blood pressure and heart rate will be measured according to SOP SBJ/102, as described for screening parameters. In addition venous access will be checked.

Height will be measured according to SOP SBJ/104 (i.e. without shoes).

Whole body electrical resistance measurements will be performed by bio-impedance (according to the manual of the InBody 720). Subjects need to wear indoor clothing. Based on the data of the bio-impedance measurement, body fat percentage is measured. Body weight is also measured by the bio-impedance device (InBody 720). Data of other outcome measures of the Inbody will be not be used.

11.3 Familiarisation session

The volunteers will have a familiarisation session during the screening day. They will practice with the questionnaires to get familiar with them and the equipment for the skin conductance and heart rate variability measurements will be shown. After the screening day a macaroni dish similar to the dish served during the study will be provided to the volunteers, which they can try at home. This will reduce the possible stress present on the first treatment day.

11.4 Allocation to entry number and study treatment

After inclusion of all subjects, the statistician will randomly allocate the subjects to an entry number and a treatment group.
Entry numbers will consist of the TNO study number (9334), followed by a slash ('/'), followed by a 2-digit number (01-28).
The statistician will inform in writing the principal investigator or study assistant who will inform the study nurse on allocation to entry number and study treatment (see also § 8.2.3).

11.5 Assessments

The assessments to be done are summarized in the scheme below.

		Session:			
	Pre Study*	1**	2	3	4
Visit number***→		101	201	301	401
**Activity**					
Vital signs	+				
Body weight	+				
Height	+				
Body fat percentage	+				
DMQ-R form	+				
STAI form	+				
DEBQ form	+				
Familiarisation session	+				
Repeated blood sampling		+	+	+	+
Subjective response		+	+	+	+
Dispensing study substances		+	+	+	+
Well-being questionnaire		+	+	+	+
Blinding questionnaire					+
End of trial					+

* Pre Study is defined as the information/screening day and is not part of the clinical part of the study. Also the familiarisation session takes place during this day.
**Session 1 is defined as the first study day of the clinical part of the study.
***The visit time codes (VTCs) are based on the session number instead of study day number, because every subject will have 4 study days planned on different days. This is because subjects are only tested during the three contraceptive taking weeks, and not during the week taking no pills or hormone-free pills. The subjects can also choose to continue taking the contraceptive pill during the hormone-free week.

The first day of treatment will be specified as Session 1. There will be a wash-out period of at least 3 days, which may extend to 7 days (which is preferred).

In-study subjects visit TNO in total 4 days (**session 1, 2, 3, and 4**) for receipt of study substances and repeated blood collection. During these test days, subjects have to refrain from caffeine during the afternoon and have to use similar breakfasts and lunches on similar time points. They have to refrain from eating 2h before visiting TNO.
Subjects will come to premise at ca. 16:00h. After arrival, subjects will be prepared for blood drawing. Furthermore, three electrodes for HRV will be attached to the upper body and two electrodes for SCL will be connected to two fingers of the non-dominant hand. Both HRV and SCL will be measured continuously after attaching the electrodes until the last blood drawing. Hereafter, subjects will listen to relaxing music for about 15 min to become relaxed. Afterwards, baseline mood will be assessed by questionnaires and blood will be collected. At t=0, subjects enter a room with an unpleasant or pleasant ambiance (see 8.2.1) and get the first glass of sparkling white wine or alcohol-free sparkling white wine which they have to drink while they are watching the mood inducing scene, followed by a meal with 2 glasses of alcohol-free sparkling white wine or sparkling white wine. Subjects have to finish both the meal and wine within 20 minutes. Afterwards, subjective measures of postprandial mood will be assessed and blood will be collected repeatedly, until 150 min after the first drink. An alcohol breathing test will be performed at the end of the test day. Subjects are allowed to leave the premises if blood alcohol concentration has been measured two times <0.2‰. Subjects will stay in the room with either a pleasant or unpleasant ambiance from t=0 till they leave the premises.

11.6 In-study parameters

11.6.1 Subjective response questionnaires

The subjective response will be measured by different questionnaires, filled out on the computer. The POMS is a widely used questionnaire to determine mood. It consists of 32 items, describing the mood scales depression, anger, fatigue, vigour and tension on a 5-point scale (67). Of the 5 mood scales determined by the POMS, only vigour is measuring positive mood, therefore 2 positive mood scales are added to measure happiness and calmness (based on the 32-item Brunel Mood Scale) (24) (P9334 B11). The B-BAES is a validated questionnaire to measure the stimulating and sedating effects of alcohol (23). It consists of 6 items, which will be measured by a visual analogue scale (P9334 B12). The postprandial wellness questionnaire has been developed by Boelsma et al. (2010) to measure combined effects of satiety feelings and wellness after a meal The questionnaire consists of 16 items, measured by visual analogue scale (32) (P9334 B13). Subjective response questionnaires will be obtained every test day at <u>t=-15, t=20, t=50, t=80, t=140 min</u>

11.6.2 Blood parameters

On every test day (4 days: **session 1, 2, 3, and 4**) at <u>t = -5, 30, 60, 90, 150 min</u> for:

- Cortisol and ACTH
- Trp:LNAA ratio
- Ghrelin
- Ethanol
- Satiety markers: insulin, CCK, GLP-1
- Metabolites: glucose, FFA

On every test day (4 days: **session 1, 2, 3, and 4**) at <u>t = -5, 30, 90 min</u> for:

- Endocannabinoids

- N-acyl serotonins
- β-endorphin
- dopamine

The methods used and the applicable normal values are given in P9334 B02 and P9334 B03, respectively.

11.6.3 Non-invasive measures

Heart rate, HRV and SCL will be measured continuously on every test day (4 days days: **session 1, 2, 3, and 4**).
Heart rate and HRV will be calculated using continuous ECG monitoring. Three electrodes will be attached on the upper body for the ECG measurement (pre-cordial lead, sampling rate 512 Hz) . SCL will be measured using two Ag/AgCl electrodes, one positioned on the middle phalanx of the ring finger and one on the middle phalanx of the index finger of the non-dominant hand. All physiological data will be collected and stored using a multi-channel ambulatory system (Mobi-8, TMS International, The Netherlands), designed to measure different electro-physiological signals at the same time in both ambulatory and stationary conditions. The multi-channel system will be used wireless, the system can be carried in a small bag which will be attached to the subjects' belt. In this way, the subjects can move freely and will not notice much of the measurements. After storage the data will be analyzed using Matlab algorithms: Heart Rate and HRV based on R-top detection, SCL based on changes in tonic levels over the procedure. These measurements will be prepared, performed and analyzed under supervision and with assistance of Dr. Victor Kallen and Marc Grootjen from TNO Soesterberg, who have extensive experience with skin conductance, ECG measurements and the multi-channel system (69-71).

11.6.4 Adverse events

AEs will be established by the medical investigator on basis of:
1. Answer to the open question: 'How are you feeling?'.
2. Spontaneous reporting
3**.** Well-being questionnaire filled-in by the subjects at the start and end of every visit (Form P9334 F03)

AEs will be registered under the responsibility of the medical investigator on SBJ/006 F01.
Additional tests on medical indication can be performed, if required as assessed by the medical investigator.

11.6.5 Serious Adverse Events (SAEs)

Any serious adverse event, whether or not related to the study treatment will be reported by the medical investigator immediately (within 24 hours) to the principal investigator, the sponsor or its representatives, or to the central medical committee (CCMO; www.toetsingonline.nl), the TNO-management, the general practitioner and the study subject. The exception to this rule is that serious adverse events that have already been treated by the general practitioner or medical specialist, or about which the general practitioner or medical specialist have already been informed, which situation is to be judged by the medical investigator, do not have to be reported to the general practitioner or subject. A written report on the event will be sent to the sponsor and the medical ethics committee (MEC) within 3 working days. A definition of a serious adverse event is given in P9334 B05.

11.6.6 Compliance with study instructions and treatment

Compliance with study instructions (e.g. pill intake, refrain from alcohol 24h before testing) will be registered on an in-study checklist (P9334 F05).

11.6.7 In-study samples and analyses remark

A large number of blood analyses are indicated to be performed. The actual number and precise analyses performed will be determined when the study is conducted. As stated in chapter 5 and 6 the study is hypotheses driven. Some new parameters might be interesting to be analyzed and some might not be so interesting, based on on-going research. The number of samples analyzed will be determined later. Most variables will be measured in all subjects therefore samples will be collected of all subjects at all moments a priori. However, due to budget and sensitivity of some measurements it may be possible to perform analyses with fewer samples.

11.7 Safety of subjects

The medical investigator will guard the medical safety of the subjects within the frame work of the study. She is on call 24 hours per day.
During visits with blood collection, blood will be collected by an authorized person (authorized by the medical investigator), and a registered nurse or the (assistant) medical investigator will be present in the research facility.

11.8 Last assessment

At the last day of the study (or within one week after the clinical part) subjects will have an exit interview with the medical investigator only when requested by the subject and/or medical investigator.

11.9 Criteria for withdrawal or premature discontinuation

Subjects may discontinue the trial at any moment without the obligation to state the reason for discontinuation.
Subjects may be withdrawn from the study by the principal investigator if they do not comply with the rules and regulations of the study.
Subjects may be withdrawn from the study by the medical investigator in case of reported serious adverse events or in case of other medical/social/psychological events as evaluated by the medical investigator and discussed with the principal investigator.

In accordance to section 10, subsection 1, of the WMO, the investigator will inform the subjects and the reviewing accredited MEC if anything occurs, on the basis of which it appears that the disadvantages of participation may be significantly greater than was foreseen in the research proposal. The study will be suspended pending further review by the accredited MEC, except insofar as suspension would jeopardise the subjects' health. The principal investigator will take care that all subjects are kept informed.

Each subject who does not complete the study for any reason will have a last assessment interview, if willing to cooperate.

11.10 Payment of the subjects

- Subjects who only visit the oral information session will not be paid.
- Subjects who are screened, but who are not eligible for participation will receive €15,-

- Subjects who are stand-by until Day 01 of the study (reserves) receive €35 (including screening).
- Subjects who complete the study will receive €460,- (including screening)

All amounts are excluding travel expenses; travel expenses will be calculated on reported postal code and therefore differs for each participant with a maximum of €15,- per visit.

After completion of the study, subjects will fill in a complete declaration form, at the last day of the study. Subjects who dropped out during the study will be paid a sum in accordance with the procedures listed in P9334 F01. Subjects will be informed about the payment orally and in writing.

12 Code and handling of samples

12.1 Sample coding

In-study blood samples collected will be coded as follows: Study code (9334), followed by a slash ('/'), followed by the entry number [2 digits], followed by a slash ('/'), followed by the visit time code (VTC, P9334 B01), followed by (next line) tube code for type of matrix and volume.

The next tube codes will be used:

01-Stolbl	Serum (5 mL)
02-EDTAbl	EDTA blood (2 mL)
21-EDTAbl	EDTA blood (6 mL)
22-EDTAbl (2x)	EDTA blood (4 mL)
26-Li-hepbl	Li-heparin blood (4 mL)

For sub-sample tubes, on the second line the tube code, type of matrix and type of analyses will be indicated.

12.2 Sample collection, preparation, storage and transfer

12.2.1 Sample collection in-study

More specific information about the handling and storage of the samples, the division of the plasma and serum will be given in detail in appendix P9334 B04.

Collection of venous blood samples and subsequent processing and storage

Session	ml (tot ml)	Markers	required	Responsible
1, 2, 3 and 4	4 (80)	ACTH, ethanol	EDTA plasma	M. van Lipzig/ V. Gielen
	5 (100)	Insulin, glucose, cortisol, FFA	Serum	V. Gielen
	4 (80)	Trp:LNAA ratio	Li-Heparin plasma	F. van Schaik
	4 (80)	CCK-8, ghrelin	EDTA plasma + aprotinin	M. van Lipzig
	6 (72)	Endocannabinoids, N-acyl serotonins Dopamine, β-endorphin.	EDTA plasma + PMSF+URB602	L. Coulier
	2 (40)	GLP-1	EDTA plasma + DPP-IV	M. van Lipzig
Total	452 mL			

12.2.2 Registration of sample storage and transfer

Place of sample storage will be registered with a TNO Unique Identification number (TUI number). Sample transfer of in-study samples will be registered on forms.

12.3 Remainder samples and archive samples

Remainder of serum and plasma of the study is meant for repeated analysis in case of unexplained abnormal values and will be discarded ca. 4 weeks after reporting to the principal investigator.
Archive samples (see chapter 20) of the study period will be discarded three months after approval of the final report of the study by the sponsor and or after acceptance of scientific publication(s) concerning the study.

If necessary, archive samples will be used for analysis of markers related to the hypothesis of this study (i.e. other plasma neurotransmitters or hormones related to mood and satiety).

13 Documentation

The documentation of this study consists of the study protocol, correspondence, report **on the results only**, raw data, source documents or authenticated copies of these.

For privacy reasons, documents containing data of individual subjects will be identified only by their pre-entry or entry number. According to VOE/CLT/019 TNO has one key document in which the name of the subject is connected to pre-entry number and entry number.

14 Data management and statistics

14.1 Data management

The following datasets will be prepared:
1. **Pre-screening parameters** (age, body weight, height, BMI, body fat percentage, heart rate, diastolic blood pressure, systolic blood pressure, DMQ-R, STAI and DEBQ score.)

Data at the end of each study day (**session 1, 2, 3, and 4**), at t = -15, 20, 50, 80 and 140 min:
2. **Subjective response** (questionnaires): POMS, B-BAES, PPW.

Data at the end of each study day (**session 1, 2, 3, and 4**), at t = -5, 30, 60, 90 and 150 min:
3. **Stress hormones**: cortisol and ACTH
4. **Trp:LNAA ratio**
5. **Ghrelin response**
6. **Satiety hormones**: CCK, GLP-1, insulin
7. **Metabolites**: glucose, FFA

Data at the end of each study day (**session 1, 2, 3, and 4**), at t = -5, 30, 90 min:
8. **Endocannabinoids**
9. **N-acyl serotonins**
10. **β-endorphin**
11. **Dopamine**

Data at the end of each study day (**session 1, 2, 3, and 4**), measured continuously:
12. **Non-invasive measures**: heart rate, HRV and SCL

Raw data of body weight, height, body fat percentage, heart rate, diastolic blood pressure and systolic blood pressure will be delivered on paper and entered in an electronic format using double data-entry.
The entry of adverse events will be hand-checked by the medical investigator instead of using double data-entry.

Questionnaires (dataset 02) will be filled out on the computer and delivered in excel format.

All other raw data will be delivered in electronic format / on-line.
Raw data will be registered and will be converted to SAS data. The datasets will be checked on missing and double data, on the range of the data and on correct read in and calculations of the variables. All transformations, calculations and changes (if any) in datasets will be controlled and registered.

14.2 Missing values

After completion of the clinical part of the study, the principal investigator will decide what has to be done with missing values and will inform data management. Decisions will be described in the report.

14.3 Statistical analysis

14.3.1 Research questions / hypotheses

Primary hypothesis
It is hypothesized that moderate alcohol consumption with a meal in different ambiances changes the response of subjective and physiological parameters of mood.

Ho: there is no effect of alcohol consumption and ambiance on mood
H1: there is an effect of alcohol consumption and ambiance on mood

Secondary hypothesis
It is hypothesized that moderate alcohol consumption with a meal in different ambiances changes the response of other physiological parameters related to mood, which are not part of the primary objective.

H0: there is no effect of alcohol consumption and ambiance on mood
H1: there is an effect of alcohol consumption and ambiance on mood

14.3.2 Descriptive parameters
Subject characteristics will be presented descriptively.

14.3.3 Derived parameters
Areas under the curve (AUC) will be calculated for the parameters of the datasets 03, 04 and 05 (see par. 14.1). The AUC will be corrected for the concentration at the first measurement point. The AUC parameters will be used in the statistical analysis. Based on results and discussion with co-researchers additional AUCs may be calculated for the secondary parameters.

14.3.4 Analysis
The SAS statistical software package V8.2/V9.1 (SAS institute Inc., North Carolina, USA) will be used for statistical analysis.

For the subjective and physiological response over time, treatment effects will be investigated with an ANOVA taking into account the model factors: treatment ‘alcohol’, treatment ‘ambiance’, time (5 time points), the interaction between the treatments and between treatments and time. The ANOVA table is given on the next page.

ANOVA table

Source of variation	*Degrees of freedom (n=28)*
Subjects	*27*
Period	*3*
Subject.Period Stratum	
Alcohol	*1*
Ambiance	*1*
*Ambiance * Alcohol*	*1*
Residual	*78*
Time	*4*
*Alcohol * Time*	*4*
*Ambiance * Time*	*4*
*Ambiance * Alcohol * Time*	*4*
Residual error	*432*
Total	*559*

The AUC measurements of the physiological response will be analysed with an ANOVA (see below).

ANOVA table

Source of variation	*Degrees of freedom (n=28)*
Subjects	*27*
Period	*3*
Subject.Period stratum	
Alcohol	*1*
Ambiance	*1*
*Ambiance * Alcohol*	*1*
Residual error	*78*
Total	*111*

Based on results and discussion with co-researchers, sponsor and remarks of referee's additional statistical analysis may be performed. In all statistical tests performed, the null hypothesis (no treatment effect) will be rejected at the 0.05 level of probability.

15 Reporting

The CRO will draft a report on the clinical study and the primary outcome parameters only. This report will include aspects with regard to the used study substances, ethical aspects (list of members MEC, date of approval of the protocol and, if relevant, amendments by the MEC, details with regard to subject information and consent) and the allocation to treatment of each subject. In addition deviations from the protocol and safety data will be presented in tables and implications for the study will be briefly described. Results of the primary outcome parameters as specified in Chapter 6 of the protocol will be presented in summarizing tables. No individual data will be presented.

The final report will contain an audit certificate by the Quality Assurance Unit (QAU) of TNO Zeist and a statement on GCP compliance signed by the principal investigator.

16 Public disclosure

Policy concerning the public disclosure of the study and its results has been determined in mutual agreement between the sponsor and TNO. Neither party will unreasonably withhold the public disclosure of study results.

The following procedure is applicable:

Public disclosure may include:
- Registration of the study in a clinical trial register (clinicaltrials.gov);
- Summarized anonymous study results issued on a TNO website, sponsors' website or an international results website (clinicaltrials.org);
- Oral or poster presentation at conferences, symposia or other public meetings;
- Or full publication in peer-reviewed scientific journals.

- Anonymous results of the study will be publicly disclosed either by TNO or the sponsor within 3 months after the final report has been issued.

- In case of reasonable arguments (e.g., registration procedures) a party may prolong this period up to 6 months.

- Either the sponsor or TNO is entitled to examine any form of public disclosure prior to submission.

17 Timing

Provided approval of the MEC is obtained in Week 39 of 2011, the planned time schedule will be as follows:

- Week 37, yy : Start recruitment subjects
- Week 39, yy : Start selection subjects
- Week 43, yy : Study initiation clinical part
- Week 50, yy : Completion clinical part
- Week 10, yy : Completion laboratory analyses
- Week 14, yy : Completion statistical analyses
- Week 18, yy : Draft unaudited TNO report

The actual start and termination dates of the study will be recorded in the final report.

<u>Frequency of communication:</u>
The progress of the study will be reported to the sponsor every 3-4 months at the following stages, in principle by telephone or e-mail

- Approval of the protocol by the MEC
- Start recruitment
- Start screening
- Completion recruitment and selection
- Start, progress and end of clinical part of the study
- Results of the study
- Publication of results

18 Insurance of subjects

In this study, TNO will act as ‘uitvoerder’ (researcher) in the sense of the WMO, and the SAR will act as ‘verrichter’ (conductor).
At the request of the SAR, TNO has insured the volunteers in this study in accordance with the legal requirements in the Netherlands (Article 7 WMO and the Measure regarding Compulsory Insurance for Clinical Research in Humans of 23rd June 2003). This insurance provides cover for damage to research subjects through injury or death caused by the study for damage or death caused by the participation in this study.

The insurance company is:

Name	: HDI-Gerling Verzekeringen N.V.
Address	: Postbus 925<br>3000 AX Rotterdam
Tel.	: + 00 31 10 40 36 100
Fax.	:+ 00 31 10 40 36 275
Policy nr.	: 67-410035-16

The insurance covers damage with a maximum of € 450 000.- per subject, with a maximum of € 3 500 000.- for the whole study and is limited to € 5 000 000.- per insurance year. The insurance applies to the damage that becomes apparent during the study or within 4 years after the end of the study.
Exclusion criteria and insurance conditions will be presented in the written information for volunteers.

The insurance certificate is specified for this study and added in appendix P9334 B08. The insurance policy is a continuous insurance; therefore deviations in study period will not affect the study insurance.

TNO has also insured the subjects for their stay, their travelling to and from TNO with a collective casualty insurance, respectively a general liability insurance. The insurance certificate is present at TNO.

Dutch law will prevail in case of legal dispute with a volunteer.

The volunteers will be informed how to handle in case of damage in the written information for volunteers (Appendix P9334 F01).

19 Ethics and quality

19.1 General

The study will be conducted in compliance with the protocol and all amendments to the protocol. The protocol and all amendments to the protocol effecting the design, rationale or objectives of the study, or the burden of or health risks for the volunteers will only be implemented after written approval of the Medical Ethics Committee and the sponsor. All amendments will be sent to the Medical Ethics Committee for information or approval.

The study will be conducted according to:
1. The current revision of the World Medical Association Declaration of Helsinki. 59th WMA General Assembly, Seoul, October 2008. Note of clarification on paragraph 29 added by the WMA General Assembly, Washington, 2002. Note of clarification on paragraph 30 added by the WMA General Assembly, Tokyo 2004;
2. The ICH Guidelines for Good Clinical Practice (ICH Topic E6, adopted 01-05-1996 and implemented 17-01-1997);
3. The requirements as described in the EU Clinical Trials Directive 2001/20/EC transposed in the Revision of the Dutch Medical Research involving Human Subjects Act (“Wet medisch-wetenschappelijk onderzoek met mensen”, WMO, effective as of March 1, 2006);
4. The current national regulations.

19.2 Good Clinical Practice

TNO Zeist has been inspected by the Public Health Inspectorate for compliance with the principles of Good Clinical Practice for the conduct of clinical trials according to the EC directive 91/507/EC and the ICH Guidelines for Good Clinical Practice (CPMC)/ICH/135/95). A written statement is supplied in P9334 B06.

19.3 Quality assurance and monitoring

It is TNO policy that the Quality Assurance Unit (QAU) of TNO Zeist will audit the clinical studies. The audit certificate of the QAU will specify the dates of audits and reports to management and to the principal investigator.
Members of the MEC, representatives of the sponsor and regulatory authorities may conduct inspections of the testing facility and/or the raw data and will have direct access to medical records of participants as produced and filed by the TNO Medical Investigator. To prevent interference with the study procedures, appointments for visits are requested in advance.

20 Retention of records, samples and specimens

The following documents will be retained in the archives of TNO Zeist, during 15 years after reporting of the study:

1. Master copies of the approved study protocol and final report
2. All documents containing personal data of individual trial subjects
3. Raw data (source documents or copies of these)
4. Correspondence
5. All other information related to tests and analyses conducted

The following samples and specimens will be retained in appropriate facilities of TNO Zeist.

1. A representative part of the test substance will be retained for 5 years if its nature allows it.
2. Archive samples of blood will be retained for three months after the final report has been approved by the sponsor and after acceptance of scientific publication(s) concerning the study (See 12.3).

21 References

1. Wells, A. S. & Read, N. W. (1996) Influences of fat, energy, and time of day on mood and performance. Physiology & Behavior 59: 1069-1076.

2. Spring, B., Maller, O., Wurtman, J., Digman, L. & Cozolino, L. (1982) Effects of protein and carbohydrate meals on mood and performance: Interactions with sex and age. Journal of Psychiatric Research 17: 155-167.

3. Evans, S. M. (2007) The role of estradiol and progesterone in modulating the subjective effects of stimulants in humans. Experimental and Clinical Psychopharmacology 15: 418-426.

4. Eertmans, A., Victoir, A., Notelaers, G., Vansant, G. & Van den Bergh, O. (2006) The Food Choice Questionnaire: Factorial invariant over western urban populations? Food Quality and Preference 17: 344-352.

5. Fotopoulos, C., Krystallis, A., Vassallo, M. & Pagiaslis, A. (2009) Food Choice Questionnaire (FCQ) revisited. Suggestions for the development of an enhanced general food motivation model. Appetite 52: 199-208.

6. Dockray, S. & Steptoe, A. (2010) Positive affect and psychobiological processes. Neuroscience & Biobehavioral Reviews 35: 69-75.

7. Wansink, B. (2004) Environmental factors that increase the food intake and consumption volume of unknowing consumers. Annu. Rev. Nutr. 24: 455-479.

8. Nijs, K., de Graaf, C., van Staveren, W. A. & de Groot, L. C. P. G. (2009) Malnutrition and Mealtime Ambiance in Nursing Homes. Journal of the American Medical Directors Association 10: 226-229.

9. Stroebele, N. & De Castro, J. M. (2004) Effect of ambience on food intake and food choice. Nutrition 20: 821-838.

10. Baum-Baicker, C. (1985) The psychological benefits of moderate alcohol consumption: A review of the literature. Drug and Alcohol Dependence 15: 305-322.

11. Peele, S. & Brodsky, A. (2000) Exploring psychological benefits associated with moderate alcohol use: a necessary corrective to assessments of drinking outcomes? Drug and Alcohol Dependence 60: 221-247.

12. Tucker, J. A., Vuchinich, R. E. & Sobell, M. B. (1982) Alcohol's Effects on Human Emotions: A Review of the Stimulation/Depression Hypothesis. Subst Use Misuse 17: 155-180.

13. Persson, L. O., Sjöberg, L. & Svensson, E. (1980) Mood effects of alcohol. Psychopharmacology 68: 295-299.

14. Sher, K. J. (1985) Subjective effects of alcohol: the influence of setting and individual differences in alcohol expectancies. J Stud. Alcohol 46: 137-146.

15. Pliner, P. & Cappell, H. (1974) Modification of affective consequences of alcohol: A comparison of social and solitary drinking. Journal of Abnormal Psychology 83: 418-425.

16. Markus, C. R., Sierksma, A., Verbeek, C., van Rooijen, J. J. M., Patel, H. J., Brand, A. N. & Hendriks, H. F. J. (2004) Moderate whisky consumption in combination with an evening meal reduces tryptophan availability to the brain but does not influence performance in healthy volunteers. British Journal of Nutrition 92: 995-1000.

17. Lloyd, H. M. & Rogers, P. J. (1997) Mood and cognitive performance improved by a small amount of alcohol given with a lunchtime meal. Behav. Pharmacol. 8: 188-195.

18. Gentry, R. T. (2000) Effect of Food on the Pharmacokinetics of Alcohol Absorption. Alcoholism: Clinical and Experimental Research 24: 403-404.

19. Ramchandani, V. A., Kwo, P. Y. & Li, T. K. (2001) Effect of food and food composition on alcohol elimination rates in healthy men and women. The Journal of Clinical Pharmacology 41: 1345-1350.

20. Roine, R. (2000) Interaction of Prandial State and Beverage Concentration on Alcohol Absorption. Alcoholism: Clinical and Experimental Research 24: 411-412.

21. Martin, C. S., Earleywine, M., Musty, R. E., Perrine, M. W. & Swift, R. M. (1993) Development and Validation of the Biphasic Alcohol Effects Scale. Alcoholism: Clinical and Experimental Research 17: 140-146.

22. Nyenhuis, D. L., Yamamoto, C., Luchetta, T., Terrien, A. & Parmentier, A. (1999) Adult and geriatric normative data and validation of the profile of mood states. J. Clin. Psychol. 55: 79-86.

23. Rueger, S. Y., McNamara, P. J. & King, A. C. (2009) Expanding the Utility of the Biphasic Alcohol Effects Scale (BAES) and Initial Psychometric Support for the Brief-BAES (B-BAES). Alcoholism: Clinical and Experimental Research 33: 916-924.

24. Lane, A. M., Soos, I., Leibinger, E., Karsai, I. & Hamar, P. (2007) Validity of the Brunel Mood Scale for use with UK, Italian and Hungarian athletes. In: Mood and Human Performance: Conceptual, Measurement and Applied Issues (Lane, A. M. ed.), pp. 119-130. Nova Science Publishers, Inc., New York.

25. King, A. C., Volpicelli, J. R., Frazer, A. & O'Brien, C. P. (1997) Effect of naltrexone on subjective alcohol response in subjects at high and low risk for future alcohol dependence. Psychopharmacology 129: 15-22.

26. Holdstock, L. & de Wit, H. (2001) Individual Differences in Responses to Ethanol and d-Amphetamine: A Within-Subject Study. Alcoholism: Clinical and Experimental Research 25: 540-548.

27. Van Tilburg, M. A. L. & Vingerhoets, A. J. J. M. (2002) The effects of alcohol on mood induced by an emotional film: A study among women. Journal of Psychosomatic Research 53: 805-809.

28. Levenson, R. W., Sher, K. J., Grossman, L. M., Newman, J. & Newlin, D. B. (1980) Alcohol and stress response dampening: Pharmacological effects, expectancy, and tension reduction. Journal of Abnormal Psychology 89: 528-538.

29. McCollam, J. B., Burish, T. G., Maisto, S. A. & Sobell, M. B. (1980) Alcohol's effects on physiological arousal and self-reported affect and sensations. Journal of Abnormal Psychology 89: 224-233.

30. Lukas, S. E. & Mendelson, J. H. (1988) Electroencephalographic activity and plasma ACTH during ethanol-induced euphoria. Biological Psychiatry 23: 141-148.

31. Heishman, S. J., Arasteh, K. & Stitzer, M. L. (1997) Comparative Effects of Alcohol and Marijuana on Mood, Memory, and Performance. Pharmacology Biochemistry and Behavior 58: 93-101.

32. Boelsma, E., Brink, E. J., Stafleu, A. & Hendriks, H. F. J. (2010) Measures of postprandial wellness after single intake of two protein-carbohydrate meals. Appetite 54: 456-464.

33. Tsigos, C. & Chrousos, G. P. (2002) Hypothalamic-pituitary-adrenal axis, neuroendocrine factors and stress. Journal of Psychosomatic Research 53: 865-871.

34. Inder, W. J., Joyce, P. R., Wells, J. E., Evans, M. J., Ellis, M. J., Mattioll, L. & Donald, R. A. (1995) The acute effects of oral ethanol on the hypothalamic-pituitary-adrenal axis in normal human subjects. Clinical Endocrinology 42: 65-71.

35. Waltman, C., Blevins, L. S., Boyd, G. & Wand, G. S. (1993) The effects of mild ethanol intoxication on the hypothalamic-pituitary-adrenal axis in nonalcoholic men. Journal of Clinical Endocrinology & Metabolism 77: 518-522.

36. Zimmermann, U., Spring, K., Kunz-Ebrecht, S. R., Uhr, M., Wittchen, H. U. & Holsboer, F. (2004) Effect of Ethanol on Hypothalamic-Pituitary-Adrenal System Response to Psychosocial Stress in Sons of Alcohol-Dependent Fathers. Neuropsychopharmacology 29: 1156-1165.

37. Spaak, J., Merlocco, A. C., Soleas, G. J., Tomlinson, G., Morris, B. L., Picton, P., Notarius, C. F., Chan, C. T. & Floras, J. S. (2008) Dose-related effects of red wine and alcohol on hemodynamics, sympathetic nerve activity, and arterial diameter. American Journal of Physiology - Heart and Circulatory Physiology 294: H605-H612.

38. King, A. C., Houle, T., de Wit, H., Holdstock, L. & Schuster, A. (2002) Biphasic Alcohol Response Differs in Heavy Versus Light Drinkers. Alcoholism: Clinical and Experimental Research 26: 827-835.

39. Eckardt, M. J., File, S. E., Gessa, G. L., Grant, K. A., Guerri, C., Hoffman, P. L., Kalant, H., Koob, G. F., Li, T. K. & Tabakoff, B. (1998) Effects of Moderate Alcohol Consumption on the Central Nervous System. Alcoholism: Clinical and Experimental Research 22: 998-1040.

40. Leathwood, P. D. (1987) Tryptophan Availability and Serotonin Synthesis. Proceedings of the Nutrition Society 46: 143-156.

41. Roberto, M., Cruz, M., Bajo, M., Siggins, G. R., Parsons, L. H. & Schweitzer, P. (2010) The Endocannabinoid System Tonically Regulates Inhibitory Transmission and Depresses the Effect of Ethanol in Central Amygdala. Neuropsychopharmacology 35: 1962-1972.

42. Joosten, M., Balvers, M., Verhoeckx, K., Hendriks, H. & Witkamp, R. (2010) Plasma anandamide and other N-acylethanolamines are correlated with their corresponding free fatty acid levels under both fasting and non-fasting conditions in women. Nutrition & Metabolism 7: 49.

43. Ferrer, B., Bermúdez-Silva, F. J., Bilbao, A., Alvarez-Jaimes, L., Sanchez-vera, I., Giuffrida, A., Serrano, A., Baixeras, E., Khaturia, S. et al. (2007) Regulation of brain anandamide by acute administration of ethanol. Biochem J 404: 97-104.

44. Perra, S., Pillolla, G., Luchicchi, A. & Pistis, M. (2008) Alcohol Inhibits Spontaneous Activity of Basolateral Amygdala Projection Neurons in the Rat: Involvement of the Endocannabinoid System. Alcoholism: Clinical and Experimental Research 32: 443-449.

45. Rubio, M., McHugh, D., Fernández-Ruiz, J., Bradshaw, H. & Walker, J. M. (2007) Short-term exposure to alcohol in rats affects brain levels of anandamide, other N-acylethanolamines and 2-arachidonoyl-glycerol. Neuroscience Letters 421: 270-274.

46. Caillé, S., Alvarez-Jaimes, L., Polis, I., Stouffer, D. G. & Parsons, L. H. (2007) Specific Alterations of Extracellular Endocannabinoid Levels in the Nucleus Accumbens by Ethanol, Heroin, and Cocaine Self-Administration. The Journal of Neuroscience 27: 3695-3702.

47. Verhoeckx, K. C. M., Voortman, T., Balvers, M. G. J., Hendriks, H. F. J., Wortelboer, H. M. & Witkamp, R. F. (2011) Presence, formation and putative biological activities of N-acyl serotonins, a novel class of fatty-acid derived mediators, in the intestinal tract. Biochimica et Biophysica Acta (in press).

48. Oswald, L. M. & Wand, G. S. (2004) Opioids and alcoholism. Physiology & Behavior 81: 339-358.

49. Frias, J., Torres, J. M., Miranda, M. T., Ruiz, E. & Ortega, E. (2002) Effects of acute alcohol intoxication on pituitary-gonodal axis hormones, pituitary-adrenal axis hormones, beta-endorphin and prolactin in human adults of both sexes. Alcohol and Alcoholism 37: 169-173.

50. Dai, X., Thavundayil, J. & Gianoulakis, C. (2005) Differences in the Peripheral Levels of beta-endorphin in Response to Alcohol and Stress as a Function of Alcohol Dependence and Family History of Alcoholism. Alcoholism: Clinical and Experimental Research 29: 1965-1975.

51. Mitchell, R. L. C. & Phillips, L. H. (2007) The psychological, neurochemical and functional neuroanatomical mediators of the effects of positive and negative mood on executive functions. Neuropsychologia 45: 617-629.

52. Chuang, J. C. & Zigman, J. M. (2010) Ghrelin's Roles in Stress, Mood, and Anxiety Regulation. Int. J Pept. 2010.

53. Callahan, H. S., Cummings, D. E., Pepe, M. S., Breen, P. A., Matthys, C. C. & Weigle, D. S. (2004) Postprandial Suppression of Plasma Ghrelin Level Is Proportional to Ingested Caloric Load but Does Not Predict Intermeal Interval in Humans. Journal of Clinical Endocrinology & Metabolism 89: 1319-1324.

54. Calissendorff, J., Danielsson, O., Brismar, K. & Röjdmark, S. (2006) Alcohol ingestion does not affect serum levels of peptide YY but decreases both total and octanoylated ghrelin levels in healthy subjects. Metabolism 55: 1625-1629.

55. Calissendorff, J., Danielsson, O., Brismar, K. & Röjdmark, S. (2005) Inhibitory effect of alcohol on ghrelin secretion in normal man. European Journal of Endocrinology 152: 743-747.

56. Stacher, G., Bauer, H. & Steinringer, H. (1979) Cholecystokinin decreases appetite and activation evoked by stimuli arising from the preparation of a meal in man. Physiology & Behavior 23: 325-331.

57. Shlik, J., Zhou, Y., Koszycki, D., Vaccarino, F. J. & Bradwejn, J. (1999) Effects of CCK-4 infusion on the acoustic eye-blink startle and psychophysiological measures in healthy volunteers. Journal of Psychopharmacology 13: 385-390.

58. Benton, D. (2002) Carbohydrate ingestion, blood glucose and mood. Neuroscience & Biobehavioral Reviews 26: 293-308.

59. Romanowicz, M., Schmidt, J. E., Bostwick, J. M., Mrazek, D. A. & Karpyak, V. M. (2011) Changes in Heart Rate Variability Associated With Acute Alcohol Consumption: Current Knowledge and Implications for Practice and Research. Alcoholism: Clinical and Experimental Research 35: 1092-1105.

60. Critchley, H. D., Elliott, R., Mathias, C. J. & Dolan, R. J. (2000) Neural Activity Relating to Generation and Representation of Galvanic Skin Conductance Responses: A Functional Magnetic Resonance Imaging Study. The Journal of Neuroscience 20: 3033-3040.

61. Iwase, S., Matsukawa, T., Ishihara, S., Tanaka, A., Tanabe, K., Danbara, A., Matsuo, M., Sugiyama, Y. & Mano, T. (1995) Effect of oral ethanol intake on muscle sympathetic nerve activity and cardiovascular functions in humans. Journal of the Autonomic Nervous System 54: 206-214.

62. Spaak, J., Tomlinson, G., McGowan, C. L., Soleas, G. J., Morris, B. L., Picton, P., Notarius, C. F. & Floras, J. S. (2010) Dose-related effects of red wine and alcohol on heart rate variability. American Journal of Physiology - Heart and Circulatory Physiology 298: H2226-H2231.

63. Stritzke, W. G. K., Patrick, C. J. & Lang, A. R. (1995) Alcohol and human emotion: A multidimensional analysis incorporating startle-probe methodology. Journal of Abnormal Psychology 104: 114-122.

64. Wedel, M., Pieters, J. E., Pikaar, N. A. & Ockhuizen, T. (1991) Application of a three-compartment model to a study of the effects of sex, alcohol dose and concentration, exercise and food consumption on the pharmacokinetics of ethanol in healthy volunteers. Alcohol and Alcoholism 26: 329-336.

65. van der Gaag, M. S., van Tol, A., Scheek, L. M., James, R. W., Urgert, R., Schaafsma, G. & Hendriks, H. F. J. (1999) Daily moderate alcohol consumption increases serum paraoxonase activity; a diet-controlled, randomised intervention study in middle-aged men. Atherosclerosis 147: 405-410.

66. Pikaar, N. A., Wedel, M. & HERMUS, R. J. J. (1988) Influence of several factors on blood alcohol concentrations after drinking alcohol. Alcohol and Alcoholism 23: 289-297.

67. Nyenhuis, D. L., Yamamoto, C., Luchetta, T., Terrien, A. & Parmentier, A. (1999) Adult and geriatric normative data and validation of the profile of mood states. J. Clin. Psychol. 55: 79-86.

68. Pasman, W. J., Blokdijk, V. M., Bertina, F. M., Hopman, W. P. M. & Hendriks, H. F. J. (2003) Effect of two breakfasts, different in carbohydrate composition, on hunger and satiety and mood in healthy men. Int J Obes Relat Metab Disord 27: 663-668.

69. Westenberg, P. M., Bokhorst, C. L., Miers, A. C., Sumter, S. R., Kallen, V. L., van Pelt, J. & Blöte, A. W. (2009) A prepared speech in front of a pre-recorded audience: Subjective, physiological, and neuroendocrine responses to the Leiden Public Speaking Task. Biological Psychology 82: 116-124.

70. van Lang, N., Tulen, J., Kallen, V., Rosbergen, B., Dieleman, G. & Ferdinand, R. (2007) Autonomic reactivity in clinically referred children attention-deficit/hyperactivity disorder versus anxiety disorder. European Child & Adolescent Psychiatry 16: 71-78.

71. Brouwer, A. M., Neerincx, M. A., Kallen, V., van der Leer, L. & Brinke, M. T. (2011) EEG alpha asymmetry, heart rate variability and cortisol in response to virtual reality induced stress. Journal of Cyber Therapy and Rehabilitation 4: 27-40.

22 List of Appendices and Forms

P9334 B01	:	Visit time codes (VTCs)
P9334 B02	:	Methods of clinical laboratory tests
P9334 B03	:	Reference values of clinical laboratory tests
P9334 B04	:	Study specific blood information
P9334 B05	:	Adverse Events: definitions and codes
P9334 B06	:	Statement of GCP compliance
P9334 B07	:	Distribution list
P9334 B08	:	Insurance certificate of the study
P9334 B09	:	Product information ‘Sparkling white wine’
P9334 B10	:	Product information ‘Alcohol-free sparkling white wine’
P9334 B11	:	Profile of Mood States (POMS)
P9334 B12	:	Brief Biphasic Alcohol Effects Scale (B-BAES)
P9334 B13	:	Postprandial Wellness (PPW) questionnaire

P9334 F01	:	Written information for subjects (in Dutch) and written informed consent (in Dutch)
P9334 F02	:	Health and lifestyle questionnaire (in Dutch)
P9334 F03	:	Well-being questionnaire (in Dutch)
P9334 F04	:	Subjects checklist (pre-study)
P9334 F05	:	Subjects checklist (in-study)
P9334 F06	:	Drinking Motives Questionnaire – Revised
P9334 F07	:	State-Trait Anxiety Inventory (Trait Inventory) – form
P9334 F08	:	Dutch Eating Behaviour Questionnaire form
P9334 F09	:	Blinding questionnaire

All forms to be used by candidates and volunteers are included. In addition, other forms coded P9334 F04 and F05 will be used to register actions and compliance.

TNO Zeist

Appendix title: **Visit time codes (VTCs)**

In-study (Blood samples)

VTC	Session
101	Session 1 – 5 minutes before starting alcohol and meal
102	Session 1 – 30 minutes after starting alcohol and meal
103	Session 1 – 60 minutes after starting alcohol and meal
104	Session 1 – 90 minutes after starting alcohol and meal
105	Session 1 – 150 minutes after starting alcohol and meal
201	Session 2 – 5 minutes before starting alcohol and meal
202	Session 2 – 30 minutes after starting alcohol and meal
203	Session 2 – 60 minutes after starting alcohol and meal
204	Session 2 – 90 minutes after starting alcohol and meal
205	Session 2 – 150 minutes after starting alcohol and meal
301	Session 3 – 5 minutes before starting alcohol and meal
302	Session 3 – 30 minutes after starting alcohol and meal
303	Session 3 – 60 minutes after starting alcohol and meal
304	Session 3 – 90 minutes after starting alcohol and meal
305	Session 3 – 150 minutes after starting alcohol and meal
401	Session 4 – 5 minutes before starting alcohol and meal
402	Session 4 – 30 minutes after starting alcohol and meal
403	Session 4 – 60 minutes after starting alcohol and meal
404	Session 4 – 90 minutes after starting alcohol and meal
405	Session 4 – 150 minutes after starting alcohol and meal

TNO Zeist

Appendix title: **Methods of laboratory tests in biological fluids**

**Variable**	**Method**	
Blood chemistry		
Ethanol	Enzymatic	KLC/285
Insulin	Immunoenzymomatric assay	KLC/301
Cortisol	Immunoenzymomatric assay	KLC/302
FFA	Enzymatic	KLC/280
Other study parameters from blood (serum or plasma)		
ACTH	ELISA	manufacture's discription
Trp:LNAA ratio	Ion chromatography	manufacture's discription
Ghrelin (total)	RIA (Linco)	manufacture's description
CCK-8	RIA (Eurodiagnostica)	manufacture's description
GLP-1	RIA (Linco)	manufacture's description
β-endorphin	RIA	according to article (1)
Endocannabinoids	LC-MC	according to article (2)
Dopamine	HPLC	according to article (3)
N-acyl serotonins	to be defined	according to article (4)

References:

1. Boyer, J. C., Bancel, E., Perray, P. F., Pouderoux, P., Balmes, J. L. & Bali, J. P. (2004) Effect of champagne compared to still white wine on peripheral neurotransmitter concentrations. Int. J Vitam. Nutr. Res. 74: 321-328.

2. Balvers, M. G. J., Verhoeckx, K. C. M. & Witkamp, R. F. (2009) Development and validation of a quantitative method for the determination of 12 endocannabinoids and related compounds in human plasma using liquid chromatography-tandem mass spectrometry. Journal of Chromatography B 877: 1583-1590.

3. Jung, Y. H., Kang, D. H., Jang, J. H., Park, H. Y., Byun, M. S., Kwon, S. J., Jang, G. E., Lee, U. S., An, S. C. & Kwon, J. S. (2010) The effects of mind-body training on stress reduction, positive affect, and plasma catecholamines. Neuroscience Letters 479: 138-142.

4. Verhoeckx, K. C. M., Voortman, T., Balvers, M. G. J., Hendriks, H. F. J., Wortelboer, H. M. & Witkamp, R. F. (2011) Presence, formation and putative biological activities of N-acyl serotonins, a novel class of fatty-acid derived mediators, in the intestinal tract. Biochimica et Biophysica Acta (in press).

TNO Zeist

Appendix title: **Normal values of laboratory tests in biological fluids**

Variable	Gender	Range			Unit
Blood chemistry					
Ethanol					
Insulin	M,F			< 24.5	mU/L
Cortisol					
FFA	M,F	0.35	-	0.85	mmol/L
Other study parameters from blood (serum or plasma)					
ACTH					
Ghrelin	M,F	8.2	-	2000	pg/mL
CCK-8	M,F	0.1	-	10	pmol/L
GLP-1	M,F	10	-	50	pmol/L
Endocannabinoids	M,F`	0.1	-	100	pg/mL - ng/mL
β-endorphin					
Trp:LNAA ratio					
Dopamine					
N-acyl serotonins					

Appendix title: **Study specific blood information**

In the table below the specifics concerning blood type (plasma or serum), tube number, amount of blood and obtained on which study day, markers analysed and details about amount of dividing, handling and storage are presented.

Collection of venous blood samples and subsequent processing and storage

Session	Tube code	mL (tot mL)	Markers	Handling	Required	Lab Resp.	Divide as	Storage cond Per tube
1, 2, 3 and 4	22	4 (80)	Ethanol, ACTH	15 min, 2000xg, 4 °C	Plasma	V.J.H. Gielen / M.M.H. van Lipzig	2 x 0,5 + arch	-18 and -70° C
	01	5 (100)	Insulin, glucose cortisol, FFA	15 min, 2000xg, 4 °C	serum	V.J.H. Gielen	4 x 0,5 + arch	-18° C
	26	4 (80)	Trp:LNAA ratio	15 min, 2000xg, 4 °C	plasma	F. van Schaik	1,0 + arch	-70° C
	22	4 (80)	CCK-8, ghrelin	15 min, 2000xg, 4 °C	plasma	M.M.H. van Lipzig	1,1 + 0.25 + remainder*	-70° C
	02	2 (40)	GLP-1	15 min, 2000xg, 4 °C	plasma	M.M.H. van Lipzig	0,5 + remainder*	-70° C
	21	6 (72)	Endocannabinoids, N-acyl serotonins, B-endorphin, dopamine	15 min, 2000xg, 4 °C	plasma	L. Coulier	1,1 + 3 x0,5	-70° C
Total		452 mL						

*: remainder of serum samples will be archived.
Archives and remainders will be stored at -70 ° C.

<u>Tube codes:</u>
01 - Serum (5 mL)
02 - EDTA plasma (2 mL)
21 - EDTA plasma (6 mL)
22 - EDTA plasma (4 mL)
26 – Lithium-heparine plasma (4 mL)

Appendix title: **Adverse events: definitions and codes**

Adverse event (AE)

Any untoward medical occurrence in a subject in a clinical trial, whether or not considered related to the study treatment.

Maximum intensity

The maximum intensity of AEs is recorded in a three-point scale (code between brackets):

1. Mild (Mild) : No interference with normal functioning
2. Moderate (Mod) : No significant interference with normal functioning
3. Severe (Sev) : Significant interference with normal functioning

Serious Adverse Event (SAE)

Any untoward medical occurrence that at any study treatment:

1. Results in death,
2. Is life-threatening,
3. Is disabling,
4. Requires inpatient hospitalisation or prolongation of existing hospitalisation,
5. Results in persistent or significant disability/incapacity, or
6. Results in a congenital anomaly/birth defect.
7. Is considered important enough to be an SAE, notwithstanding the points 1 to 6

Relation to study treatment or relation to study procedure

The relation of AEs to the study treatment and relation to study procedure are recorded in a five-point scale (code between brackets):

1. Not related (Not) : No relation
2. Unlikely (Unl) : A relation is not likely, but not impossible
3. Possible (Pos) : A relation is not likely, but may exist
4. Probable (Prob) : A relation has not been clearly demonstrated, but is likely
5. Definite (Def) : A reaction which follows a reasonable temporal sequence from administration of the study treatment and which is confirmed by improvement on stopping the treatment and reappearance of the experience on repeated exposure

Subject's outcome

The subject's outcome is recorded on a four-point scale (code between brackets):

1. Recovered (Rec)
2. Still present (Pres)
3. Data not available (NA)
4. Died (Died)

Appendix title: **Adverse events: definitions and codes**

Action taken

The action taken (on study treatment in relation to the AE) is recorded on a five-point scale (code between brackets):
1. No change (NC)
2. Reduced (Red)
3. Increased (Inc)
4. Temporarily suspended (Tmp)
5. Stopped (Stop)

Start and end

Start : Actual date and time AE started

End : Actual date and time AE ended during the study period. If AE extends past end of the study, enter 'Cont'.

TNO Zeist

Appendix title: **Statement GCP compliance**

This appendix gives the GCP statement of compliance for the TNO Nutrition and Food Research Institute (TNO Voeding) by the Public Health Inspectorate, dated October 6, 1998.
As of January 2005 this TNO Institute is indicated with “TNO Quality of Life location Zeist”

INSPECTIE VOOR DE GEZONDHEIDSZORG

STATEMENT OF COMPLIANCE

WITH THE PRINCIPLES OF GOOD CLINICAL PRACTICE

It is herewith confirmed that

TNO Voeding
Utrechtseweg 48
3704 HE Zeist
The Netherlands

has been inspected to assess compliance with the Principles of Good Clinical Practice for the conduct of clinical trials according to the directive 91/507/EC and the Guideline for Good Clinical Practice (CPMP/ICH/135/95) in the period

March to July 1998

The Inspector for Clinical Trials

dr. Helena M. van den Dungen

Rijswijk, October 6, 1998

Ministry of Health, Welfare and Sports
Public Health Supervisory Service of The Netherlands
Inspectorate of Health Care

TNO Zeist

Appendix title: **Distribution list**

Intern
H.F.J. Hendriks
W.J. Pasman
A. Stafleu
I.C. Schrieks
W.A.A. Klöpping-Ketelaars (medical investigator)
J. de Groot (team manager)
F.W. Sieling
I. van Assum (Prikkamer, room 11.0.06)
J.A.M. Jacobs
H. J. Fick
C.M. Rubingh
A.E. Dutman
C. Hoeflaken
Responsible person lab (A. Speulman)
L.I.H. Bok (monsterbeheer)
V.J.H. Gielen / W. Kreuning
M.M.H. van Lipzig
F. van Schaik
L. Coulier
Quality Assurance Unit
Reserve (intern)

Extern
H. Rijkborst (SAR)
C.J. Adema (SAR)
J.W.N.M. Stasse (SAR)
H. Burghoorn (SAR)

Department of Reanimation and Clinical Toxicology, Utrecht University Hospital
METOPP
Vertrouwensarts
A. Douma (onafhankelijk persoon voor klachtenprocedure)

Reserve (extern)

Appendix title: **Isurance certificate of the study**

AON

Verzekeringscertificaat

Ondergetekenden, Aon Risk Solutions, Makelaars in Verzekeringen & Risico Adviseurs te Rotterdam, verklaren hiermede dat via ons de navolgende proefpersonenverzekering is gesloten:

**Polisnummer**	:	V0100063650 / 67-410035-16
**Verzekeringnemer**	:	Nederlandse organisatie voor Toegepast Natuurwetenschappelijk Onderzoek TNO.
**Verzekerde**	:	De proefpersoon die zich onderwerpt aan een wetenschappelijk onderzoek.
**Onderzoek**	:	"The effect of moderate alcohol consumption with a meal in different ambiances on postprandial mood, evaluated by subjective and physiological responses"<br>Studienummer 9334<br>Aan het onderzoek doen 28 personen mee.
**Onderzoeksperiode**	:	Oktober/december 2011
**Verzekerd bedrag**	:	EUR 450.000,00 per proefpersoon<br>EUR 3.500.000,00 per wetenschappelijk onderzoek<br>EUR 5.000.000,00 als maximum per jaar<br><br>EUR 5.000.000 voor de uitlooptermijn van 4 jaar in geval van beëindiging van de verzekering.
**Dekkingsgebied**	:	Nederland
**Voorwaarden**	:	Algemene voorwaarden Gerling PRO 2003.<br>De verzekering voldoet aan de in de Wet Medisch-wetenschappelijk Onderzoek met Mensen gestelde eisen
**Verzekeringsperiode**	:	1 januari 2011 tot 1 januari 2012
**Verzekerings-maatschappij**	:	HDI-Gerling Verzekeringen N.V.

Dit certificaat is afgegeven op basis van de polis onder polisnummer 67-410035-16 van Gerling en is onderworpen aan de verzekeringsvoorwaarden zoals deze in de polis c.a. vermeld zijn. Ingeval van schade is de inhoud van de polis met aanhangsels bindend.

Rotterdam, 223 augustus 2011
Aon Risk Solutions

Appendix title: **Product information** 'Sparkling white wine'

The test substance used in this study is 'Prosecco Santa Chiara', an Italian sparkling white wine. It is a vino frizzante, containing 11% alcohol.

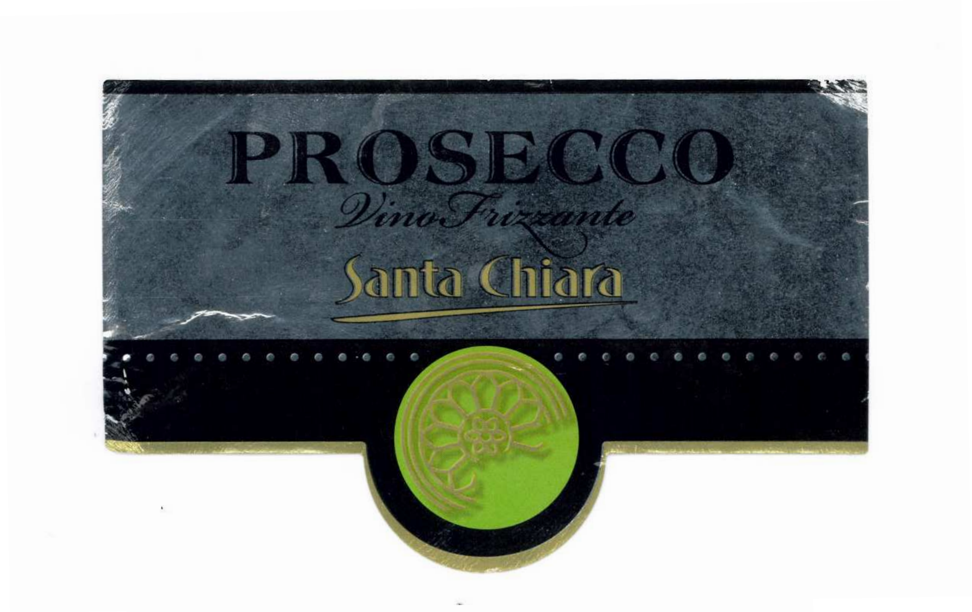

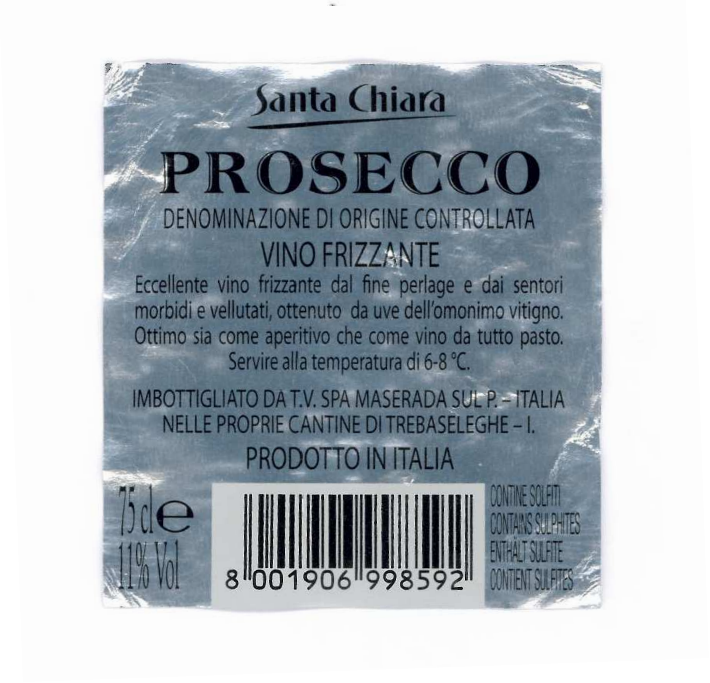

TNO Zeist

Appendix title: **Product information** ‘Alcohol-free sparkling white wine’

The reference substance used in this study is ‘Vita Nova Sparkling Secco’, an alcohol-free sparkling white wine. It is made out of Sauvignon Blanc grapes (50%) and Semillon grapes (50%) from Bordeaux, France. The Alcohol content is <0.5% (0.02% alcohol).

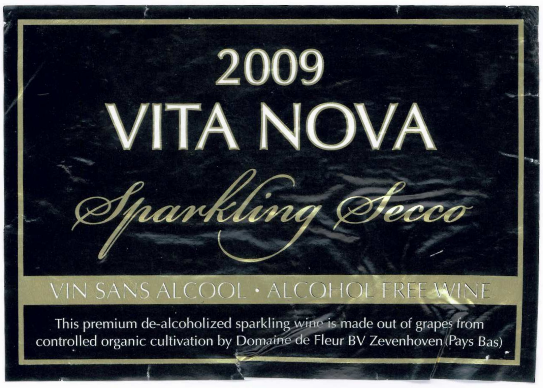

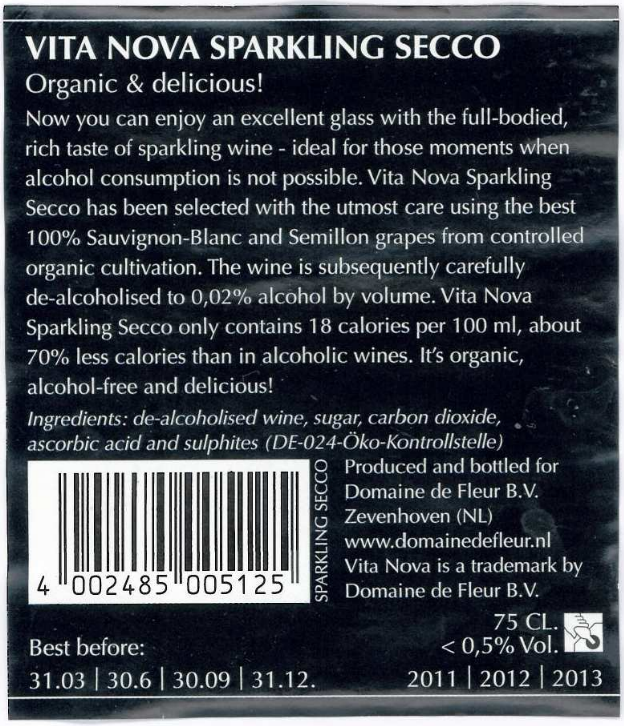

TNO Zeist

Appendix title: **Profile of mood states (POMS)**

This questionnaire will be filled out on the computer, so the instructions may differ slightly from below.

INSTRUCTIE

Hieronder vindt u een lijst met woorden. Deze woorden beschrijven stemmingen of gemoedstoestanden. Een aantal woorden beschrijven negatieve stemmingen, andere geven een positieve stemming aan. Deze gevoelens kunt u wel of niet hebben.

Lees elk woord zorgvuldig en omcirkel dan het cijfer rechts van het woord welke het beste weergeeft *HOE U ZICH NU VOELT*. Denk niet te lang na over uw antwoord. Het gaat om uw eerste indruk. Er bestaan geen foute antwoorden. Elk antwoord is goed als het maar uw eigen stemming weergeeft. Sla geen woord over.

De cijfers betekenen het volgende: 0 = helemaal niet
1 = een beetje
2 = enigszins
3 = nogal
4 = heel erg

	helemaal niet	een beetje	enigszins	nogal	heel erg
1. Neerslachtig	0	1	2	3	4
2. Vrolijk	0	1	2	3	4
3. Slecht gehumeurd	0	1	2	3	4
4. Uitgeput	0	1	2	3	4
5. Actief	0	1	2	3	4
6. Zenuwachtig	0	1	2	3	4
7. Kalm	0	1	2	3	4
8. Hulpeloos	0	1	2	3	4
9. Voldaan	0	1	2	3	4
10. Geërgerd	0	1	2	3	4
11. Helder	0	1	2	3	4
12. Paniekerig	0	1	2	3	4
13. Droevig	0	1	2	3	4
14. Opstandig	0	1	2	3	4
15. Vermoeid	0	1	2	3	4

TNO Zeist

Appendix title: **Profile of mood states (POMS)**

	helemaal niet	een beetje	enigszins	nogal	heel erg
16. Levendig	0	1	2	3	4
17. Rustig	0	1	2	3	4
18. Gespannen	0	1	2	3	4
19. Eenzaam	0	1	2	3	4
20. Tevreden	0	1	2	3	4
21. Aan het eind van mijn krachten	0	1	2	3	4
22. Ongelukkig	0	1	2	3	4
23. Woedend	0	1	2	3	4
24. Lusteloos	0	1	2	3	4
25. Vol energie	0	1	2	3	4
26. Ontspannen	0	1	2	3	4
27. Rusteloos	0	1	2	3	4
28. Onwaardig	0	1	2	3	4
29. Blij	0	1	2	3	4
30. Knorrig	0	1	2	3	4
31. Doodop	0	1	2	3	4
32. Opgeruimd	0	1	2	3	4
33. Angstig	0	1	2	3	4
34. Droefgeestig	0	1	2	3	4
35. Kwaad	0	1	2	3	4
36. Beheerst	0	1	2	3	4
37. Afgemat	0	1	2	3	4
38. Onzeker	0	1	2	3	4
39. Wanhopig	0	1	2	3	4
40. Mopperend	0	1	2	3	4

TNO Zeist

Appendix title: **Brief Biphasic Alcohol Effects Scale (B-BAES)**

This questionnaire will be filled out on the computer, so the instructions may differ slightly from below.

INSTRUCTIE

Hieronder kan je invullen in welke mate de beschreven toestand voor jou van toepassing is door op de lijn te klikken. Klikt u ver links op de lijn, dan geeft dit aan dat het niet van toepassing is en klikt u ver rechts op de lijn dan geeft dit aan dat het wel van u van toepassing is.

Energiek

Helemaal niet van toepassing	|————————|	*Helemaal wel van toepassing*

Verdoofd

Helemaal niet van toepassing	|————————|	*Helemaal wel van toepassing*

Opgewonden

Helemaal niet van toepassing	|————————|	*Helemaal wel van toepassing*

Opgewekt (up)

Helemaal niet van toepassing	|————————|	*Helemaal wel van toepassing*

Langzame gedachten

Helemaal niet van toepassing	|————————|	*Helemaal wel van toepassing*

Sloom

Helemaal niet van toepassing	|————————|	*Helemaal wel van toepassing*

TNO Zeist

Appendix title: **Postprandial Wellness (PPW) questionnaire**

This questionnaire will be filled out on the computer, so the instructions may differ slightly from below.

De onderstaande vragen worden op de volgende tijdstippen gesteld:
t=-15, t=20, t=50, t=80, t=140

We willen graag weten **hoe u zich voor en na het eten van de maaltijd voelt.**

GEEF DOOR MIDDEL VAN EEN RECHTE VERTICALE STREEP AAN
WAT UW GEVOEL HET BESTE BENADERT

Hoe prettig voelt u zich op dit moment?

Heel erg onprettig |——————————| *Heel erg prettig*

Hoe voldaan voelt u zich op dit moment?

Helemaal niet voldaan |——————————| *Heel erg voldaan*

Hoe ontspannen voelt u zich op dit moment?

Helemaal niet ontspannen |——————————| *Heel erg ontspannen*

GEEF DOOR MIDDEL VAN EEN RECHTE VERTICALE STREEP AAN WAT UW GEVOEL HET BESTE BENADERT

Hoe slaperig voelt u zich op dit moment?

Helemaal niet slaperig |————————————| *Heel erg slaperig*

Vindt u dit prettig of onprettig?

heel erg onprettig |————————————| *Heel erg prettig*

Hoe energiek voelt u zich op dit moment (lichamelijk energiek)?

Helemaal niet energiek |————————————| *Heel erg energiek*

Vindt u dit prettig of onprettig?

Heel erg onprettig |————————————| *Heel erg prettig*

GEEF DOOR MIDDEL VAN EEN RECHTE VERTICALE STREEP AAN WAT UW GEVOEL HET BESTE BENADERT

Hoe alert voelt u zich op dit moment (mentaal energiek)?

Helemaal niet alert	|———————————|	*Heel erg alert*

Vindt u dit prettig of onprettig?

Heel erg onprettig	|———————————|	*Heel erg prettig*

Hoe ervaart u uw lichaamstemperatuur op dit moment (heeft u een koud of warm gevoel)?

Heel erg koud	|———————————|	*Heel erg warm*

Vindt u dit prettig of onprettig?

Heel erg onprettig	|———————————|	*Heel erg prettig*

TNO Zeist

Appendix title: **Postprandial Wellness (PPW) questionnaire**

GEEF DOOR MIDDEL VAN EEN RECHTE VERTICALE STREEP AAN WAT UW GEVOEL HET BESTE BENADERT

Hoeveel honger heeft u op dit moment?

Geen honger |————————————————| *Heel veel honger*

Vindt u dit prettig of onprettig?

Heel erg onprettig |————————————————| *Heel erg prettig*

Hoe vol voelt u zich op dit moment?

Niet vol |————————————————| *Heel erg vol*

Vindt u dit prettig of onprettig?

Heel erg onprettig |————————————————| *Heel erg prettig*

GEEF DOOR MIDDEL VAN EEN RECHTE VERTICALE STREEP AAN WAT UW GEVOEL HET BESTE BENADERT

Hoe graag zou u op dit moment iets willen eten?

Niet graag |————————————————| *Heel erg graag*

Hoeveel zou u op dit moment kunnen eten?

Heel weinig |————————————————| *Heel veel*

De onderstaande vragen worden op de volgende tijdstippen gesteld:
t=20

GEEF DOOR MIDDEL VAN EEN RECHTE VERTICALE STREEP AAN
WAT UW GEVOEL HET BESTE BENADERT

Hoe vond u de smaak van de maaltijd?

Helemaal niet lekker |———————————————| *Heel erg lekker*

Hoe vond u de smaak van de wijn?

Helemaal niet lekker |———————————————| *Heel erg lekker*

Korte titel onderzoek: Het effect van eetsituaties en alcohol op hormonen en verzadiging

Titel bijlage: **Schriftelijke informatie voor vrijwilligers**

Titel:	Het effect van verschillende eetsituaties en verschillende hoeveelheden alcohol op hormonen en de verzadiging
Onderzoeksleider:	Henk F.J. Hendriks
Arts:	Ineke A.A. Klöpping-Ketelaars
Studie specifieke Verpleegkundige:	Henriëtte Fick-Brinkhof
Onderzoekslocatie:	TNO Humane Studies, locatie Zeist (gebouw 11)<br>Utrechtseweg 48<br>3704 HE Zeist
Postadres:	TNO Humane Studies<br>Postbus 360<br>3700 AJ Zeist
Telefoonnummer	088 86 65035
Dringende gevallen buiten kantooruren:	06 51082701
Arts buiten kantooruren	06 51082701

VERTROUWELIJKHEID

Deze schriftelijke informatie voor vrijwilligers is het eigendom van TNO (locatie Zeist) en wordt verstrekt aan diegenen, die direct of indirect betrokken zullen zijn bij het onderzoek.

Niets uit dit document mag worden vermenigvuldigd en/of openbaar gemaakt door middel van druk, fotokopie, microfilm of op welke andere wijze dan ook.

1 Inleiding

1.1 TNO

TNO staat voor Nederlandse Organisatie voor Toegepast Natuurwetenschappelijk Onderzoek en is opgericht in 1932. TNO maakt de resultaten van natuurwetenschappelijk onderzoek toepasbaar voor de samenleving. Het onderzoek dat hierbinnen wordt uitgevoerd, vindt plaats op verschillende locaties waarvan Zeist er één is. In het vervolg van dit document zal TNO locatie Zeist worden aangeduid met TNO.

1.2 Onderzoek bij TNO in Zeist

TNO doet onderzoek naar de effecten van voeding, (voedings)supplementen en soms ook farmaceutische producten op de gezondheid van de mens. Dit gebeurt in onderzoeken met vrijwilligers .We testen dan onder meer de verdraagbaarheid, veiligheid, opname, uitscheiding en effecten van voedingsstoffen op de gezondheid.
Deze onderzoeken kunnen worden uitgevoerd op basis van subsidies van de overheid of in opdracht van (en dus betaald door) bedrijven.
Daarnaast wordt er onderzoek gedaan naar chemische verbindingen in de werksituatie, en worden er behandelingen en/of meetmethodes onderzocht.

2 Reden van het onderzoek

In dit onderzoek willen we het effect van het gebruiken van een maaltijd in verschillende eetsituaties in combinatie met het drinken van alcohol variërend in alcoholpercentage onderzoeken.
Hormonen spelen een belangrijke rol in het ontstaan van verzadigingsgevoelens. In veel voorgaande onderzoeken waarbij het ontstaan van dit verzadigingsgevoel is onderzocht, zijn metingen gedaan in een laboratoriumachtige omgeving. Hormoonspiegels veranderen wanneer je een maaltijd eet, maar worden ook beïnvloedt door de omgeving. Wij denken daarom dat de hele eetsituatie belangrijk is voor de verzadiging. Daarnaast kan alcohol hormoonspiegels en verzadigingsgevoelens beïnvloeden. Daarom willen we het effect van verschillende eetsituaties in combinatie met verschillende hoeveelheden alcohol onderzoeken.

3 Doel van het onderzoek

Dit onderzoek heeft tot doel het effect van een maaltijd in combinatie met verschillende hoeveelheden alcohol op hormonen en verzadiging te meten in verschillende eetsituaties.

4 Proefopzet

Aan het onderzoek nemen 28 vrouwelijke, gezonde vrijwilligers deel. Het betreft een zogenaamde 'cross-over' studie. Dat betekent dat alle deelnemers alle eetsituaties na elkaar krijgen maar in verschillende volgorde. Deze volgorde is gerandomiseerd, wat inhoud dat de volgorde van de eetsituaties door middel van loting zal worden bepaald.

Wij kunnen u niet vertellen wat de precieze eetsituaties zullen zijn, maar tijdens deze eetsituaties zal de maaltijd elke keer hetzelfde zijn, namelijk een macaronischotel. Voor en na de maaltijd wordt een aantal keer bloed afgenomen, wordt huidgeleiding, hartslag en hartslagvariabiliteit gemeten, en moet u een aantal vragenlijstjes invullen op de computer.

5 Uitvoering van het onderzoek

5.1 Selectie

Voordat u kunt deelnemen aan het onderzoek vindt een selectie plaats aan de hand van een lijst met toelatings- en uitsluitingscriteria (bijlage 1). Tijdens de selectie wordt ook uw lengte, gewicht, vetpercentage, hartslag en bloeddruk gemeten.

5.2 Oefensessie

Tijdens de selectiedag vindt er ook een oefensessie plaats, waarbij u kunt oefenen met de vragenlijsten, die tijdens het onderzoek worden afgenomen. Ook krijgt u een macaronimaaltijd mee naar huis, zodat u deze thuis kan proeven. Dan weet u wat u kunt verwachten tijdens de testdagen.

5.3 Tijdsplanning en duur van het onderzoek

Het onderzoek vindt plaats in het onderzoekscentrum van TNO te Zeist.
Het gehele onderzoek duurt 5 weken, waarin u 4 maal naar TNO komt tussen 16:00 en 21:00 uur. Het onderzoek zal worden uitgevoerd van 24 oktober t/m 16 december 2011.

5.4 Onderzoeksproducten en behandelingen

Voor het onderzoek wordt van u verwacht dat u 4 keer naar TNO te Zeist komt voor een testdag.

Om praktische redenen zult u ingedeeld worden in groepen. Afhankelijk van de groep waarin u wordt ingedeeld komt u waarschijnlijk op steeds dezelfde dag van week naar TNO (bijvoorbeeld altijd op maandag). Deze dag zal gedurende het gehele onderzoek hetzelfde blijven.

U kunt tijdens de stopweek van de pil niet ingepland worden voor een testdag. U kunt er ook voor kiezen uw stopweek over te slaan door gelijk met een volgende pilstrip te beginnen, dan kunt u wel ingepland worden voor een testdag.

Tijdens het onderzoek maakt u verschillende eetsituaties mee. Het eten en drinken dat u krijgt is in de winkel verkrijgbaar en bestaat uit een avondmaaltijd met 3 glazen mousserende witte wijn. De maaltijd is alle vier de keren hetzelfde, maar de mousserende witte wijn kan variëren in alcoholpercentage. Voor deelname aan het onderzoek is het belangrijk dat u zowel macaroni als mousserende witte wijn lekker vindt. De macaroni en 3 glazen witte wijn bent u namelijk verplicht volledig op te eten en drinken binnen een tijdsbestek van 20 min.

Eenmalig voor en viermaal na de maaltijd wordt er bloed afgenomen en moet u een aantal vragenlijsten invullen. De huidgeleiding en hartslagvariabiliteit worden continu gemeten.

5.5 Procedures tijdens de testdagen

U komt ’s middag naar TNO op de afgesproken tijd. U vult een korte vragenlijst in over uw welbevinden waarna u wordt voorbereid op de bloedafname. Hierna zullen de elektroden voor het meten van huidgeleiding en hartslagvariabiliteit worden bevestigd. Voor de huidgeleiding worden twee elektroden aan twee vingers van uw niet-dominante hand geplakt, en voor de hartslagvariabiliteit bepaling worden elektroden bevestigd op uw bovenlichaam voor het maken van een electrocardiogram (ECG). Deze zullen er pas weer afgehaald worden aan het einde van de testdag. Hierna kunt u even ontspannen in de ‘huiskamer’. Na ongeveer 15 min moet u een aantal vragenlijstjes invullen en vindt er een bloedafname plaats. Hierna krijgt u in een kamer een kort filmpje te zien en krijgt u een glas mousserende wijn. Vervolgens krijgt u de macaronischotel met 2 glazen mousserende witte wijn, waarna u nog 4 keer de vragenlijsten invult en er nog 4 keer bloed zal worden afgenomen (verspreid over 2 uur).

Na de laatste meting zal er een blaastest worden afgenomen, om te controleren of uw alcoholpromillage laag genoeg is om zelf naar huis te kunnen gaan. Uw alcoholpromillage moet hiervoor onder de 0,2‰ zijn, dit is de alcohollimiet voor het beginnerrijbewijs. Aangezien de wijn genuttigd wordt samen met een maaltijd en de laatste meting 2,5 uur na het eerste glas plaatsvindt, zal de alcoholconcentratie in uw bloed laag zijn. Desondanks kunnen er ten gevolge van de alcoholinname bijwerking optreden, zoals een verminderd reactievermogen. **U wordt dan ook sterk afgeraden om een (motor)voertuig te besturen op weg naar huis, maar geadviseerd om gebruik te maken van het openbaar vervoer of een taxi!**

Aan het eind van de testdag vult u nog een keer de vragenlijst over uw welbevinden in. Aan het eind van de laatste testdag wordt u gevraagd nog een laatste vragenlijst in te vullen ter afsluiting van het onderzoek. Afhankelijk van hoe snel het alcoholpromillage in uw bloed zakt, zal de testdag ongeveer 4 uur duren.

In de bloedmonsters zullen hormonen en neurotransmitters (stofjes die in de hersenen bepaalde gevoelens opwekken of juist afremmen, een voorbeeld is nicotine uit sigaretten) gemeten worden die te maken hebben met verzadiging en het verzadigingsgevoel.
Ook zullen algemene stoffen in het bloed gemeten worden die invloed kunnen hebben op het verzadigingsgevoel zoals glucose en insuline.
Daarnaast zullen bloedmonsters bewaard worden ten behoeve van mogelijke latere analyses in het kader van het onderzoek. Al deze testen geven waarschijnlijk geen duidelijke informatie over hoe gezond u bent. Wel kan door grote groepen mensen te vergelijken iets worden gezegd over het effect van de onderzoeksstoffen in het algemeen.

5.6 Richtlijnen

Er zijn een aantal richtlijnen waar u zich aan moet houden voordat u een testdag heeft en op de testdag zelf:

- u mag 24 uur voordat u naar TNO komt geen alcohol meer drinken.
- u mag op de testdag geen koffie drinken na 12:00 uur ’s middags.
- u mag op de testdag niet intensief sporten of bewegen.
- u eet niets meer vanaf 2 uur voordat u bij TNO verwacht wordt (drinken mag wel).
- u ontbijt en luncht op elke testdag hetzelfde en op ongeveer hetzelfde tijdstip.

Verder is het belangrijk dat u uw eet- en drinkgewoonten, lichamelijke beweging en leefpatroon niet aanpast tijdens de studie.

Het is voor het slagen van dit onderzoek uitermate belangrijk dat u zich aan bovenstaande richtlijnen houdt.

5.7 Uitkeuring

Afhankelijk van uw wens of die van de TNO arts, heeft u een gesprek met de arts over uw huidige gezondheid en uw gezondheid tijdens het onderzoek.

5.8 Veiligheid

Tijdens het onderzoek is altijd een arts bereikbaar. De bloedafname wordt verricht door een ervaren bloedprikker en tijdens de bloedafname is een verpleegkundige of EHBO-er in het onderzoeksinstituut aanwezig.

5.9 Mogelijke bijwerkingen

Er zijn geen mogelijke bijwerkingen te verwachten.

6. Te verwachten voordelen en ongemakken

U zult als gezonde vrijwilliger geen direct voordeel ondervinden van deelname aan het onderzoek. Echter, door uw medewerking te verlenen kunt u een bijdrage leveren aan de kennis over de effecten van verschillende eetsituaties en alcohol op hormoonspiegels en verzadiging.

Als u deelneemt aan het onderzoek kunt u (eventueel) de volgende ongemakken verwachten:

- Herhaalde bloedafnames; 20 maal tijdens de studie (totaal circa 460 ml)

7 Ethische aspecten, kwaliteit en persoonlijke levenssfeer

7.1 Ethische toestemming

Dit onderzoek is positief beoordeeld door een erkende Medisch Ethische Toetsingscommissie.

7.2 Informatie en schriftelijke toestemming

U kunt pas deelnemen aan het onderzoek nadat u mondeling en schriftelijk bent geïnformeerd over de achtergronden van en procedures tijdens het onderzoek, de mogelijke risico's bij deelname aan het onderzoek en uw rechten en plichten. Ook moeten al uw vragen naar tevredenheid zijn beantwoord en moet u schriftelijk toestemming hebben gegeven voor uw deelname. Dit geeft u aan door het ondertekenen van een zogenaamd 'informed consent'. Dit formulier dient in tweevoud te worden ingevuld (één exemplaar is voor u en één exemplaar is voor TNO). Indien er nieuwe relevante informatie beschikbaar komt met betrekking tot het onderzoek zult u daarover worden geïnformeerd.
Op basis van de mondelinge en schriftelijke informatie kunt u 2 à 3 dagen bedenktijd in acht nemen.

7.3 Beëindiging deelneming

U doet geheel vrijwillig mee aan het onderzoek. U mag uw deelname aan het onderzoek op ieder moment beëindigen, en u hoeft daarbij geen reden op te geven. De arts kan uw deelname aan het onderzoek beëindigen, als hij/zij dat op medische gronden noodzakelijk vindt. De onderzoeksleider kan uw deelname beëindigen, als u zich niet aan de voorschriften houdt. De METC kan alleen op medisch en/of ethische gronden TNO en/of de sponsor toestaan dit onderzoek te beëindigen.

7.4 Onafhankelijke arts

Als u twijfelt of u wel geschikt/gezond bent om aan dit onderzoek deel te nemen kunt u met uw vragen terecht bij de arts, onderzoeksleider of de studie specifieke verpleegkundige. Als u desondanks nog met vragen blijft zitten kunt u contact opnemen met een onafhankelijke arts, Dr. E.J. van der Beek, bedrijfsarts. Hij is te bereiken op werkdagen via 070-3484558 of 06-29570823 (Achmea Vitale / Ministerie van Buitenlandse Zaken, Postbus 20061, 2500 EB, Den Haag of via e-mail: eric.van.der.beek@achmea.nl)).
Ook als u als deelnemer aan dit onderzoek medische vragen heeft die u liever aan een onafhankelijke arts wilt stellen kunt u bij hem terecht.

7.5 Persoonlijke levenssfeer en toegankelijkheid onderzoeksgegevens

Om de privacy te waarborgen, wordt het gebruik van namen zo veel mogelijk beperkt. Iedere (kandidaat-)vrijwilliger krijgt een keurings- en een onderzoeksnummer. Deze nummers worden gebruikt bij de registratie van de onderzoeksgegevens.
Tijdens het onderzoek kunnen daartoe bevoegde TNO-medewerkers, leden van een Medisch Ethische Toetsingscommissie van de METOPP te Tilburg, medewerkers van de Inspectie voor de Gezondheidszorg, en monitoren van de betrokken opdrachtgever ter plekke aanwezig zijn om inspecties en/of beoordelingen uit te voeren om de betrouwbaarheid en de kwaliteit van de meetgegevens te verifiëren waarop later de onderzoeksresultaten worden gebaseerd. De inzage in uw persoonlijke medische gegevens gebeurt steeds onder geheimhouding.
De resultaten van het onderzoek kunnen leiden tot publicaties in vaktijdschriften en voordrachten op congressen en bijeenkomsten. Uw anonimiteit blijft daarbij gewaarborgd.
Uw huisarts zal door TNO worden geïnformeerd over uw deelname aan het onderzoek, en als er afwijkende waarden zijn gevonden in het bloed of bij de andere metingen, wordt hij/zij ook geïnformeerd.

7.6 Klachten

Hoe te handelen bij klachten?
Als u klachten heeft over het onderzoek, kunt u dit melden bij de onderzoeker of de studie specifieke verpleegkundige (zie voorblad).
Als u liever uw klacht meldt bij iemand die helemaal niets met de uitvoer van het onderzoek te maken heeft, kunt u terecht bij mw. A. Douma, tel. 088 86 61 684.

8. Financiële vergoeding

- Voor deelname aan het volledige onderzoek, ontvangt u een vergoeding van **€ 460,-** inclusief 15,- voor de selectiedag.
- Voor de selectiedag ontvangt u **€ 15,-.**

- Deelnemers die reserve staan tot en met dag 1 van de studie ontvangen **€ 35,-** inclusief 15,- voor de selectiedag (zij komen of naar TNO of staan telefonisch stand-by. Zij gaan in de studie indien er mensen uitvallen of niet komen opdagen tijdens de eerste studiedag).

De hierboven genoemde bedragen zijn exclusief reiskosten. De reiskostenvergoeding bedraagt € 0,19 per kilometer, ongeacht de wijze van vervoer. Het aantal kilometers wordt op basis van uw werkelijke postcode bepaald. Er geldt een maximumbedrag van **€ 15,00** reiskosten per bezoek.

TNO is verplicht deze vergoedingen op te geven aan de Belastingdienst.
In bijlage 2 kunt u lezen welk deel van de vergoeding u ontvangt bij onvolledige deelname.

9. Verzekering

Tijdens het onderzoek bent u verzekerd tegen de gevolgen van letsel (= lichamelijke en geestelijke schade) veroorzaakt door:

1. Deelname aan het onderzoek en de voorafgaande informatie- en selectieprocedure, en
2. Gaan naar, verblijf bij en vertrek van TNO te Zeist.

Zie bijlage 3 voor meer informatie over de studiespecifieke WMO verzekering als ook de ongevallen verzekering.

Bijlage 1. Selectiecriteria

U kunt alleen aan het onderzoek meedoen als:

1. U op basis van ziektegeschiedenis en lichamelijk onderzoek geschikt blijkt te zijn voor het onderzoek
2. U vrouw bent en op dag 01 van het onderzoek tussen de 18 en 45 jaar bent.
3. U de anticonceptiepil gebruikt op dag 01 van het onderzoek. *Dit moet een éénfasige combinatiepil zijn met een stopweek of placeboweek van 7 dagen (alle pillen bevatten dezelfde hoeveelheid oestrogeen en progestageen).*
4. Uw Body Mass Index (BMI), dit is uw gewicht gedeeld door het kwadraat van uw lichaamslengte, tussen de 18.5 en 27 kg/m^2 ligt (wordt gemeten tijdens de keuring).
5. U een gewicht heeft tussen de 57 en 80 kg.
6. U een gebruikelijk Nederlands voedingspatroon volgt.
7. U minimaal 3 en maximaal 21 alcoholische dranken per week gebruikt.
8. U vrijwillig deelneemt aan het onderzoek.
9. U schriftelijk toestemming geeft voor deelname.
10. U bereid bent zich te houden aan de regels van het onderzoek.
11. U bereid bent gedurende het onderzoek niet op te treden als bloeddonor/plasmadonor.
12. U bereid bent te accepteren dat de verzamelde gegevens gecodeerd worden gebruikt, minimaal 15 jaar worden gearchiveerd en mogelijk gepubliceerd.
13. U bereid bent te accepteren dat de te ontvangen vergoeding door TNO wordt opgegeven aan de belastingdienst.

U kunt **niet** meedoen als:

1. U heeft deelgenomen aan enig onderzoek waarbij bloed is afgenomen en/of toediening van stoffen heeft plaatsgevonden in de 90 dagen voor dag 01 van dit onderzoek.
2. De resultaten van het onderzoek beïnvloed zouden kunnen worden door huidige of vroegere ziektes of klachten (hieronder vallen ziekten zoals psychologische of psychiatrische aandoeningen, hormonale aandoeningen of maag-darmziekten).
3. U medicatie gebruikt die de studie kunnen beïnvloeden (bijvoorbeeld antidepressiva).
4. U familieleden heeft met alcoholisme.
5. U in het verleden alcohol- of drugsgerelateerde problemen heeft gehad.
6. U rookt.
7. Uw gewicht onverklaarbaar is af- of toegenomen in de maand voorafgaand aan de keuring.
8. U een afslankdieet of medisch voorgeschreven dieet volgt.
9. U een vegetarisch, veganistisch of macrobiotisch dieet volgt.
10. U bloed/plasma heeft gedoneerd binnen 1 maand voor dag 01 van het onderzoek.
11. U zwanger bent, borstvoeding geeft of de wens heeft om zwanger te worden tijdens het onderzoek.
12. U een medewerker van TNO in Zeist bent of partner of familielid van een medewerker van TNO in Zeist (tot en met de tweede graad).
13. U geen huisarts heeft.
14. U niet toestaat dat de aanmelding aan dit onderzoek aan uw huisarts gemeld wordt.
15. U niet toestaat, dat gegevens met betrekking tot uw gezondheid, zoals labresultaten, bevindingen tijdens het gesprek met de arts en het lichamelijk onderzoek door de arts, en eventuele ongewenste voorvallen aan uw huisarts gemeld worden en/of niet toestaat dat informatie aan uw huisarts gevraagd wordt, als er een dringende reden is in het belang van uw gezondheid.

Bijlage 2. Financiële vergoeding bij onvolledige deelname

Bij onvolledige deelname aan het onderzoek ontvangt u:

1. De volledige vergoeding, als uw deelname wordt beëindigd door:
    1. De arts op medische gronden.
    2. De onderzoeksleider op basis van onderzoeksafwijkingen, buiten uw schuld om (ter beoordeling van de projectleider).
    3. Voortijdige beëindiging van het onderzoek door de METC na aangeven van de sponsor en/of TNO.

2. Een pro rata vergoeding, als:
    1. U uw deelname voortijdig beëindigt op grond van overmacht.*

3. Geen vergoeding, als:
    1. De projectleider voortijdig uw deelname beëindigt op grond van gebrek aan medewerking aan het onderzoek of wangedrag.
    2. U niet geschikt bent voor deelname aan het onderzoek.
    3. U niet bent ingeloot voor deelname aan het onderzoek.

* De projectleider beslist in overige gevallen van voortijdige beëindiging van deelname aan het onderzoek of u een vergoeding ontvangt en, zo ja, welk bedrag.

Bijlage 3. Aanvullende informatie over de verzekering

TNO heeft, ingevolge de Wet Medisch Wetenschappelijk Onderzoek met Mensen, respectievelijk het Besluit van 23 juni 2003 houdende regels inzake de verplichte verzekering bij medisch-wetenschappelijk onderzoek met mensen, een verzekering afgesloten voor eventuele schade van proefpersonen ten gevolge van deelname aan een onderzoek uitgevoerd door TNO op een locatie in Nederland.

De gegevens van de verzekeraar zijn:

Naam:	HDI-Gerling Verzekeringen N.V.
Postadres:	Postbus 925
	3000 AX Rotterdam
Telefoon:	010 4036100
Telefax:	010 4036275

Het polisnummer is 300017584.

Deze verzekering biedt dekking voor schade door dood of letsel tot een bedrag van € 450.000,- per proefpersoon, zulks tot een maximum van tot € 3.500.000,- per onderzoek met een maximum van € 5.000.000,- per verzekeringsjaar. Tevens geldt een uitlooptermijn van 4 jaar in geval van beëindiging van de verzekering, met een maximum verzekerd bedrag van € 5.000.000 voor alle schade tezamen die zich na deze beëindiging openbaart. Deze verzekering is uitsluitend van kracht in Nederland.

Deze verzekering dekt uitsluitend:
- Schade geleden door de proefpersoon die verhinderd is arbeid te verrichten tot maximaal € 60.000 per jaar;
- Derving van levensonderhoud tot maximaal € 60.000 per jaar;
- Kosten huishoudelijke hulp tot maximaal € 7,50 per uur;
- Vergoeding van nadeel anders dan vermogensschade > € 1.500 tot maximaal € 10.000;
- Redelijke kosten van medische hulp, medische voorzieningen etc. tot maximaal € 50.000;
- Redelijke kosten van vervoer van € 0,40 per km tot maximaal € 10.000.

Uitgesloten van dekking van deze verzekering is schade:
1. waarvan op grond van de aard van het onderzoek zeker of nagenoeg zeker was dat deze zich bij de proefpersoon zou voordoen en aan de proefpersoon als zodanig mededeling is gedaan;
2. die het gevolg is van het uitblijven van een vermindering van de gezondheidsproblemen van de proefpersoon, dan wel het gevolg is van een verdere verslechtering van de gezondheids-problemen van de proefpersoon, indien deelname van de proefpersoon aan het onderzoek geschiedt in het kader van de behandeling van deze gezondheidsproblemen;
3. waarvan aannemelijk is dat die zich ook zou hebben geopenbaard wanneer de proefpersoon niet aan het onderzoek zou hebben deelgenomen;
4. die zich bij een nakomeling van de proefpersoon openbaart als gevolg van een nadelige inwerking van het onderzoek op de proefpersoon of diens nakomeling;
5. veroorzaakt, bevorderd of verergerd doordat de verzekerde zich niet of niet volledig aan de voorschriften en instructies van de met de uitvoering van het onderzoek belaste personen (TNO) heeft gehouden.
6. In verband met onderzoeken met een operatierisico.

Schademelding
Bij letsel en letselschade of relevante gezondheidsklachten moet direct(en):
1. contact worden opgenomen met de studie specifieke verpleegkundige van TNO,
2. de adviezen van de onderzoeker van TNO worden opgevolgd,
3. er zorg voor worden gedragen dat verdere schade zo veel mogelijk wordt beperkt/voorkomen, en
4. de huisarts worden geïnformeerd.
5. Verplichtingen ten aanzien van de verzekering worden nageleefd.

Naast de wettelijke verplichte verzekering voor proefpersonen zijn enkele bedrijfsverzekeringen van TNO eveneens van toepassing op onderzoek met proefpersonen, zoals de collectieve ongevallenverzekering voor bijzondere werkomstandigheden en de aansprakelijkheidsverzekering.

Toestemmingsformulier

Ik bevestig dat ik de schriftelijke informatie voor vrijwilligers van het onderzoek "Het effect van verschillende eetsituaties en verschillende hoeveelheden alcohol op hormonen en de verzadiging" (versie 09 september 2011) heb gelezen. Ik begrijp de informatie.

Ik heb de gelegenheid gehad om aanvullende vragen te stellen. Deze vragen zijn in voldoende mate beantwoord. Ik heb voldoende tijd gehad om over deelname na te denken.

Ik weet dat mijn deelname geheel vrijwillig is en dat ik mijn toestemming op ieder moment kan intrekken zonder dat ik daarvoor een reden hoef te geven.

Ik geef toestemming om mijn huisarts op de hoogte te brengen van mijn deelname aan dit onderzoek.

Ik geef toestemming dat onderzoeksleiders, de artsonderzoeker, de verpleegkundige, medewerkers van de Inspectie voor de Gezondheidszorg, auditors van TNO, leden van de medisch-ethische toetsingscommissie en de opdrachtgever inzage kunnen krijgen in mijn medische gegevens en onderzoeksgegevens. Ik geef toestemming om de gegevens te verwerken voor de doeleinden zoals beschreven in de schriftelijke informatie.

Ik geef toestemming dat het afgenomen lichaamsmateriaal gedurende 3 maanden na goedkeuring van het studierapport door de sponsor wordt bewaard voor verdere analyse voor wetenschappelijk onderzoek in het kader van dit onderzoek en dat het daarna wordt vernietigd.

Ik stem in met mijn deelname aan bovengenoemd onderzoek.

Keuringsnummer : 9334 / ……….

Naam (voorletters, achternaam) : ____

Handtekening proefpersoon :

Datum : |___|___|-|___|___|-|___|___|___|___| (dd-mm-jjjj)

In te vullen door TNO medewerker:

Ik heb mondelinge en schriftelijke toelichting verstrekt over het onderzoek (of was aanwezig bij deze toelichting).

Naam (voorletters, achternaam) : ____

Handtekening :

Datum : |___|___|-|___|___|-|___|___|___|___| (dd-mm-jjjj)

Studiecode TNO: 9334 Keuringsnummer: «preentry»

Persoonlijke gegevens

Kandidaat

Geslacht : man / vrouw (doorstrepen hetgeen **niet** van toepassing is)

Achternaam :______________________________

Voorletters :______________________________

Roepnaam :______________________________

Straat + huisnr. :______________________________

Postcode : |___|___|___|___| |___|___|

Woonplaats :______________________________

Geboortedatum : |___|___|-|___|___|-|___|___|___|___| (dd-mm-jjjj) Leeftijd : |___|___| jaar

Geboorteplaats :______________________________

Telefoon :______________________________(privé) ______________________________(mobiel)

E-mail adres :______________________________

Huisarts

Naam :______________________________

Straat + huisnr. :______________________________

Postcode : |___|___|___|___| |___|___|

Woonplaats :______________________________

Telefoon :______________________________

Studiecode TNO: 9334 Keuringsnummer: «preentry»

Verklaring leefgewoonten en gezondheid

Korte titel onderzoek: Effect van eetsituaties op hormonen en verzadiging

Indien u wilt deelnemen aan bovengenoemd onderzoek, wordt u verzocht deze verklaring eenduidig, volledig en naar waarheid in te vullen met ***zwarte*** *pen of ballpoint* ***(geen potlood)****. De gegevens, die u invult, worden besproken met de keuringsarts en zullen vertrouwelijk worden behandeld.*

1 Algemeen

1.1 Hoogst genoten opleiding: ____

1.2 Tot welk ras rekent u zichzelf?

- ☐ blank
- ☐ negroïde
- ☐ Aziatisch
- ☐ overige
- ☐ mengvorm van: ____

1.3 Heeft u eerder meegedaan aan een onderzoek met vrijwilligers / proefpersonen?

- ☐ ja, minder dan 3 maanden geleden
- ☐ ja, meer dan 3 maanden geleden
- ☐ nee, ik heb nog nooit aan een onderzoek meegedaan

1.4 Heeft u eerder meegedaan aan een alcoholonderzoek bij TNO?

- ☐ Zo ja, wanneer?____
- ☐ Nee

Studiecode TNO: 9334 Keuringsnummer: «preentry»

1.5 Heeft u gezins- en/of familieleden die bij TNO te Zeist werkzaam zijn?

☐ ja
Wie?

☐ echtgeno(o)t(e) / partner

☐ ouder

☐ broer / zuster

☐ kind

☐ overig: ____

☐ nee → **ga verder naar vraag 1.6**

1.6 Bent u bloeddonor? (Let op: tijdens de uitvoer van het onderzoek is bloeddonatie niet toegestaan)

☐ ja
Wat is de datum van uw laatste donatie : |___|___|-|___|___|-|___|___|___|___|
(dd-mm-jjjj)

Datum volgende donatie : |___|___|-|___|___|-|___|___|___|___|
(dd-mm-jjjj)

Aard donatie: ☐ bloed

☐ plasmaferese

☐ nee → **ga verder naar vraag 2.1**

Studiecode TNO: 9334 Keuringsnummer: «preentry»

2 Genotmiddelen

2.1 Drinkt u alcohol houdende dranken?

☐ ja
Hoeveel?
☐ 1-3 glazen per week
☐ 3-6 glazen per week
☐ 7-14 glazen per week
☐ 14-21 glazen per week
☐ meer dan 21 glazen per week
☐ nee → **ga verder naar 2.2**

2.2 Rookt u?

☐ ja
☐ nee, ik ben korter dan 3 maanden geleden gestopt
☐ nee, ik ben langer dan 3 maanden geleden gestopt
☐ nee, ik heb nooit gerookt

2.3 Gebruikt u op dit moment nicotinepleisters en/of nicotinekauwgom?

☐ ja
☐ nee

2.4 Gebruikt u soft drugs en/of hard drugs?

☐ ja
☐ nee, ik ben korter dan 3 maanden geleden gestopt
☐ nee, ik ben langer dan 3 maanden geleden gestopt
☐ nee, nooit gebruikt

2.5 Komt er in de familie alcoholisme voor?

☐ ja
☐ nee

Studiecode TNO: 9334 Keuringsnummer: «preentry»

3 Slaap

3.1 Heeft u avond- en / of nachtdiensten?

☐ ja → neem contact op met de verpleegkundige of arts.
☐ nee

3.2 Heeft u slaapproblemen?

☐ nee
☐ ja

Heeft u één of meerdere vragen met ja beantwoord, licht deze vra(a)g(en) dan zonodig toe:

____

____

Studiecode TNO: 9334 Keuringsnummer: «preentry»

4 Zintuigen

Heeft u problemen met:

	ja	nee	
4.1	☐	☐	Zien (anders dan een bril of lenzen)
4.2	☐	☐	Horen (inclusief een gehoorapparaat)
4.3	☐	☐	Proeven of uw gebit
4.4	☐	☐	Ruiken

Heeft u één of meerdere vragen met ja beantwoord, licht deze vra(a)g(en) dan zonodig toe:

____

____

Studiecode TNO: 9334 Keuringsnummer: «preentry»

5 Gezondheid

A.: Heeft u regelmatig (enkele keren per maand) last van:

	ja	nee	
A 5.1	☐	☐	Hoofdpijn
A 5.2	☐	☐	Duizeligheid
A 5.3	☐	☐	Misselijkheid of braken
A 5.4	☐	☐	Hyperventilatie
A 5.5	☐	☐	Verkouden of grieperig (minstens 1 keer per maand)

Heeft u één of meerdere vragen met ja beantwoord, licht deze vra(a)g(en) dan zonodig toe:

____

____

B.: Bent u momenteel onder controle of behandeling bij een arts of fysiotherapeut voor:

	ja	nee	
B 5.1	☐	☐	Huid, haar, nagels
B 5.2	☐	☐	Zenuwen, hersenen
B 5.3	☐	☐	Suikerziekte of schildklierproblemen
B 5.4	☐	☐	Hart- en vaatziekten (bijv. hartkloppingen, pijn/druk op de borst, ritmestoornissen, lage of hoge bloeddruk)
B 5.5	☐	☐	Longen (bijv. astma, emfyseem)
B 5.6	☐	☐	Nier- of blaasklachten
B 5.7	☐	☐	Maag, lever en darmklachten (bijv. buikpijn, ontstekingen, verstopping of diarree)

Studiecode TNO: 9334 Keuringsnummer: «preentry»

	ja	nee	
B 5.8	☐	☐	Gewrichten of spieren (bijv. schouders, ellebogen, heupen enz.)
B 5.9	☐	☐	Afwijkingen in het bloed (bijv. verhoogd suiker, verhoogd cholesterol, verhoogde leverenzymen)
B 5.10	☐	☐	Psychische klachten
B 5.11	☐	☐	Overige klachten, hier niet opgenoemd

Heeft u één of meerdere vragen met ja beantwoord, licht deze vra(a)g(en) dan zonodig toe:

____

____

C.: Overig

	ja	nee	
C 5.1	☐	☐	Heeft u de laatste vijf jaar een operatie of behandeling ondergaan?
C 5.2	☐	☐	Is of wordt er binnenkort een medische behandeling of operatie bij u gepland?
C 5.3	☐	☐	Is uw gewicht in de laatste drie maanden veranderd zonder aanwijsbare oorzaak?
C 5.4	☐	☐	Bent u allergisch of overgevoelig?
C 5.5	☐	☐	Komen in uw familie veel hart- en vaatziekten voor?

Heeft u één of meerdere vragen met ja beantwoord, licht deze vra(a)g(en) dan zonodig toe:

____

____

Studiecode TNO: 9334 Keuringsnummer: «preentry»

6 Medicatie, supplementen etc.

6.1 Gebruikt u geneesmiddelen (ook “af en toe”gebruik)?

☐ ja Namelijk: ____

____

☐ nee

6.2 Gebruikt u vaker dan 1 keer per maand slaapmiddelen?

☐ ja Namelijk: ____

☐ nee

6.3 Gebruikt u vaker dan 1 keer in de twee weken pijnstillers?

☐ ja Namelijk: ____

☐ nee

6.4 Gebruikt u voedingssupplementen?

☐ ja Namelijk: ____

☐ nee

Heeft u één of meerdere vragen met ja beantwoord, licht deze vra(a)g(en) dan zonodig toe:

____

____

Studiecode TNO: 9334 Keuringsnummer: «preentry»

7 Voeding

7.1 Volgt u wat uw voeding betreft een speciale leefregel (b.v. vegetarisch, veganistisch, macrobiotisch, Islamitisch?)

☐ ja Namelijk: ____
☐ nee

7.2 Volgt u een dieet?

☐ ja Namelijk: ____

Voorgeschreven door: __________________________________
☐ nee

7.3 Zijn er producten die u absoluut niet lust en/of niet eet?

☐ ja Namelijk: ____
☐ nee

7.4 Zijn er producten die u niet verdraagt? (bijv. notenallergie, suikerovergevoeligheid of lactose-intolerantie)

☐ ja Namelijk: ____
☐ nee

7.5 Ontbijt u regelmatig ‘s ochtends (5-7 keer per week)?

☐ ja
☐ nee

7.6 Luncht u regelmatig tussen de middag (5-7 keer per week)?

☐ ja
☐ nee

Studiecode TNO: 9334 Keuringsnummer: «preentry»

7.7 Eet u regelmatig ‘s avonds een warme maaltijd (5-7 keer per week)?

☐ ja
☐ nee

7.8 Gebruikt u tussendoortjes?

☐ ja
Wanneer?
- ☐ ‘s morgens
- ☐ ‘s middags
- ☐ ‘s avonds
- ☐ de hele dag door
- ☐ anders, namelijk: ______________________________

☐ nee

Studiecode TNO: 9334 Keuringsnummer: «preentry»

8 Anticonceptie en menstruatie

8.1 Heeft u momenteel een menstruele cyclus?

☐ ja Eerste dag van laatste menstruatie is : |___|___|-|___|___|-|___|___|___|___|
dd mm jjjj

☐ nee Maand/jaar van laatste menstruatie is : |___|___|-|___|___|___|___|
mm jjjj

8.2 Gebruikt u de anticonceptiepil?

☐ nee → **ga verder naar 8.3**
☐ ja

Zoja, welke? (merk pil): ____

Op welke dag (dag van de week en datum) begint u met een nieuwe pilstrip? :

_________dag |___|___|-|___|___|- 2011

Wanneer (dag van de week en datum) heeft u uw volgende stopweek? :

_________dag |___|___|-|___|___|- 2011

8.3 Gebruikt u hormoonvervangende therapie?

☐ ja Namelijk: ____
☐ nee

8.4 Bent u op dit moment zwanger?

☐ ja
☐ nee

8.5 Bent u van plan binnen een half jaar zwanger te worden?

☐ ja
☐ nee

8.6 Geeft u op dit moment borstvoeding?

☐ ja
☐ nee

Studiecode TNO: 9334 Keuringsnummer: «preentry»

In te vullen door kandidaat:

Ondergetekende verklaart dit formulier naar waarheid te hebben ingevuld:

Handtekening: ________________________

Datum (dd-mm-jjjj): |___|___|-|___|___|-|___|___|___|___|

In te vullen door arts:

Ondergetekende, keuringsarts bij TNO, verklaart heden het bovenstaande beoordeeld te hebben en wel / niet* met de kandidaat proefpersoon besproken te hebben, en waar nodig op de daartoe bestemde plaats aanvullende opmerkingen te hebben geplaatst:

Paraaf arts: ________________________

Datum (dd-mm-jjjj): |___|___|-|___|___|-|___|___|___|___|

Opmerkingen:

____

____

____

____

* : doorstrepen hetgeen niet van toepassing is

TNO

Titel formulier: Bevindingen sinds het vorige bezoek

Subject nummer: «entry» Sessie: Datum: __|__|-|_|__|__|-|_2_|_0_|_1_|_1_| (dd-mm-jjjj)

1. Hoe voelt u zich?

____

____

2. Heeft u sinds het vorige bezoek/afgelopen tijd klachten gehad? Ja ☐ Nee ☐

Zo ja, ga naar vraag 3a en /of 3b
Zo nee, ga naar vraag 4

3a. Aard van de klacht ?

____

____

Begindatum klacht: |___|___|-|___|___|-|___|___|___|___| (dd-mm-jjjj)

Einddatum klacht: |___|___|-|___|___|-|___|___|___|___| (dd-mm-jjjj)

Evt. opmerkingen: ____

3b. Eventueel andere klacht: ____________________________________

____

Begindatum klacht: |___|___|-|___|___|-|___|___|___|___| (dd-mm-jjjj)

Einddatum klacht: |___|___|-|___|___|-|___|___|___|___| (dd-mm-jjjj)

Evt. opmerkingen ____

5. Heeft u een arts of tandarts geraadpleegd/bezocht sinds het vorige bezoek? Ja ☐ Nee ☐

Zo ja, waarvoor? ____

Datum bezoek: |___|___|-|___|___|-|___|___|___|___| (dd-mm-jjjj)

TNO

Titel formulier: Bevindingen sinds het vorige bezoek

Subject number: «entry» Sessie: Datum: ___|___|-|_|__|___|-|_2_|_0_|_1_|_1_| (dd-mm-jjjj)

6. Heeft u medicijnen gebruikt sinds het vorige bezoek? Ja ☐ Nee ☐

Zo ja, welke? ________________________________ Dosering: ______________

Reden? ____

____

Begindatum inname: |___|___|-|___|___|-|___|___|___|___| (dd-mm-jjjj)

Einddatum inname: |___|___|-|___|___|-|___|___|___|___| (dd-mm-jjjj)

7. U hoeft deze vraag alleen in te vullen indien de arts hieronder een klacht heeft vermeld.

U vermeldde eerder de klacht: _______________________________________

Is de klacht nog aanwezig? Ja ☐ Nee ☐

Indien nee, wat is de einddatum van de klacht: |___|___|-|___|___|-|___|___|___|___| (dd-mm-jjjj)

Gezien door arts __________(paraaf) |___|___|-|___|___|-|_2_|_0__|_1_|_1_| (dd-mm-jjjj)

Gezien door verpleegkundige __________(paraaf) |___|___|-|___|___|-|_2_|_0_|__1_|_1_| (dd-mm-jjjj)

TNO

Titel formulier: Bevindingen sinds het vorige bezoek

Subject nummer: «entry» Sessie: Datum: ___|___|-|_|__|___|-|_2_|_0_|_1_|_1_| (dd-mm-jjjj)

1. Hoe voelt u zich?

____

____

2. Heeft u sinds u binnenkwam klachten gehad?

Ja ☐ Nee ☐

Zo ja, ga naar vraag 3a en /of 3b
Zo nee, ga naar vraag 4

3a. Aard van de klacht ?

____

____

Begindatum klacht: |___|___|-|___|___|-|___|___|___|___| (dd-mm-jjjj)

Einddatum klacht: |___|___|-|___|___|-|___|___|___|___| (dd-mm-jjjj)

Evt. opmerkingen: ____

3b. Eventueel andere klacht: ____

____

Begindatum klacht: |___|___|-|___|___|-|___|___|___|___| (dd-mm-jjjj)

Einddatum klacht: |___|___|-|___|___|-|___|___|___|___| (dd-mm-jjjj)

Evt. opmerkingen ____

Gezien door arts __________(paraaf) |___|___|-|___|___|-|_2_|_0__|_1_|_1_| (dd-mm-jjjj)

Gezien door verpleegkundige __________(paraaf) |___|___|-|___|___|-|_2_|_0_|__1_|_1_| (dd-mm-jjjj)

TNO Zeist

Titel formulier: **Drinken of niet drinken**

Studiecode TNO: 9334 Keuringsnummer: «preentry»

Drinken of niet drinken

1994, L. Cooper, vertaling/bewerking 2002 R.W. Wiers & P van Empelen, FdP, Universiteit Maastricht

Inleiding

In deze lijst staan redenen die mensen geven om wel of geen alcohol te drinken. Eerst volgt een lijst met redenen die mensen soms geven om alcohol te drinken. Wij vragen u aan te geven hoe vaak u om die reden alcohol drinkt.

Een voorbeeld:

ik drink alcohol ……………………………….

		(bijna) nooit	soms	gemiddeld	vaak	heel vaak
0.	om me stoer te voelen	**0**	**1**	**2**	**3**	**4**

Het gaat er nu dus om aan te geven **<u>hoe vaak</u>** u drinkt om de aangegeven reden.

U kiest wat het meest bij je past door het cijfer achter de zin te omcirkelen.

Als u **nooit of bijna nooit** alcohol drinkt om u stoer te voelen, omcirkelt u de **0**.

Als u **soms** alcohol drinkt om u stoer te voelen, omcirkelt u de **1**.

Als u **ongeveer de helft van de keren dat u alcohol drinkt** dit doet om u stoer te voelen, omcirkelt u de **2**.

Als u **vaak** alcohol drinkt om u stoer te voelen, omcirkelt u de **3**.

Als u **heel vaak of (bijna) altijd** alcohol drinkt om u stoer te voelen, omcirkelt u de **4**.

Ga door op de volgende pagina

TNO Zeist

Titel formulier: **Drinken of niet drinken**

Studiecode TNO: 9334 Keuringsnummer: «preentry»

Alcohol drink ik ……………………

		(bijna) nooit	soms	gemiddeld	vaak	heel vaak
1.	om mijn zorgen even te vergeten ….…………………………….	**0**	**1**	**2**	**3**	**4**
2.	omdat mijn vrienden druk op me uitoefenen om te drinken ……..	**0**	**1**	**2**	**3**	**4**
3.	om van een feest te genieten ....………………………..	**0**	**1**	**2**	**3**	**4**
4.	omdat het me helpt als ik me rot voel …………………………..	**0**	**1**	**2**	**3**	**4**
5.	om gezellig te zijn ……………...................…………………	**0**	**1**	**2**	**3**	**4**
6.	om me beter te voelen als ik een slecht humeur heb . ……….	**0**	**1**	**2**	**3**	**4**
7.	omdat ik me dan lekker voel ………………. …………………	**0**	**1**	**2**	**3**	**4**
8.	zodat anderen me niet belachelijk kunnen maken omdat ik niet drink ………………………………………………………..	**0**	**1**	**2**	**3**	**4**
9.	omdat het me een “kick” geeft ……………………..…………..……	**0**	**1**	**2**	**3**	**4**
10.	om lekker aangeschoten te raken (“high”) ………..………………..	**0**	**1**	**2**	**3**	**4**
11.	omdat het sociale gelegenheden leuker maakt ……...............……..	**0**	**1**	**2**	**3**	**4**
12.	om bij een groep te horen die ik leuk vind ………………………..	**0**	**1**	**2**	**3**	**4**
13.	omdat ik me dan vrolijk voel ………..………………..………….	**0**	**1**	**2**	**3**	**4**
14.	omdat het feestjes en uitgaan leuker maakt ..……………………..	**0**	**1**	**2**	**3**	**4**
15.	om me zeker van mezelf te voelen …………………………………	**0**	**1**	**2**	**3**	**4**
16.	om iets speciaals te vieren ……………..…………………………..	**0**	**1**	**2**	**3**	**4**
17.	om mijn problemen te vergeten …………………………………….	**0**	**1**	**2**	**3**	**4**
18.	omdat het leuk is ….…. ………………………………………….	**0**	**1**	**2**	**3**	**4**
19.	om aardig gevonden te worden …………………………………….	**0**	**1**	**2**	**3**	**4**
20.	om me niet buitengesloten te voelen ……………….………………	**0**	**1**	**2**	**3**	**4**

dank voor het invullen

Score__________ (paraaf) ________(datum)__________ Controle: (paraaf)_________datum)___________

TNO Zeist

Titel formulier: **Stress Vragenlijst** (**State Trait Anxiety Inventory – Trait anxiety)**

Studiecode TNO: 9334 Keuringsnummer: «preentry»

Instructies Hieronder vindt u een aantal uitspraken, die door mensen kunnen gebruikt om zichzelf te beschrijven. **Lees iedere uitspraak door en zet een kruisje in het hokje rechts van die uitspraak om daarmee aan te geven hoe u zich *in het algemeen* voelt.** Er zijn geen goede of slechte antwoorden. Denk niet te lang na en geef uw eerste indruk. Het gaat er dus om dat u bij deze vragenlijst weergeeft hoe u zich *in het algemeen* voelt.

	BIJNA NOOIT	SOMS	VAAK	BIJNA ALTIJD
Ik voel me prettig	☐	☐	☐	☐
Ik voel me nerveus en onrustig	☐	☐	☐	☐
Ik voel me tevreden	☐	☐	☐	☐
Ik kan een tegenslag maar heel moeilijk verwerken	☐	☐	☐	☐
Ik voel me in vrijwel alles tekort schieten	☐	☐	☐	☐
Ik voel me uitgerust	☐	☐	☐	☐
Ik voel me rustig en beheerst	☐	☐	☐	☐
Ik voel dat de moeilijkheden zich opstapelen zodat ik er niet meer tegenop kan	☐	☐	☐	☐
Ik pieker teveel over dingen die niet zo belangrijk zijn	☐	☐	☐	☐
Ik ben gelukkig	☐	☐	☐	☐
Ik wordt geplaagd door storende gedachten	☐	☐	☐	☐
Ik heb gebrek aan zelfvertrouwen	☐	☐	☐	☐
Ik voel me veilig	☐	☐	☐	☐
Ik voel me op mijn gemak	☐	☐	☐	☐
Ik ben gelijkmatig van stemming	☐	☐	☐	☐
Ik ben tevreden	☐	☐	☐	☐
Er zijn gedachten die ik heel moeilijk los kan laten	☐	☐	☐	☐
Ik neem teleurstellingen zo zwaar op dat ik ze niet van me af kan zetten	☐	☐	☐	☐
Ik ben een rustig iemand	☐	☐	☐	☐
Ik raak helemaal gespannen en in beroering als ik denk aan mijn zorgen van de laatste tijd	☐	☐	☐	☐

Score__________ (paraaf) ________(datum)__________ Controle: (paraaf)_________datum)___________

TNO Zeist

Titel formulier: **Nederlandse vragenlijst voor eetgedrag**

Studiecode TNO: 9334 Keuringsnummer: «entry»

Instructie:
Op de volgende bladzijden vindt u een aantal vragen over eetgewoonten. Lees elke vraag goed door en zet dan een kruisje in het hokje bij het antwoord dat het beste bij u past. Per vraag één antwoord.
Uw eerste indruk is meestal de beste; staat u dus niet te lang stil bij elke vraag. Elk antwoord dat u geeft is goed.

1. Als u geïrriteerd bent, hebt u dan zin om iets te eten?

☐ nooit
☐ zelden
☐ soms
☐ vaak
☐ zeer vaak
☐ ik ben **nooit** geïrriteerd

2. Als het eten u goed smaakt, eet u dan meer dan u gewend bent?

☐ nooit
☐ zelden
☐ soms
☐ vaak
☐ zeer vaak

3. Als u niets te doen heeft, krijgt u dan zin om iets te eten?

☐ nooit
☐ zelden
☐ soms
☐ vaak
☐ zeer vaak
☐ ik heb **altijd** wel wat te doen

4. Als u wat zwaarder bent geworden, eet u dan daarna wat minder dan u gewend bent?

☐ nooit
☐ zelden
☐ soms
☐ vaak
☐ zeer vaak
☐ ik wordt **nooit** zwaarder

5. Als u terneergeslagen of ontmoedigd bent, hebt u dan zin om iets te eten?

☐ nooit
☐ zelden
☐ soms
☐ vaak
☐ zeer vaak
☐ ik ben **nooit** terneergeslagen of ontmoedigd

Titel formulier: **Nederlandse vragenlijst voor eetgedrag**

Studiecode TNO: 9334 Keuringsnummer: «entry»

6. Als het eten lekker ruikt en er goed uit ziet, neemt u dan een grotere portie dan u gewend bent?
   - ☐ nooit
   - ☐ zelden
   - ☐ soms
   - ☐ vaak
   - ☐ zeer vaak

7. Hoe vaak slaat u aangeboden eten en drinken af omdat u aan uw gewicht wilt denken?
   - ☐ nooit
   - ☐ zelden
   - ☐ soms
   - ☐ vaak
   - ☐ zeer vaak

8. Als u zich alleen voelt, hebt u dan zin om iets te eten?
   - ☐ nooit
   - ☐ zelden
   - ☐ soms
   - ☐ vaak
   - ☐ zeer vaak
   - ☐ ik voel me **nooit** alleen

9. Als u iets lekkers ziet of ruikt, krijgt u dan trek?
   - ☐ nooit
   - ☐ zelden
   - ☐ soms
   - ☐ vaak
   - ☐ zeer vaak

10. Als u zich in de steek gelaten voelt, hebt u dan zin om iets te eten?
    - ☐ nooit
    - ☐ zelden
    - ☐ soms
    - ☐ vaak
    - ☐ zeer vaak
    - ☐ ik voel me **nooit** in de steek gelaten

11. Komt het voor dat u tijdens de maaltijden minder probeert te eten dan u wel zou lusten?
    - ☐ nooit
    - ☐ zelden
    - ☐ soms
    - ☐ vaak
    - ☐ zeer vaak

Titel formulier: **Nederlandse vragenlijst voor eetgedrag**

Studiecode TNO: 9334 Keuringsnummer: «entry»

12. Als u iets lekkers heeft, eet u dat dan meteen op?
☐ nooit
☐ zelden
☐ soms
☐ vaak
☐ zeer vaak

13. Als u boos bent, hebt u dan zin om iets te eten?
☐ nooit
☐ zelden
☐ soms
☐ vaak
☐ zeer vaak

14. Let u er precies op hoeveel u eet?
☐ nooit
☐ zelden
☐ soms
☐ vaak
☐ zeer vaak

15. Als u langs een bakker loopt, krijgt u dan zin om iets lekkers te kopen?
☐ nooit
☐ zelden
☐ soms
☐ vaak
☐ zeer vaak

16. Als u iets onprettigs te wachten staat, hebt u dan zin om iets lekkers te kopen?
☐ nooit
☐ zelden
☐ soms
☐ vaak
☐ zeer vaak

17. Eet u met opzet dingen, die goed zijn voor de lijn?
☐ nooit
☐ zelden
☐ soms
☐ vaak
☐ zeer vaak

18. Als u andere mensen ziet eten, krijgt u dan ook zin?
☐ nooit
☐ zelden
☐ soms
☐ vaak
☐ zeer vaak

TNO Zeist

Titel formulier: **Nederlandse vragenlijst voor eetgedrag**

Studiecode TNO: 9334 Keuringsnummer: «entry»

19. Als u teveel hebt gegeten, eet u dan de dag daarna minder dan u gewend bent?
    - ☐ nooit
    - ☐ zelden
    - ☐ soms
    - ☐ vaak
    - ☐ zeer vaak
    - ☐ ik eet **nooit** teveel

20. Als u ongerust, bezorgd of gespannen bent, hebt u dan zin om iets te eten?
    - ☐ nooit
    - ☐ zelden
    - ☐ soms
    - ☐ vaak
    - ☐ zeer vaak
    - ☐ ik ben **nooit** ongerust, bezorgd of gespannen

21. Kunt u van lekkere dingen afblijven?
    - ☐ nooit
    - ☐ zelden
    - ☐ soms
    - ☐ vaak
    - ☐ zeer vaak

22. Eet u om niet dikker te worden met opzet wat minder?
    - ☐ nooit
    - ☐ zelden
    - ☐ soms
    - ☐ vaak
    - ☐ zeer vaak

23. Als u iets tegenzit of verkeerd gaat, hebt u dan zin om iets te eten?
    - ☐ nooit
    - ☐ zelden
    - ☐ soms
    - ☐ vaak
    - ☐ zeer vaak

24. Als u langs een snackbar of cafetaria loopt, krijgt u dan zin om iets lekkers te kopen?
    - ☐ nooit
    - ☐ zelden
    - ☐ soms
    - ☐ vaak
    - ☐ zeer vaak

Titel formulier: **Nederlandse vragenlijst voor eetgedrag**

Studiecode TNO: 9334 Keuringsnummer: «entry»

25. Als u opgewonden bent, hebt u dan zin om iets te eten?
☐ nooit
☐ zelden
☐ soms
☐ vaak
☐ zeer vaak
☐ ik ben **nooit** opgewonden

26. Hoe vaak probeert u tussen de maaltijden door niets te eten omdat u aan de lijn doet?
☐ nooit
☐ zelden
☐ soms
☐ vaak
☐ zeer vaak

27. Eet u meer dan u gewend bent, als u anderen ziet eten?
☐ nooit
☐ zelden
☐ soms
☐ vaak
☐ zeer vaak

28. Als u zich verveelt, of rusteloos voelt, hebt u dan zin om iets te eten?
☐ nooit
☐ zelden
☐ soms
☐ vaak
☐ zeer vaak
☐ ik voel me **nooit** verveeld of rusteloos

29. Hoe vaak probeert u in de loop van de avond niets te eten, omdat u aan de lijn doet?
☐ nooit
☐ zelden
☐ soms
☐ vaak
☐ zeer vaak

30. Als u angstig bent, krijgt u dan zin om iets te eten?
☐ nooit
☐ zelden
☐ soms
☐ vaak
☐ zeer vaak
☐ ik ben **nooit** angstig

Titel formulier: **Nederlandse vragenlijst voor eetgedrag**

Studiecode TNO: 9334 Keuringsnummer: «entry»

31. Houdt u met eten rekening met uw gewicht?
☐ nooit
☐ zelden
☐ soms
☐ vaak
☐ zeer vaak

32. Als u zich teleurgesteld voelt, hebt u dan zin om iets te eten?
☐ nooit
☐ zelden
☐ soms
☐ vaak
☐ zeer vaak
☐ ik ben **nooit** teleurgesteld

33. Bent u geneigd om tijdens het bereiden van een maaltijd alvast wat te eten?
☐ nooit
☐ zelden
☐ soms
☐ vaak
☐ zeer vaak

TNO Zeist

Titel formulier: **Eind vragenlijst alcoholpercentage**

Studiecode TNO: 9334 Keuringsnummer: «entry»

Hoe groot schat je het alcoholpercentage van de wijnen die je tijdens de verschillende testdagen hebt gedronken?

1^{e} testdag: …………….%

2^{e} testdag: …………….%

3^{e} testdag: …………….%

4^{e} testdag: …………….%

Datum: ____
